# A genome-wide association study identifies new loci associated with response to SARS-CoV-2 mRNA-1273 vaccine in a cohort of healthy healthcare workers

**DOI:** 10.3389/fimmu.2025.1639825

**Published:** 2025-08-18

**Authors:** Antonio José Cabrera-Serrano, Lucía Ruiz-Durán, Juan Francisco Gutiérrez-Bautista, María Carretero-Fernández, Rob ter Horst, Yang Li, Fernando Jesús Reyes-Zurita, Francisco José García-Verdejo, Mihai G. Netea, Pedro Sánchez-Rovira, Miguel Ángel López-Nevot, Antonio Sampedro, Juan Sainz

**Affiliations:** ^1^ Genomic Oncology Area, GENYO, Centre for Genomics and Oncological Research: Pfizer/University of Granada/Andalusian Regional Government, Parque Tecnológico de Ciencias de la Salud (PTS), Granada, Spain; ^2^ Instituto de Investigación Biosanitaria IBs.Granada, Granada, Spain; ^3^ Servicio de Análisis Clínicos e Inmunología, University Hospital Virgen de las Nieves, Granada, Spain; ^4^ Departamento de Bioquímica, Biología Molecular e Inmunología III, University of Granada, Granada, Spain; ^5^ Department of Internal Medicine and Radboud Center for Infectious Diseases, Radboud University Nijmegen Medical Center, Nijmegen, Netherlands; ^6^ CeMM Research Center for Molecular Medicine of the Austrian Academy of Sciences, Vienna, Austria; ^7^ Medical University of Vienna, Center for Medical Data Science, Institute of Artificial Intelligence, Vienna, Austria; ^8^ Centre for Individualised Infection Medicine (CiiM) & TWINCORE, joint ventures between the Helmholtz-Centre for Infection Research (HZI) and the Hannover Medical School (MHH), Hannover, Germany; ^9^ Department of Biochemistry and Molecular Biology I, Faculty of Sciences, University of Granada, Granada, Spain; ^10^ Department of Medical Oncology, Complejo Hospitalario de Jaén, Jaén, Spain; ^11^ Department for Immunology & Metabolism, Life and Medical Sciences Institute (LIMES), University of Bonn, Bonn, Germany; ^12^ Servicio de Microbiología, University Hospital Virgen de las Nieves, Granada, Spain; ^13^ Centro de Investigación Biomédica en Red de Epidemiología y Salud Pública (CIBERESP), Madrid, Spain

**Keywords:** SARS-CoV-2, GWAS, circulating IgG levels, genetic variants, IgG decay

## Abstract

**Introduction:**

The COVID-19 pandemic had significant global public health consequences, affecting over 200 countries and regions by 2020. The development and efficacy of specific vaccines, such as the mRNA-1273 (Spikevax^®^) vaccine developed by Moderna Inc., have substantially reduced the impact of the pandemic and mitigated its consequences. This study aims to identify novel genetic loci associated with the effectiveness of the mRNA-1273 vaccine, as measured by elevated anti-Spike (anti-S) IgG levels at multiple time points post-vaccination.

**Materials and methods:**

We conducted three genome-wide association studies (GWAS) in a cohort of Spanish healthcare workers, analyzing anti-S IgG levels at one-month post-vaccination (n=567), at three months post-vaccination (n=447), and the difference in circulating anti-S IgG levels between these two time points (n=447).

**Results:**

We identified fourteen novel loci associated with increasing concentrations of anti-S IgG post-vaccination (*p*=5.01×10^-13^ and *p*=2.81×10^-8^). Functional results showed that some of the novel risk alleles influence the absolute counts of specific B cell subsets (*p*=2.57×10^-5^-8.82×10^-3^), which are involved in immune signaling pathways and metabolic processes. Furthermore, these variants co-localize with multiple QTLs and epigenetic marks, suggesting that the GWAS hits may affect regulatory activity in promoters, enhancers, and transcriptional regions, thereby modulating gene expression relevant to the humoral immune response.

**Discussion:**

In conclusion, this study highlights the complex interplay of genetic factors influencing the immune response to vaccination, particularly through modulation of B cell activity, immune signaling pathways, and metabolic processes. The identification of genetic variants could inform future strategies to enhance vaccine efficacy and provide a deeper understanding of individual variability in vaccine responses, especially for COVID-19 and other viral infections.

## Introduction

The SARS-CoV-2 pandemic had a profound impact on the global population. As of June 2020, the virus spread to over 200 countries and territories, affecting diverse populations and communities (1). Since the emergence of the virus in 2019, more than 775 million infections and about 7 million deaths have been reported because of this infection (2), although these numbers are likely to be underestimated.

Advanced age and the presence of comorbidities such as hypertension, diabetes, and cardiovascular disease have been identified as significant risk factors for severe complications from SARS-CoV-2 infection (3). These underlying conditions can increase the vulnerability of individuals to the virus, as it enters host cells through the angiotensin-converting enzyme 2 (ACE2) receptor, which is present in a wide range of tissues and organs (4). Additionally, chronic lung diseases, including chronic obstructive pulmonary disease and interstitial lung diseases such as idiopathic pulmonary fibrosis, have been associated with a higher risk of hospitalization and mortality among SARS-CoV-2 patients (5). The evasion from the effects of the type I interferon response by SARS-CoV-2 is another critical factor contributing to its ability to cause severe disease (6). This immune evasion mechanism allows the virus to replicate and spread more efficiently, leading to the development of acute respiratory distress syndrome and other life-threatening complications (6).

Given the significant morbidity and mortality, the development and distribution of effective and safe vaccines have become a top priority (7). Vaccination efforts against SARS-CoV-2 have been rapid and widespread, with a significant proportion of the global population having received at least one vaccine dose. These vaccines, including those based on messenger RNA (mRNA), viral vectors, recombinant proteins, and inactivated virus platforms, primarily aim to generate a humoral immune response against the SARS-CoV-2 spike (S) protein, which plays a crucial role in the ability of the virus to infect host cells (8).

Numerous studies have investigated the response of different populations to SARS-CoV-2 vaccines (8–12). The immune response to the vaccines has been found to be generally robust in the general population, with the vaccines demonstrating high efficacy in reducing the risk of severe disease and hospitalization (8, 12). One of the most widely used SARS-CoV-2 vaccines is the mRNA-1273 vaccine (Spikevax^®^), developed by Moderna Inc. in collaboration with the National Institute of Allergy and Infectious Diseases (11). The mRNA-1273 vaccine, similar to the BNT162b2 vaccine developed by BioNtech and Pfizer, utilizes mRNA technology to enable the production of the full-length SARS-CoV-2 S protein, which triggers an immune response (10) While these mRNA vaccines have been shown to be highly effective in preventing SARS-CoV-2 infections and severe disease, there is growing interest in understanding the factors that may influence individual responses to these vaccines (9). Some studies have linked humoral and cellular responses to mRNA vaccination to human leukocyte antigen (HLA) molecules (13–15). However, relatively few studies have explored the association between genetic polymorphisms and vaccine response. Genome-wide association studies (GWAS) have emerged as a powerful tool for identifying genetic variants linked to various traits and diseases, including immune responses to vaccines (16, 17). With respect to SARS-CoV-2 infection, numerous studies have identified polymorphisms related to severity, infection, and disease susceptibility (18–20), but only a few have shown alterations in IgG responses and altered cytokine profiles post-vaccination (21–23).

Understanding the genetic factors that influence vaccine response is crucial for optimizing vaccination strategies and identifying individuals at risk of suboptimal immune responses. By elucidating the genetic architecture underlying the response to the mRNA-1273 vaccine, this study aims to provide insights that can facilitate and accelerate the development of improved vaccination approaches, contribute to ongoing efforts to combat SARS-CoV-2, and enhance our understanding of the response to mRNA vaccines. To this end, we conducted, for the first time, a GWAS involving 601 Spanish healthcare workers, correlating their genetic data with their humoral immune response to the mRNA-1273 vaccine.

## Materials and methods

### Study population

The study included 601 healthy healthcare workers (399 women and 202 men) recruited from the Virgen de las Nieves University Hospital in Granada, Spain. All participants received the mRNA-1273 vaccine (Moderna). Eligibility criteria required the absence of prior SARS-CoV-2 infection, verified through review of clinical history, RT-PCR testing, and institutional serological screenings. Individuals with any previous positive PCR or serological result were excluded. A follow-up antibody determination was performed at 30 and 90 days after vaccination.

All biological samples were collected in accordance with local medical ethics regulations, following the provision of informed consent by the participants, their legal representatives, or both, in line with guidelines reported in the Declaration of Helsinki. The study protocol was reviewed and approved by the regional ethics committee (Portal de Ética de la Investigación Biomédica, Junta de Andalucía, code: 0297-N-21).

### Antibodies against SARS‐CoV‐2 quantification

Participants underwent blood extraction at 1 month (30 days) and 3 months (90 days) after receiving the second vaccine dose of the mRNA-1273 vaccine. Quantitative determination of IgG antibodies against the SARS-CoV-2 Spike (S) protein was performed. Circulating anti-Spike IgG levels were quantified using chemiluminescent SARS-CoV-2 IgG assay (Alinity, Abbott, USA), following the manufacturer’s instructions. Results are expressed in binding antibody units per milliliter (BAU/mL), with a positivity cutoff set at >7.5 BAU/mL.

It is important to note that anti-Spike IgG levels are used in this study as a surrogate marker of the humoral immune response. Although such measurements provide a standardized and widely accepted indication of prior immunologic exposure or response to vaccination, they do not directly measure neutralizing antibody activity or functional immune protection. Therefore, the results should be interpreted within the context of known limitations of binding antibody assays, and not as definitive correlates of vaccine-induced clinical protection against SARS-CoV-2 infection.

### DNA extraction and quantification

All blood samples were stored at −80°C until analysis. DNA extraction was carried out at GENYO (Centre for Genomics and Oncological Research: Pfizer/University of Granada/Andalusian Regional Government, Granada, Spain) using QIAamp DNA Blood kits (Valencia, CA, USA) according to the manufacturer’s instructions. DNA concentration and quality were measured using Qubit 4 Fluorometer (Thermo Fisher, Applied Biosystems, Waltham MA, USA).

### Genome-wide associations analyses

All individuals included in the GWAS were genotyped using the Infinium™ Global Screening Array-24 v3.0 BeadChip (Illumina, San Diego, CA, USA). Extensive quality control metrics were applied to the data using R v3.3.1 and PLINK v1.90p software. Samples were excluded if there was sex discordance, a call rate of <90%, minimal or excessive heterozygosity (>3 SDs from the mean), estimated relatedness (Pihat≥0.2) or if they were identified as non-European based on principal components analysis (PCA). The PCA was performed with PLINK v1.90b, including the genotypes from phase 3 of the 1000 Genomes Project as the reference panel (24).

Genetic variants were excluded if they showed a significant deviation from Hardy–Weinberg equilibrium (HWE<1×10^-5^), had minor allele frequency (MAF) of <0.05, or a genotype call rate of ≤90%. Genome-wide imputation was then performed using the Michigan Imputation Server (https://imputationserver.sph.umich.edu/index.html) and the Haplotype Reference Consortium reference haplotype panel (HRC V.r1.1; http://www.haplotype-reference-consortium.org/) (25). All variants with an imputation R^2^<0.3 were excluded from subsequent analysis. PLINK v1.90p was used to perform all GWAS analysis. To identify independent SNPs, we utilized data from LDLink for European cohorts (https://ldlink.nih.gov/?tab=home). SNPs were considered independent if they met the criteria of r²<0.1 and D’<0.2.

To ensure the accuracy and comparability of the genetic association analysis, the IgG phenotypes were normalized using Z-score transformation prior to conducting the GWAS. We conducted linear regression analysis to examine the association between SNPs and circulating IgG levels, adjusting for sex, age and the top ten principal components. Genomic control (GC) adjustment to ensure the robustness and validity of our analysis.

We conducted three GWAS to investigate genetic factors associated with IgG levels at different time points. Specifically, the first GWAS focused on IgG quantification measured during the first month, while the second analysis assessed IgG levels at the third month. A third GWAS was performed to analyze the absolute differences in IgG levels between these two time points. This design allowed us to capture both static and dynamic changes in IgG levels over time, enabling the identification of genetic variants potentially influencing baseline IgG production, temporal changes, and overall immune dynamics.

### Functional effect of GWAS hits and cytokine quantitative trait loci, circulating levels of inflammatory proteins, blood-derived cell populations, and steroid hormones

To provide insight into the functional role of the novel SNPs identified through the GWAS, we performed *in vitro* stimulation experiments and measured cytokine production (IFNγ, IL1Ra, IL1β, IL6, IL8, IL10, TNFα, IL17, and IL22) after stimulation of peripheral blood mononuclear cells (PBMCs), whole blood (WB) or monocyte-derived macrophages (MDMs) with LPS (1 or 100 ng/ml), PHA (10μg/ml), Pam3Cys (10μg/ml), CpG (ODN M362; 10μg/ml), *Escherichia coli*, and *Staphylococcus aureus*. Stimulation experiments were conducted on 408 healthy subjects of the 500FG of the Human Functional Genomics Project (HFGP) according to previously reported protocols (26, 27).

A proteomic analysis was also performed on serum and plasma samples from the 500FG cohort. Circulating protein concentrations were measured using the commercial Olink^®^ Inflammation panel (Olink, Sweden), resulting in the quantification of 103 different biomarkers (**Supplementary Table 1**). Protein concentrations were expressed on a log_2_ scale as normalized protein expression values and were further normalized using bridging samples to correct for batch variation (27).

Additionally, we tested the association of GWAS hits with absolute numbers of 91 blood-derived cell populations (**Supplementary Table 2**). Blood-derived cell populations were measured by 10-color flow cytometry (Navios flow cytometer, Beckman Coulter, Miami, FL, USA) after blood sampling (2–3 h), and cell count analysis was performed using Kaluza software (Beckman Coulter, v.1.3). To reduce inter-experimental noise and increase statistical power, cell count analysis was performed by calculating parental and grandparental percentages, which were defined as the percentage of a certain cell type within the subpopulation of the cells from which it was isolated. Detailed laboratory protocols for cell isolation, reagents, gating strategies, and flow cytometry analysis, as well as methodological details of the functional experiments, have been reported elsewhere (28, 29).

Given the impact of sex on the response to mRNA vaccines for SARS-CoV-2A, as well as the influence of steroid hormones on immune responses, we also evaluated the association of GWAS markers with circulating concentrations of seven steroid hormones (androstenedione, cortisol, 11-deoxy-cortisol, 17-hydroxy-progesterone, progesterone, testosterone and 25-hydroxy vitamin D3) in a subset of the 500FG cohort, excluding individuals undergoing hormonal replacement therapy or taking oral contraceptives (n=279) (27).

Finally, in order to test if genetic markers were associated with baseline serum levels of immunoglobulin IgG and its subclasses were measured by immunonephelometry using Beckman Coulter reagents and a Beckman Coulter Imager according to previously reported protocols.

In order to account for multiple comparisons, we used a significance threshold of 4.62×10^−4^ (0.05/12 SNPs/9 cytokines), *p*=4.04×10^-5^ (0.05/12 SNPs/103 inflammatory proteins, *p*=4.58×10^-5^ (0.05/12 SNPs/91 blood cell types), 5.95×10^-4^ (0.05/12 SNPs/7 hormones) 4.16×10^-3^ (0.05/12 SNPs IgG levels) for the cytokine quantitative trait loci, proteomic, blood cell counts, steroid hormone analyses and IgG levels, respectively. All functional analyses were performed using R v4.2.2 software (https://www.r-project.org/) adjusted by age and sex as covariates, using custom scripts in the R programming language based on existing functions. Functional plots were displayed using Graphpad Prism v8.0.1 (Graphpad Software, San Diego, CA, USA). All data used in this project have been meticulously cataloged and archived in the BBMRI-NL data infrastructure (https://hfgp.bbmri.nl/) using the MOLGENIS open-source platform for scientific data (30). This allows flexible data querying and download, including sufficiently rich metadata and interfaces for machine processing (R statistics, REST API) and using FAIR principles to optimize Findability, Accessibility, Interoperability, and Reusability (31).

### Bioinformatic and in silico analyses

Annotation and biological interpretation of genome-wide significant association results were performed using publicly available bioinformatic tools, including the FUMA-GWAS platform (https://fuma.ctglab.nl/) (32) and the Open Targets Platform (https://platform.opentargets.org/) (33). We also tested whether the associated SNPs could represent expression quantitative trait loci (eQTL) acrossdifferent cell types and tissues using data from the GTEx portal (https://gtexportal.org/home/) (34) and QTLbase (http://www.mulinlab.org/qtlbase) (35), which aggregates functional QTL data from sources such as TCGA, GTEx, Pancan-MNVQTLdb, and DICE. To complete these functional analyses, meta-scores were developed, integrating diverse annotations or individual scores into a single quantitative score using Combined Annotation Dependent Depletion (CADD) (36) and Regulome DB (https://regulomedb.org/) (37).

These in silico analyses were conducted in an exploratory framework, and we emphasize that they do not constitute direct experimental validation.Annotations were considered of potential interest if they met a nominal significance threshold (p<1×10^-3^) and/or were supported by at least two independent tools among FUMA-GWAS, Open Targets Platform, GTEx, QTLbase, CADD, and RegulomeDB, in order to increase the reliability of the functional prioritization. The GC value was calculated using the “QCEWAS” (38) package from R v4.2.2 software to estimate the inflation rate for each GWAS. Quantile-quantile (Q-Q) plots and Manhattan plots were generated using “qqplot” (39) and “qqman” (40) procedures in R v3.3.1.

## Results

A total of 601 healthy healthcare workers were recruited at the Virgen de las Nieves University Hospital (Granada, Spain), including 399 women and 202 men. After applying quality control filters, antibody determination during the first 30 days was conducted in a cohort of 567 individuals (366 women and 201 men). The overall mean age was 48.1 years (range: 21-68 years), with a mean of 48.8 years for women (range: 21-66 years) and 46.6 years for men (range: 23-68 years). At the time of the second antibody determination, conducted 90 days after vaccination, 447 participants were included (294 women and 153 men). The mean age was 49.5 years (range: 22–68 years), with 50.0 years for men (range: 22–66) and 47.8 years for women (range: 23–68 years) (**Table 1**). While most participants showed a decline in IgG levels between the first and second determinations, only 2 individuals (0.45%) exhibited increased IgG titers at 90 days, indicating minimal upward variation at the individual level.

**Table 1:** Description of study cohorts.

Variable	*Cohort for 1 month (N=567)*	*Cohort for 3 months (N=447)*	*Cohort for 1-3 months (N=447)*
*Age (years)*	48.19 ± 11.49	49.50 ± 11.10	49.50 ± 11.10
*Sex ratio (female/male)*	1.82 (366/201)	1.92 (294/153)	1.92 (294/153)
Anti-S IgG (BAU/mL)*	2424.31 (1543.41-3891.87)	892.09 (520.63-1469.24)	1526.62 (998.3-2401.49)

*Values are presented as Median (IQR). IQR, Interquartile Range.

We also evaluated the potential effect of age and sex on IgG concentrations at both time points (**Supplementary Table 3**). Sex had a statistically significant impact on antibody levels: men showed higher median IgG titers than women at both 1 month (3570.5 vs. 2697.3 AU/mL; p = 0.00079) and 3 months (1305.8 vs. 991.6 AU/mL; p = 0.00048) post-vaccination. A slight difference in age between sexes was observed at 1 month (p = 0.035), but not at 3 months (p = 0.309), and we found no strong overall correlation between age and IgG levels. Based on these findings, all GWAS analyses were adjusted for age and sex to account for their potential confounding effects.

### Genome-wide genetic analyses

After quality control, three GWAS were conducted. The first included a total of 567 individuals and corresponded to data collected one month after vaccination. The second GWAS included 447 individuals, a subset of the original cohort, and was based on data collected three months after vaccination. The third GWAS, also with 447 individuals, compared antibody responses between 1- and 3-months post-vaccination. The GC factor (lambda) was adjusted to 1.001 for all GWAS, indicating minimal inflation of test statistics due to population structure. The Q-Q plot did not show evidence of systematic inflation (**Supplementary Figures 1A–C**), confirming the absence of hidden population substructure or cryptic relatedness.

### GWAS at 1-month post-vaccination

**Figure 1:**
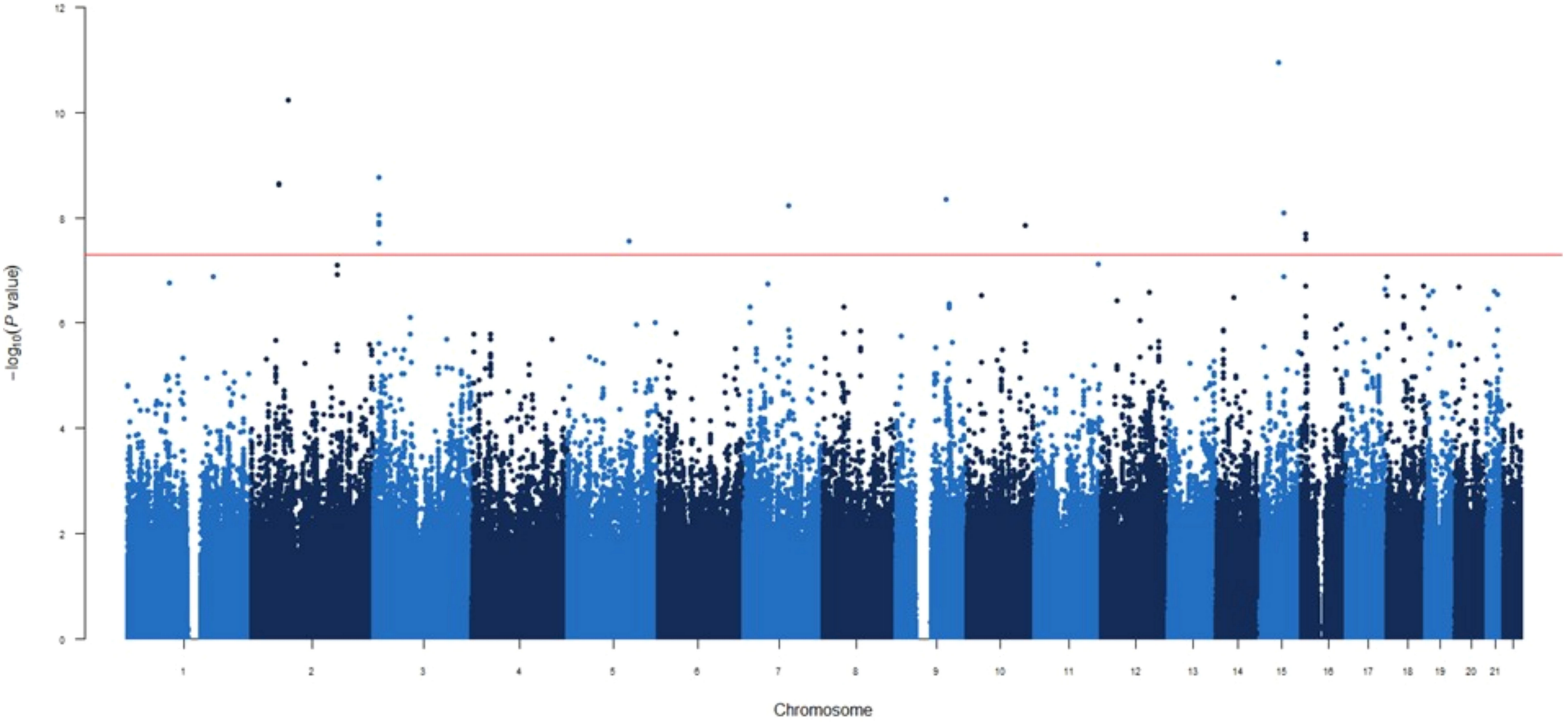
Manhattan plot for the GWAS analysis for IgG levels measured one month after mRNA-1273 vaccine.

In the GWAS at 1-month post-vaccination, nine novel independent genetic signals were identified associated with circulating IgG concentrations (**Table 2**, **Figure 1**).

**Table 2:** Genome-wide significant associations with antibody production identified in GWASs.

GWASs	SNP	Chr	Position	Alt/Ref	Nearest gene(s)	Consequence	MAF	MAF (1,000 Genomes)	Beta	Standard error	P_value_
GWAS 1	rs1350209880	15	58183817	A/T	*ENSG00000295231* | *ALDH1A2*	LncRNA | Upstream gene variant	0.060	0.00004	0.785	0.113	1.12×10^-11^
	rs72845602 δ	2	72347224	T/C	*CYP26B1*	Downstream gene variant	0.053	0.040	0.845	0.126	5.72×10^-11^
	rs4088054 γ	3	10808633	T/A	*SLC6A11* | *LINC00606*	Intergenic | Upstream gene variant	0.093	0.088	0.654	0.106	1.71×10^-09^
	rs117643807	9	100352774	T/C	*TMOD1*	Intron variant	0.068	0.067	0.724	0.121	4.37×10^-09^
	rs7792239	7	90808856	A/G	*CDK14*	Intron variant	0.067	0.105	0.773	0.130	5.93×10^-09^
	rs28485994	15	67257395	C/T	*SMASR* | *SMAD3-DT*	Intergenic variant	0.097	0.094	0.588	0.100	8.13×10^-09^
	rs7907582	10	118144424	C/G	*CCDC172* | *PNLIPRP3*	Intergenic variant	0.069	0.087	0.674	0.117	1.41×10^-08^
	rs34340658	16	10129731	T/C	*GRIN2A* | *LOC105371076*	Intron variant	0.124	0.095	0.539	0.094	2.04×10^-08^
	rs55770715	5	122347325	A/G	*SNX24* | *LOC124901213*	Intergenic variant	0.061	0.041	0.738	0.130	2.78×10^-08^
GWAS 2	rs28485994	15	67257395	C/T	*SMASR* | *SMAD3-DT*	Intergenic variant	0.097	0.094	0.768	0.110	3.66×10^-12^
	rs55725269 γ	3	10799545	A/G	*ATP2B2*	Downstream gene variant	0.057	0.054	0.812	0.140	7.62×10^-09^
GWAS 3	rs1350209880	15	58183817	A/T	*ENSG00000295231* | *ALDH1A2*	LncRNA | Upstream gene variant	0.060	0.00004	0.915	0.122	5.01×10^-13^
	rs117643807	9	100352774	T/C	*TMOD1*	Intron variant	0.068	0.067	0.932	0.139	1.04×10^-10^
	rs75197984 δ	2	54765683	T/C	*SPTBN1*	Intron variant	0.059	0.063	0.863	0.131	1.97×10^-10^
	rs7907582	10	118144424	C/G	*CCDC172* | *PNLIPRP3*	Intergenic variant	0.069	0.087	0.779	0.126	2.15×10^-09^
	rs7792239	7	90808856	A/G	*CDK14*	Intron variant	0.067	0.105	0.873	0.145	5.65×10^-09^
	rs1125991	2	172263448	A/T	*METTL8*	Intron variant	0.083	0.072	0.706	0.118	7.71×10^-09^
	rs34340658	16	10129731	T/C	*GRIN2A* | *LOC105371076*	Intron variant	0.124	0.095	0.669	0.112	8.64×10^-09^
	rs4630616	17	76330484	A/G	*LOC105371912*	Intron variant	0.153	0.176	0.626	0.107	1.78×10^-08^
	rs55919500	20	8072501	A/G	*PLCB1*	Intron variant	0.065	0.101	0.805	0.140	2.81×10^-08^

SNP, single nucleotide polymorphism; Chr, Chromosome; Alt, alternative allele; Ref, reference allele; MAF, minor allele frequency; OR, odds ratio; CI, confidence interval; P, P-value; Phet, P-value of heterogeneity.

^δ^rs72845602 and rs75197984 are in modest linkage disequilibrium (D’ = 0.601; r^2^ = 0.001).

^γ^rs4088054 and rs55725269 are in complete linkage disequilibrium with the rs3745990 (D’ = 1.00; r^2^ = 0.584).

GWAS 1: The GWAS analysis of IgG levels measured at the first month after mRNA-1273 vaccination.

GWAS 2: The GWAS analysis of IgG levels measured at the third month after mRNA-1273 vaccination.

GWAS 3: The GWAS analysis of the difference in IgG levels between the first and third months after mRNA-1273 vaccination.

GWAS analyses were conducted using linear regression with PLINK software. Estimates calculated according to a log-additive model of inheritance and adjusted for age, sex and 10 first principal components.

**Figure 2:**
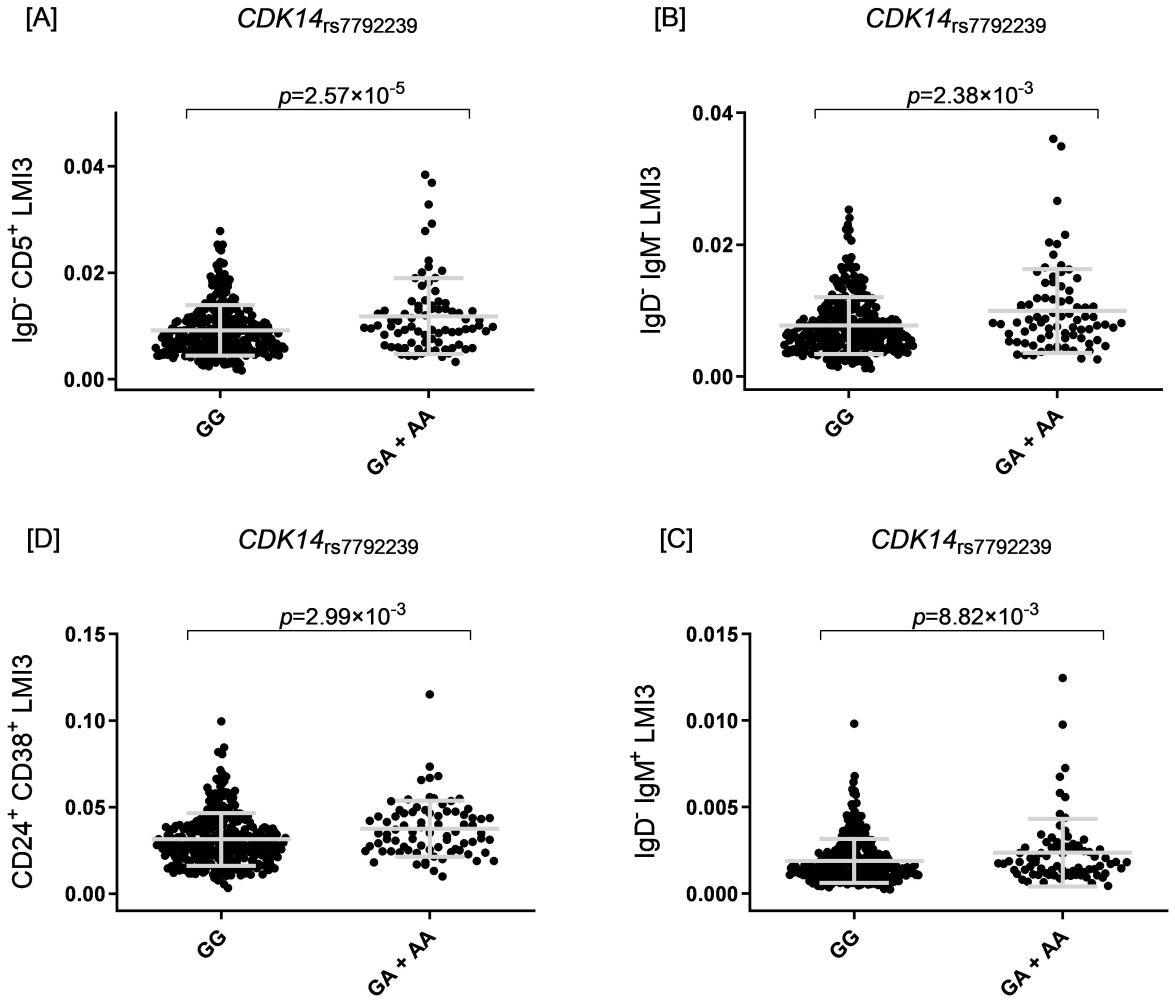
Four scatter plots labeled **(A–D)** show data for CDK14 rs7792239. [A] Plot for IgD^−^ CD5^+^ LMI3, comparing GG and GA + AA, with p = 2.57×10^−5^. [B] Plot for IgD^−^ IgM LMI3, comparing GG and GA + AA, with p = 2.38×10^−3^. [C] Plot for IgD^−^ IgM^+^ LMI3, comparing GG and GA + AA, with p = 8.82×10^−3^. [D] Plot for CD24^+^ CD38^+^ LMI3, comparing GG and GA + AA, with p = 2.99×10^−3^.

The two most statistically significant associations were *ENSG00000295231*|*ALDH1A2*_rs1350209880_ and *CYP26B1*_rs72845602_ (**Table 2**), which map to a LncRNA gene upstream *ALDH1A2* (Aldehyde Dehydrogenase 1 Family Member A2 gene, 15q21.3) and downstream of the CYP26B1 (Cytochrome P450 Family 26 Subfamily B Member 1 gene, 2p13.2, **Supplementary Figure 2A**). The other seven novel associations were for the *SLC6A11*|*LINC00606*_rs4088054_, *TMOD1*_rs117643807_, *CDK14*_rs7792239_, *SMASR|SMAD3-DT*_rs28485994_, *CCDC172|PNLIPRP3*_rs7907582_, *GRIN2A|LOC105371076*_rs34340658_ and *SNX24|LOC124901213*_rs55770715_ SNPs (**Table 2**, **Supplementary Figures 2B–H**). At functional level, we found, for the first time, a significant association of the *CDK14*_rs7792239A_ allele with increased absolute numbers of IgD^-^CD5^+^ immature memory B lymphocytes (*p*=2.57×10^-5^, **Figure 2A**) and potential associations with increased absolute numbers of other immature memory B lymphocytes, including IgD^-^IgM^-^, CD24^+^CD38^+^ and IgD^-^IgM^+^ (*p*=2.38×10^-3^; *p*=2.99×10^-3^ and *p*=8.82×10^-3^, respectively, **Figures 2B**, **4D**).

**Figure 3:**
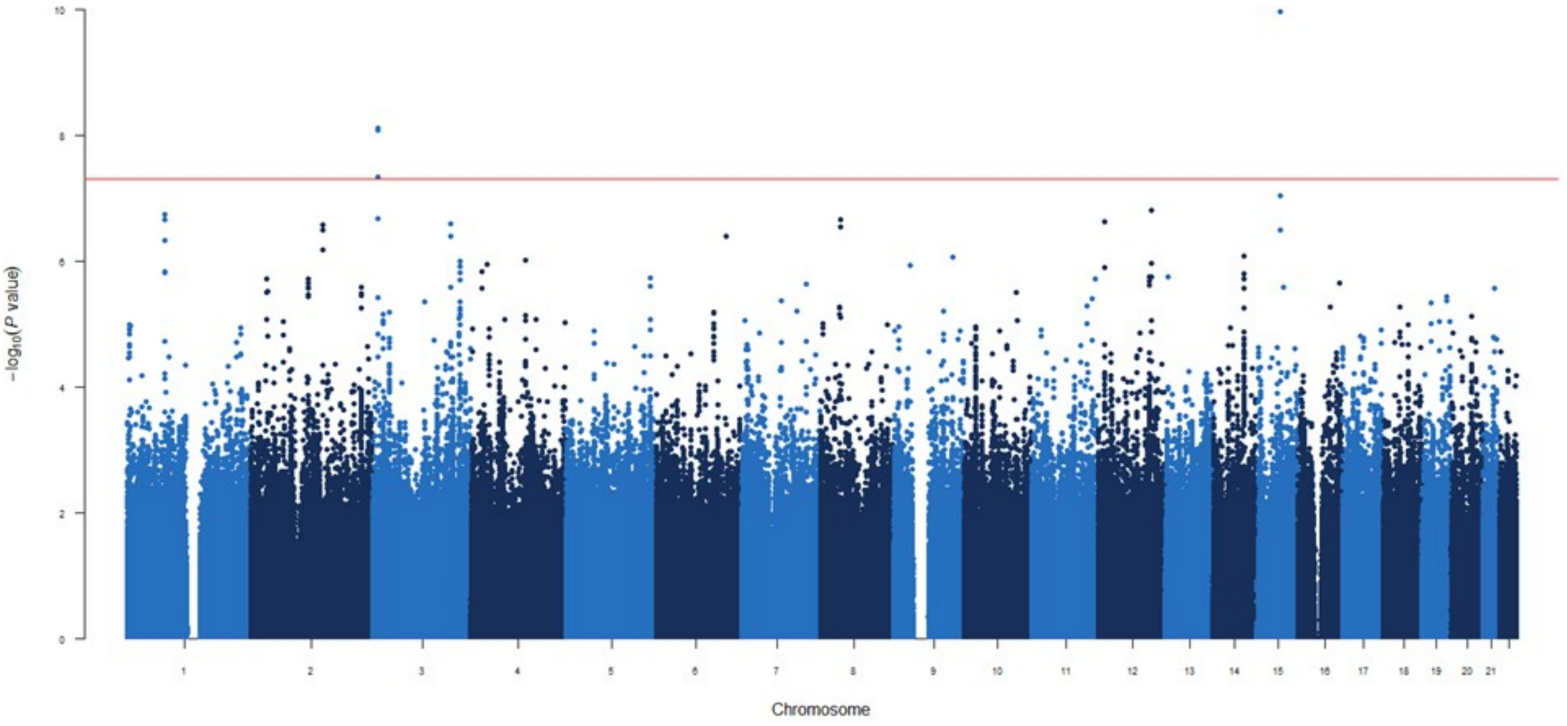
Manhattan plot for the GWAS analysis for IgG levels measured three months after mRNA-1273 vaccine.

**Figure 4:**
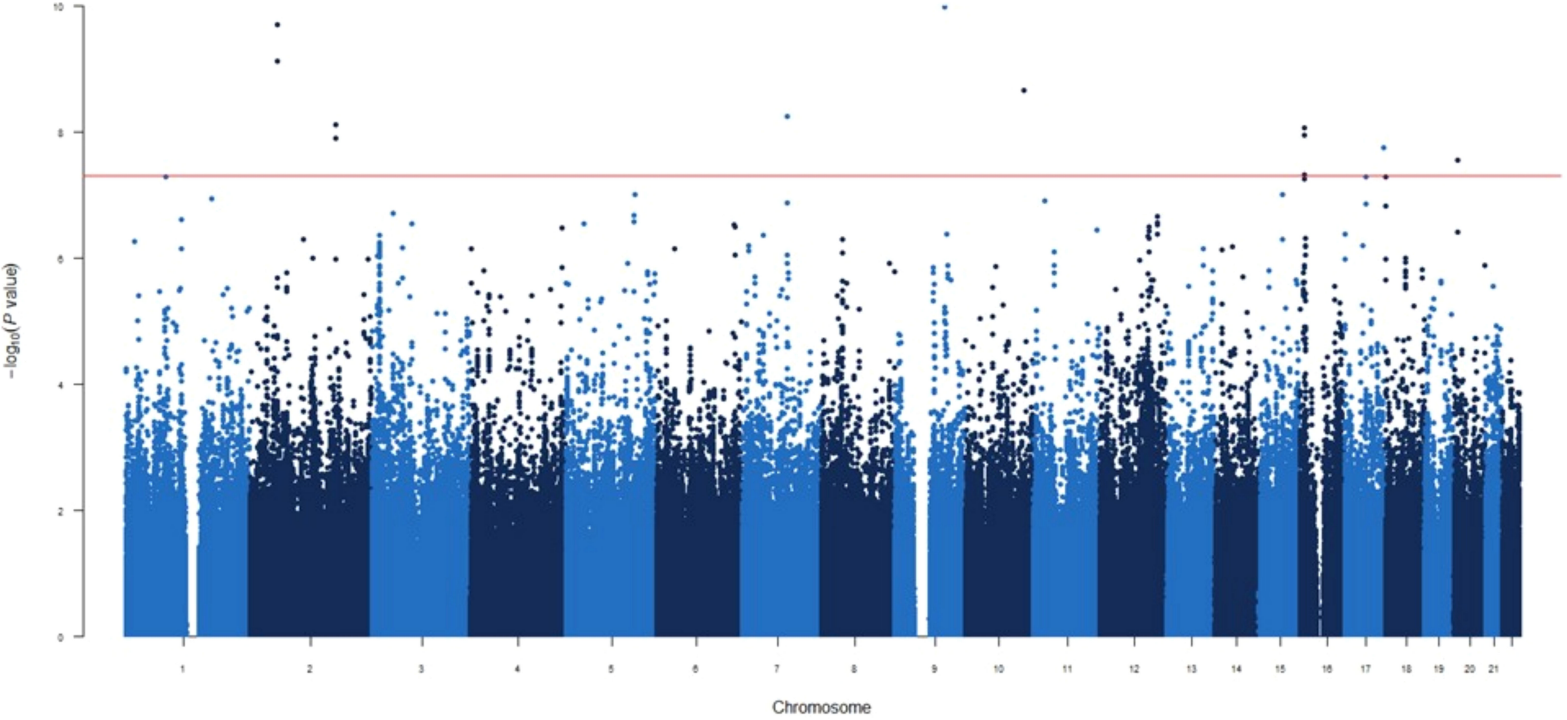
Manhattan plot for the GWAS analysis for difference IgG levels between one and three months after mRNA-1273 vaccine.

In addition, carriers of the *SNX24*|*LOC124901213*_rs55770715A_ allele had lower expression levels of *SNX24* mRNAs in different tissues (*p*=9.63×10^-6^-2.60×10^-4^, **Supplementary Table 4**, **Supplementary Figures 2C**, **5A, B**). Interestingly, we also identified 19 novel potentially interesting associations with increased circulating concentrations of IgG at 1-month post-vaccination, which remained borderline significant. These association signals were located near the *ENSG00000307057*, *TNFSF4*|*LOC100506023*, *THOC1*|*COLEC12*, *METTL8*, *UOX*, *ZNF516*, *PLCB1*, *SOCS3*, *ENSG00000232855*|*ENSG00000307297*, *CACNA1A*, *PLXNC1*, *ENSG00000234703*|*RUNX1*, *LYZL1*, *ENSG00000300202*, *LOC105369715*, *ENSG00000226566*|*ENSG00000307505*, *ENSG00000229618*, *PLAT* and *RNU6-1326P*|*USP25 loci* (*p*=7.76×10^-8^ – 5.41×10^-7^, **Supplementary Table 5**).

### GWAS at 3 months post-vaccination

In the GWAS at 3 months post-vaccination, two novel independent genetic signals were identified associated with increased circulating IgG levels (**Figure 3**). These two significant associations were the *SMASR|SMAD3-DT*_rs28485994_ and *ATP2B2*_rs55725269_ (OR=2.15, *p*=3.66×10^-12^ and OR=2.25, *p*=7.62×10^-9^, respectively, **Table 2**).

The first signal maps between two LncRNA genes on chromosome 15q21.3 while the second is located downstream of the ATPase Plasma Membrane Ca^2+^ Transporting 2 gene on chromosome 3p25.3 (**Supplementary Figure 3**). Interestingly, these signals were previously identified in the GWAS after 1 month vaccine, with the first being identified directly and the second associated with *SLC6A11*|*LINC00606*_rs4088054_, which shows modest LD (D’=1.00 and r^2^ = 0.584). Additionally, we identified nine novel potential associated signals with borderline significance in the *NUAK1*|*CKAP4*, *ST6GALNAC3*, *LOC105379385*, *GPRC5A*, *GTDC1*, *FILNC1*, *CEP128*, *LOC105376235* and *COL25A1 loci* associated with de increased levels of IgG after third month vaccine (*p*=1.58×10^-7^-9.58×10^-7^, **Supplementary Table 5**).

### GWAS for differential antibody responses at 1 vs 3 months post-vaccination

**Figure 5:**
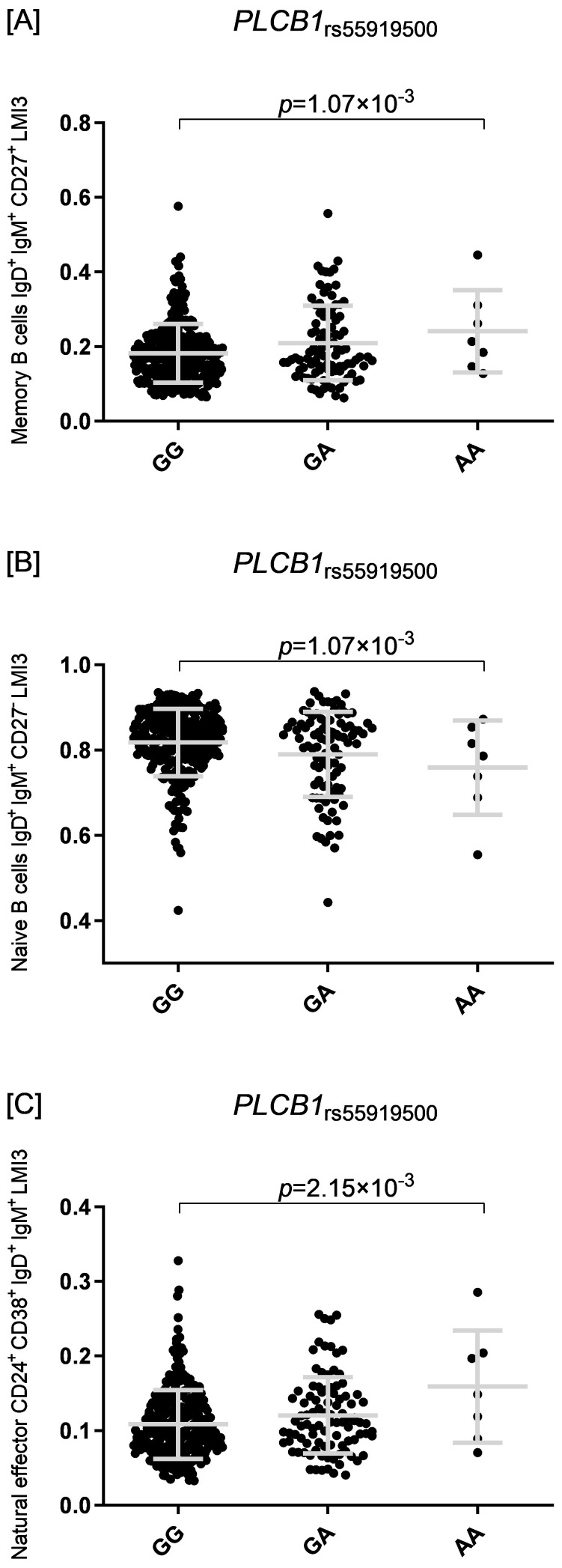
Three dot plots display B cell subsets associated with the PLCB1 rs55919500 genotype: GG, GA, and AA. Plot **(A)** shows memory B cells with a p-value of 1.07×10^−3^. Plot **(B)** presents naïve B cells with a similar p-value. Plot **(C)** illustrates natural effector B cells with a p-value of 2.15×10^−3^. Each plot includes individual data points and median lines.

In the third GWAS, conducted to evaluate the differences in circulating IgG levels between 1 and 3 months we identified nine novel genetic variants associated with the different IgG levels (**Figure 4**). Similar to the first GWAS, the most statistically significant association was the *ENSG00000295231*|*ALDH1A2*_rs1350209880_ SNP (OR=2.50, *p*=5.01×10^-13^, **Table 2**) which maps on LncRNA gene and upstream *ALDH1A2* gene on chromosome 15q21.3. The next five significant associations were *TMOD1*_rs117643807_, *SPTBN1*_rs75197984_, *CCDC172|PNLIPRP3*_rs7907582_, *CDK14*_rs7792239_ and *GRIN2A|LOC105371076*_rs34340658_ SNPs (OR=2.54, *p*=1.04×10^-10^; OR=2.37, *p*=1.97×10^-10^ OR=2.18, *p*=2.15×10^-09^; OR=2.39, *p*=5.65×10^-09^ and OR=1.95, *p*=8.64×10^-09^, respectively, **Table 2**, **Supplementary Table 5**) were also directly identified in the first GWAS, with the exception of *SPTBN1*_rs75197984_ which shows slight linkage disequilibrium (LD) with *CYP26B1*_rs72845602_ (D’=0.601 and r^2^ = 0.001). In addition, the other three significant associations were *METTL8*_rs1125991_, *LOC105371912*_rs4630616_ and *PLCB1*_rs55919500_ (OR=2.03, *p*=7.71×10^-09^; OR=1.87, *p*=1.78×10^-08^ and OR=2.24, *p*=2.81×10^-08^, respectively, **Table 2**, **Supplementary Table 5**) which were identified for the first time, in this GWAS. Besides the functional impact of the *CDK14* SNP on absolute numbers of different immature memory B lymphocytes mentioned previously, we found that carriers of the *PLCB1*_rs55919500A_ allele had increased absolute numbers of IgD^+^IgM^+^CD27^+^ memory B cells and natural effector CD24^+^CD38^+^IgD^+^IgM^+^ B cells (*p*=1.07×10^-3^ and *p*=2.15×10^-3^, respectively, **Figures 5A, B**). Intriguingly, carriers of the *PLCB1*_rs55919500A_ allele also had decreased absolute numbers of naïve IgD^+^IgM^+^CD27^-^ B cells (*p*=2.38×10^-3^, **Figure 5C**).

Finally, we identified 28 novel potential associated signals with borderline significance (5×10^-8^<p<1×10^-6^) in the *THOC1*|*COLEC12*, *ENSG00000301718*|*ENSG00000295563*, *SMASR*|*SMAD3-DT*, *ETF1*, *TNFSF4* | *LOC100506023*, *lnc-LUZP2-3*|*HSALNG0143151*, *RN7SKP216*|*ENSG00000258254*, *LOC105378072*|*LOC101928923*, *LOC101928923*, *ENSG00000258272*, *ENSG00000299420*, *ENSG00000307057*, *BAAT*, *ENSG00000294440*|*ENSG00000308043*, *BTD*, *HAL*, *ENSG00000309019*|*MYL9*, *LINC01918*|*ENSG00000293860*, *LOC105371757*, *ENSG00000300202*, *PTPRG*, *TMIGD3*, *PCGF3-AS1*, *GPC5*, *LOC105376642*|*HNRNPKP3*, *LINC00606*|*ENSG00000230599*, *HACL1* and *CNTNAP5 loci* associated with difference circulating levels of IgG between first and third month vaccine (*p*=5.10×10^-8^-9.87×10^-7^, **Supplementary Table 5**).

Of note, none of the genetic signals identified in these GWASs showed association with baseline IgG levels measured in the 500FG cohort of the HFGP, which confirms that the reported associations are specific to the immune response elicited by vaccination (**Supplementary Table 5**).

## Discussion

This comprehensive study identified, for the first time, 14 genetic variants significantly associated with increased circulating IgG levels at 1 month and 3 months post-vaccination with the mRNA-1273 vaccine or in the GWAS assessing differential antibody responses between 1- and 3-months post-vaccination.

The strongest association was for the *ENSG00000295231*|*ALDH1A2*_rs1350209880_ SNP, located within a LncRNA gene and upstream of *ALDH1A2* gene on chromosome 15q21.3. This variant showed the most significant association in the first GWAS, at 1-month post-vaccination, and in third GWAS to differences in circulating IgG levels between months 1 and 3 post-vaccination, suggesting a role in sustaining IgG production and possibly influencing the magnitude or persistence of the humoral immune response. While rs1350209880 is annotated as a rare variant in external databases, it exhibited a minor allele frequency above 0.05 in our cohort, likely reflecting population-specific enrichment. This justified its retention following standard GWAS quality control thresholds. Genotyping quality metrics were robust and supported the validity of this signal; however, we acknowledge that associations involving population-enriched or low-frequency variants should be interpreted with caution and warrant replication in independent cohorts.

Although *ENSG00000295231* LncRNA remains uncharacterized, several studies have shown that LncRNAs are important regulators in immune diseases (41–43), cancer (44–46) and various biological pathways (47, 48). *ALDH1A2* encodes Aldehyde Dehydrogenase 1 Family Member A2, which catalyzes the NAD-dependent oxidation of retinaldehyde to retinoic acid, a key signaling molecule involved in immune genes regulation (49–51). Retinoic acid, a metabolite of vitamin A, is essential for enhancing and sustaining IgG immune responses in B cell (52) and *in vivo* (53). It is also used as an adjuvant to boost mucosal/systemic immune responses and cytokine production (52, 54, 55). Specifically, retinoic acid may help reduce respiratory complications and aid epithelial repair after SARS-CoV-2 infection, due to its immune-modulating and anti-inflammatory properties (56, 57).

Supporting this role, we found *CYP26B1*_rs72845602_ SNP significantly associated with higher concentrations of circulating IgG levels 1-month post-vaccination. This SNP lies within *CYP26B1* gene, which encodes a cytochrome P450 enzyme that metabolizes all-trans retinoic acid and influences T cell differentiation and inflammation (54, 58–60). Another SNP, *SPTBN1*_rs75197984_, in moderate LD with *CYP26B1*_rs72845602_, was associated with increased circulating concentrations of IgG at 3 months post-vaccination. This genetic variant maps to Spectrin Beta, Non-Erythrocytic 1 (SPTBN1), encoding βII-spectrin, a cytoskeletal protein involved in cell shape, membrane organization, and protein sorting (61–63). Though not directly linked to circulating IgG levels or SARS-CoV-2, *SPTBN1* is involved in modulating immune regulating and viral infections, including HIV-1 (64–66). *In silico* analyses showed both SNPs are associated with several QTLs in blood, CD4^+^ naïve T cells, and CD14^+^ monocytes, and alter regulatory motifs in Gfi1, a transcription factor essential for B cell differentiation and IgG class switching (67–69). Gfi1-deficient B cells produce more IgG2a and IgG2b, likely via increased TGF-β1 expression, which regulates IgG subclass production (69). These findings suggest that *CYP26B1*_rs72845602_ and *SPTBN1*_rs75197984_ may influence IgG production by modulating immune cell function and response to vaccination.

Likewise, *SLC6A11*|*LINC00606*_rs4088054_ maps to a validated LncRNA and the Solute Carrier Family 6 Member 11 (SLC6A11) genes, which encodes a sodium-dependent gamma-aminobutyric acid (GABA) transporter. GABA, an inhibitory neurotransmitter, is involved in modulation of immune cell activation via its transporters in T cells and macrophages (70). At 3 months post-vaccination, *ATP2B2*_rs55725269_ SNP, in complete LD with *SLC6A11*|*LINC00606*_rs4088054_, was also associated with elevated circulating levels of IgG. This SNP lies downstream of the ATPase Plasma Membrane Ca2+ Transporting 2 (ATP2B2), encoding PMCA2, a plasma membrane calcium ATPase critical for restoring calcium balance in T cells (71, 72) thus regulating immune response intensity and duration (73). These results suggest that both SNPs may elevate IgG concentrations through immune modulation, possibly via calcium-dependent effects of ATP2B2 on SLC6A11 function. Supporting this, a recent study associated genetic variants within *ATP2B2* with higher mortality in severe SARS-CoV-2 cases (74), while GWAS have associated the *SLC6A11* locus with gut microbiome composition (75, 76). Given the microbiome’s role in SARS-CoV-2 infection (77–79), and vaccine efficacy (80, 81), *SLC6A11* may impact circulating IgG concentrations via microbiome modulation.

Another noteworthy finding from the 1-month post-vaccination GWAS was the association of *SNX24|LOC124901213*_rs55770715_ with higher circulating concentrations of IgG. This SNP maps *SNX24* and a small nucleolar RNA gene at 5q23.2. SNX24, a member of the Sorting Nexin family, is involved in regulating protein trafficking through the endocytic pathway (82). Variants in *SNX24* have been linked to platelet indices (83, 84), and upregulation in megakaryocytes with ploidy (85). Our data and the finding that *SNX24* is downregulated in FLI1-deficient platelets support a role in platelet formation. *SNX24* is also required for α-granule biogenesis and cargo trafficking in megakaryocytes (86). This SNP overlaps histone marks in blood and lung cells, suggesting it may act as a regulatory element. It is also associated with *SNX24* expression in lung and spleen and constitutes an mQTL in blood, indicating a potential regulatory role in immune response gene expression.

In addition to *ENSG00000295231*|*ALDH1A2*_rs1350209880_ five other SNPs were associated with antibody responses from 1 to 3 months post-vaccination (*CDK14*_rs7792239_, *CCDC172*|*PNLIPRP3*_rs7907582_, *TMOD1*_rs117643807_, *GRIN2A*|*LOC105371076*_rs34340658_, and *LOC105371912*_rs4630616_), indicating their likely involvement in regulating antibody production over time.

The *CDK14*_rs7792239_ SNP maps to Cyclin Dependent Kinase 14 (CDK14) gene, which regulates the G2/M cell cycle and supports endothelial and epithelial proliferation and migration (87, 88). A recent mouse study linked CDK14 with interferon-gamma (IFN-γ) pathways, suggesting a key role in lung immune repair (89). The *CDK14*_rs7792239A_ allele was associated with increased absolute numbers of IgD^-^CD5^+^ immature memory B lymphocytes, and trends (though not significant) towards increases in absolute numbers of IgD^-^IgM^-^, CD24^+^CD38^+^ and IgD^-^IgM^+^ immature memory B cells. Previous studies have reported shifts in B cell memory populations, especially increased IgG^+^ memory B cells, after SARS-CoV-2 infection and vaccination (90–92). Given CDK14’s link to IFN-γ/STAT1 signaling and its potential to promote IgG production, this variant may support humoral responses. Notably, *CDK14*_rs7792239_ affects the E2A_2 regulatory motif, which is crucial for B cell development, germinal center formation, and IgG production (93–95). CDK14 thus appears vital for effective humoral immunity and protection against SARS-CoV-2.

The *CCDC172*|*PNLIPRP3*_rs7907582_ SNP is located between the *CCDC172* and *PNLIPRP3* genes on chromosome 10q25.3. *CCDC172* participates in protein-protein interactions and structural integrity, while *PNLIPRP3* encodes for a pancreatic lipase-like protein. Though their roles in SARS-CoV-2 are unclear, *in silico* analyses catalogued the SNP as probably malignant, with effects on regulatory motifs like CEBPB_known4 and Maf_known3/4, which showed altered activity in monocytes from hospitalized SARS-Cov-2 patients (96) and are activated in respiratory epithelial cells during severe infection (97). This suggests a potential role of CEBPB in modulating immune responses in the context of severe viral infection. Additionally, its paralog, CEBPD, has been implicated in driving immune cell responses in monocytes through IL-6-associated survival pathways (98). In support of this hypothesis, it has been found that the Maf_known3/4 motif is a transcription factor MAFB family member, which shows survival-associated upregulation in monocytes (98).

On the other hand, the *TMOD1*_rs117643807_ SNP maps to the Tropomoduling 1 (*TMOD1*) gene, which encodes an acting-capping protein that regulates cytoskeleton dynamics and is involved in cell shape, motility and signaling. *TMOD1* is essential in erythroid and cardiac cells, but recent studies suggest it also plays a role in immune regulation by maintaining cytoskeletal structure during immune cell activation and trafficking (99). In dendritic cells, it is also critical for proper maturation and function, reducing the ability of these cells to stimulate T cells and shifts cytokine secretion toward immune tolerance (100). While not directly linked to IgG levels or SARS-CoV-2, this variant could influence B cell activation and function via cytoskeletal remodeling and modulation of phagocyte’s activities.

The *GRIN2A*|*LOC105371076*_rs34340658_ SNP is located between *GRIN2A* gene, which encodes a subunit of the N-methyl-D-aspartate (NMDA) receptor involved in synaptic signaling, and a LncRNA at 16p13.2. While *GRIN2A* has mainly been studied in neurological disorders, growing evidence indicates cross-talk between neurotransmission and host immunity (101–103). Some studies have shown that non-neutralizing anti-SARS-CoV-2 IgG antibodies, particularly those against the S protein, can alter central nervous system (CNS) gene expression in mice, including upregulation of *GRIN2A* in the hippocampus. This suggests that such antibodies may influence neuronal activity and contribute to neurological symptoms seen in SARS-CoV-2 patients and vaccinated individuals. Given *GRIN2A*’s role in synaptic plasticity, preventing the production of anti-S1-111 IgG or similar antibodies could help reduce CNS manifestations of SARS-CoV-2 infection and vaccination (104). Interestingly, these observations are consistent with *in silico* findings linking this SNP—and similarly, the marker identified in *CKD14*—to altered E2A_2 and E2A_5 regulatory motifs. These motifs are bound by E2A and, as mentioned above, are involved in modulating B cell development, germinal center formation, and IgG production (93–95). Therefore, *GRIN2A*|*LOC105371076*_rs34340658_ likely contributes to effective humoral responses and protection against SARS-CoV-2.

Lastly, the *LOC105371912*_rs4630616_ SNP maps an uncharacterized ncRNA gene. Although its specific role in SARS-Cov-2 remains unknown, growing evidence suggests that lncRNAs are key regulatory elements in diverse biological processes, including the modulation and effectiveness of the immune responses during infection and following vaccination. Nonetheless, further studies are warranted to explore the potential involvement of this gene in SARS-CoV-2 pathogenesis.

At this point, it is also important to note that we found an association of the *SMASR|SMAD3-DT*_rs28485994_ SNP with increased circulating IgG concentrations in the GWAS conducted at 1 and 3 months post-vaccination, suggesting a role of this marker in sustaining antibody production over time. This SNP maps to *SMASR* and *SMAD3-DT*, lncRNA genes associated with the *SMAD3* gene. *SMAD3* encodes a key protein that cooperates with FOSL2 in the TGF-β signaling pathway, which suppresses type I IFN responses and promotes immune evasion (105–107). Recent studies have revealed a role for SMAD3 in SARS-CoV-2 pathogenesis. The SARS-CoV-2 N protein binds to SMAD3, enhancing TGF-β/SMAD3 signaling, which leads to G1 cell cycle arrest and tubular epithelial cell death via necroptosis (108–110). SMAD3 also downregulates Cystic Fibrosis Transmembrane Conductance Regulator (CFTR), increasing intracellular chloride levels and triggering the release of pro-inflammatory cytokines. In line with this, some studies have shown that SMAD3 suppresses miR-145, exacerbating CFTR dysfunction and inflammation (111), and that in severe SARS-CoV-2, there is an increase in polymorphonuclear myeloid-derived suppressor cells (PMN-MDSCs) that suppress T-cell activity through reactive oxygen species (ROS) (112). These findings suggest that the role of TGF-β/SMAD3 in immune regulation could potentially influence B-cell responses, particularly by modulating IgG levels after vaccination.

Finally, another noteworthy finding of this study was the identification of three SNPs—*PLCB1*_rs55919500_, *METTL8*_rs1125991_, and *LOC105371912*_rs4630616_—associated with differential antibody responses. These variants point toward potentially important molecular mechanisms that link genetic regulation with immune cell function, particularly in the context of viral infections such as SARS-CoV-2. *PLCB1* encodes phospholipase C beta 1, an enzyme involved in intracellular signaling pathways that regulate inflammation and immune responses through modulation of proinflammatory cytokines such as IL-1β, IL-6, and IL-8 (113, 114). Consistent with this function, *in vitro* analysis showed that carriers of the *PLCB1*_rs55919500A_ allele had an increased absolute number of memory B cells (IgD^+^IgM^+^CD27^+^) and natural effector B cells (CD24^+^CD38^+^IgD^+^IgM^+^), as well as fewer naïve B cells (IgD^+^IgM^+^CD27^-^). These subsets have been implicated in the response to SARS-CoV-2 infection and vaccination (115, 116), with unswitched memory B cells (IgD^+^IgM^+^CD27^+^) found to be increased in individuals recovering from severe SARS-CoV-2 compared to those with milder disease (115). *METTL8*, on the other hand, encodes a mitochondrial RNA methyltransferase responsible for installing 3-methylcytidine (m3C) modifications in specific mitochondrial tRNAs, a process essential for efficient mitochondrial translation and respiratory chain activity (117, 118). RNA methylation, including by METTL family enzymes, has been recognized as a key regulator of immune cell function, differentiation, and tumor immune evasion (119, 120). In addition, *METTL8*_rs1125991_ was associated with changes in the regulatory motif of FOXO1, a transcription factor crucial for B cell development, tolerance, and function. Supporting a functional role, this SNP was strongly linked to increased expression of CYBRD1 mRNA in blood and was identified as an mQTL in both blood and naïve CD4^+^ T cells. CYBRD1 encodes an iron-regulated ferric reductase, and altered expression may modulate immune responses by affecting iron metabolism, ferroptosis (iron-dependent cell death), and the tumor microenvironment (121). Notably, components of mitochondrial cytochrome systems have been found to be elevated in the plasma of SARS-CoV-2 patients, reflecting mitochondrial dysfunction and apoptosis during severe infection (122). Moreover, METTL8 itself has been implicated in CD8^+^ T cell infiltration in lung squamous cell carcinoma, suggesting a broader role in regulating immune responses within tissue microenvironments (123). Taken together, these findings suggest that genetic variation in *PLCB1* and *METTL8* influences immune cell profiles and functions through distinct molecular mechanisms, namely, inflammatory signaling, mitochondrial RNA modification, and transcriptional regulation. These mechanisms converge to shape the host’s antibody responses and may modulate the outcome of SARS-CoV-2.

In summary, this study identified fourteen genetic variants linked to increased circulating IgG levels following mRNA-1273 vaccination, with the most notable being *ENSG00000295231|ALDH1A2*_rs1350209880_, a variant associated with immune regulation and enhanced IgG production over time. Other key variants mapped to genes like *CYP26B1*, *SPTBN1*, and *SMASR|SMAD3-DT*, which influences cytokine production, T cell differentiation, and IgG subclass production, were crucial for optimizing immune responses. Variants in *CDK14* and *GRIN2A* were directly linked to B cell activation and memory, further shaping the humoral immune response. Despite other variants map on LncRNAs that may regulate IgG production through mechanisms like neurotransmission and calcium signaling and need further investigation. Finally, variants including *TMOD1*_rs117643807_ and *PLCB1*_rs55919500_, suggest immune modulation via cell signaling pathways, while variants like *ATP2B2*_rs55725269_ and *METTL8*_rs1125991_ highlight the role of metabolism and the microbiome in vaccine responses.

Although this study provides valuable insights into the genetic predisposition to increase IgG levels after vaccination, it also has several limitations. First, our analysis was restricted to individuals of the Spanish cohort, which limited our ability to replicate previous findings from studies in other ethnic populations. This decision was based on both scientific and logistical considerations. Focusing on a genetically homogeneous population increases internal consistency and reduces confounding due to population structure and environmental heterogeneity, thereby enhancing the robustness of GWAS findings. Additionally, we did not have access to large-scale, individual-level data from other vaccinated cohorts, which limited the feasibility of multi-cohort or trans-ethnic analyses. While we do not assume fundamental differences in immune function between European populations, replication in independent cohorts will be essential to validate and generalize our results.

Second, we were unable to confirm the association of several potentially relevant *loci*. This could be attributed to the relatively limited statistical power of our study. Differences in the vaccine platform used (e.g., mRNA-1273 in our study versus BNT162b2 or mixed vaccine platforms in other studies), distinct timepoints for sample collection, population-specific allele frequencies, and varying analytical strategies may also explain these discrepancies. For instance, recent large-scale GWAS such as the UK Biobank study (124) analyzed combined data from different vaccine types without distinguishing between platforms like BNT162b2 and ChAdOx1, while our study focused specifically on the mRNA-1273 vaccine, providing greater homogeneity in immune stimulus and timing. Additionally, some of the previously identified variants may have modest effect sizes that require larger sample sizes or meta-analyses to achieve statistical significance. The absence of overlapping associations therefore does not rule out the relevance of these loci but underscores the complexity of genetic regulation of vaccine responses and the importance of harmonized designs across studies for robust cross-cohort validation. Finally, while our functional and *in silico* analyses offer an initial understanding of the potential effects of GWAS hits, further experimental validation is needed to definitively establish the biological roles of these markers. Additionally, while our functional and *in silico* analyses offer an initial understanding of the potential effects of GWAS hits, further experimental validation is needed to definitively establish the biological roles of these markers.

Third, although age and sex are known factors influencing immune responses, we did not perform stratified analyses by sex or age strata due to limited statistical power, which would reduce the reliability of such subgroup analyses. Notably, previous studies have consistently reported that vaccine-induced antibody titers tend to be statistically higher in women than in men, highlighting sex as an important biological variable in immune responses (125, 126). For example, Demombreun et al. demonstrated higher SARS-CoV-2 antibody titers post-vaccination in women compared to men (125), while Jensen et al. also reported sex differences favoring stronger humoral responses in females (126). However, our findings did not replicate this trend, as shown in **Supplementary Table 3**, where no statistically significant difference or even a trend toward higher titers in men was observed. These discrepancies may be explained by several factors. Our cohort consisted exclusively of individuals of European ancestry, whereas Demombreun et al. included a more diverse racial and ethnic population including Hispanic/Latinx, Non-Hispanic Asian, Non-Hispanic Black, among others. Moreover, women in our study were slightly older and represented a larger sample size than men, which could influence antibody levels. In addition, differences in antibody measurements may contribute to the contrasting results. Our study measured anti-Spike IgG antibodies, reflecting a broader antibody response, whereas the cited studies focused on anti-RBD IgG antibodies, which target a specific region of the spike protein. Although related, these assays are not directly comparable. On the other hand, the referenced studies assessed immune responses at baseline, after the first dose, and after the second dose, while our measurements were performed at one and three months post full vaccination, capturing a later phase of the immune response, which may display different sex-related patterns. Furthermore, biological and immunological factors likely contribute to these differences. It is well established that innate and adaptive immune responses tend to be stronger and faster in women, which can lead to higher antibody titers but also increased susceptibility to autoimmune diseases and more frequent vaccine adverse reactions (127). However, some studies have demonstrated no evidence of stronger vaccine-induced immunity in females compared to males (128), suggesting that sex differences may vary by context and methodology. Hormonal differences, such as higher estrogen levels in women and testosterone in men, modulate immune responses and may partially explain sex differences in vaccine efficacy and reactogenicity. Age also interacts with sex in shaping vaccine responses; while some vaccines are more effective in younger women (127), sex differences in adverse reactions may persist regardless of age.

Considering all the above, age and sex were included as covariates in all GWAS models, helping to control for their potential confounding effects. Future studies with larger cohorts are needed to rigorously assess the impact of age and sex on immune outcomes and genetic associations.

Finally, it is important to note that our study design included antibody measurements at only two post-vaccination time points, 1 and 3 months, which limits the ability to fully characterize the longitudinal dynamics of the IgG response. While our GWAS comparing differences between these time points partially captures interindividual variability in antibody kinetics, the use of static snapshots may overlook important temporal patterns such as early peaks, delayed responses, or rapid waning. Future studies incorporating denser longitudinal sampling, particularly in the early and late phases post-vaccination, will be essential to better understand the genetic regulation of humoral values over time.

These findings underscore the complex interplay of genetic factors influencing the immune response to vaccination, particularly through modulation of B cell activity, immune signaling pathways, and metabolic processes. These insights could inform future strategies to enhance vaccine efficacy, especially for SARS-CoV-2 and other viral infections. The identification of genetic variants provides a novel and deeper understanding of individual variability in vaccine responses and opens new avenues for personalized vaccine strategies.

## Data Availability

The datasets presented in this study can be found in online repositories. The names of the repository/repositories and accession number(s) can be found below: https://hfgp.bbmri.nl/, BBMRI-NL data infrastructure ftp.genyo.es, GENYO repository.

## References

[1] Iyanda AE Adeleke R Lu Y Osayomi T Adaralegbe A Lasode M. A retrospective cross-national examination of COVID-19 outbreak in 175 countries: a multiscale geographically weighted regression analysis (January 11-June 28, 2020). J Infect Public Health. (2020) 13:1438–45. doi: 10.1016/j.jiph.2020.07.006, PMID: 32773211 PMC7375316

[2] WHO. Coronavirus (COVID-19) dashboard. Available online at: https://covid19.who.int/ (Accessed August 14, 2024).

[3] Contini C Caselli E Martini F Maritati M Torreggiani E Seraceni S. COVID-19 is a multifaceted challenging pandemic which needs urgent public health interventions. Microorganisms. (2020) 8:1228. doi: 10.3390/microorganisms8081228, PMID: 32806657 PMC7464234

[4] Maiti BK. Potential role of peptide-based antiviral therapy against SARS-CoV-2 infection. ACS Pharmacol Transl Sci. (2020) 3:783–5. doi: 10.1021/acsptsci.0c00081, PMID: 32821885 PMC7393772

[5] Bui LT Winters NI Chung MI Joseph C Gutierrez AJ Habermann AC. Chronic lung diseases are associated with gene expression programs favoring SARS-CoV-2 entry and severity. Nat Commun. (2021) 12:4314. doi: 10.1038/s41467-021-24467-0, PMID: 34262047 PMC8280215

[6] Xia H Cao Z Xie X Zhang X Chen JY-C Wang H. Evasion of type I interferon by SARS-CoV-2. Cell Rep. (2020) 33. doi: 10.1016/j.celrep.2020.108234, PMID: 32979938 PMC7501843

[7] Hao Y-J Wang Y-L Wang M-Y Zhou L Shi J-Y Cao J-M. The origins of COVID-19 pandemic: A brief overview. Transbound Emerg Dis. (2022) 69:3181–97. doi: 10.1111/tbed.14732, PMID: 36218169 PMC9874793

[8] Yang R Deng Y Huang B Huang L Lin A Li Y. A core-shell structured COVID-19 mRNA vaccine with favorable biodistribution pattern and promising immunity. Signal Transduct Target Ther. (2021) 6:213. doi: 10.1038/s41392-021-00634-z, PMID: 34059617 PMC8165147

[9] Verheul MK Nijhof KH de Zeeuw-Brouwer MI Duijm G ten Hulscher H de Rond L. Booster immunization improves memory B cell responses in older adults unresponsive to primary SARS-CoV-2 immunization. Vaccines (Basel). (2023) 11:1196. doi: 10.3390/vaccines11071196, PMID: 37515012 PMC10384172

[10] Krauson AJ Casimero FVC Siddiquee Z Stone JR. Duration of SARS-CoV-2 mRNA vaccine persistence and factors associated with cardiac involvement in recently vaccinated patients. NPJ Vaccines. (2023) 8:141. doi: 10.1038/s41541-023-00742-7, PMID: 37758751 PMC10533894

[11] Anderson EJ Rouphael NG Widge AT Jackson LA Roberts PC Makhene M. Safety and immunogenicity of SARS-CoV-2 mRNA-1273 vaccine in older adults. New Engl J Med. (2020) 383:2427–38. doi: 10.1056/NEJMoa2028436, PMID: 32991794 PMC7556339

[12] Zhang H Xu N Xu Y Qin P Dai R Xu B. Safety and immunogenicity of Ad5-nCoV immunization after three-dose priming with inactivated SARS-CoV-2 vaccine in Chinese adults. Nat Commun. (2023) 14:4757. doi: 10.1038/s41467-023-40489-2, PMID: 37553338 PMC10409730

[13] Gutiérrez-Bautista JF Sampedro A Gómez-Vicente E Rodríguez-Granger J Reguera JA Cobo F. HLA class II polymorphism and humoral immunity induced by the SARS-CoV-2 mRNA-1273 vaccine. Vaccines (Basel). (2022) 10:402. doi: 10.3390/vaccines10030402, PMID: 35335034 PMC8949280

[14] Esposito M Minnai F Copetti M Miscio G Perna R Piepoli A. Human leukocyte antigen variants associate with BNT162b2 mRNA vaccine response. Commun Med. (2024) 4:63. doi: 10.1038/s43856-024-00490-2, PMID: 38575714 PMC10995155

[15] Magri C Marchina E Sansone E D'Adamo AP Cappellani S Bonfanti C. Genome-wide association studies of response and side effects to the BNT162b2 vaccine in Italian healthcare workers: Increased antibody levels and side effects in carriers of the HLA-A* 03: 01 allele. HLA. (2023) 102:707–19. doi: 10.1111/tan.15157, PMID: 37469131

[16] Li P Shi D Shen W Shi S Guo X Li J. Pilot genome-wide association study of antibody response to inactivated SARS-CoV-2 vaccines. Front Immunol. (2022) 13:1054147. doi: 10.3389/fimmu.2022.1054147, PMID: 36451823 PMC9704361

[17] Bian S Guo X Yang X Francis SS Shu Y Liu S. Genetic determinants of IgG antibody response to COVID-19 vaccination. Am J Hum Genet. (2024) 111:181–99. doi: 10.1016/j.ajhg.2023.12.005, PMID: 38181733 PMC10806743

[18] Cruz R Diz-de Almeida S López de Heredia M Quintela I Ceballos FC Pita G. Novel genes and sex differences in COVID-19 severity. Hum Mol Genet. (2022) 31:3789–806. doi: 10.1093/hmg/ddac132, PMID: 35708486 PMC9652109

[19] The COVID-19 Host Genetics Initiative. A second update on mapping the human genetic architecture of COVID-19. Nature. (2023) 621:E7–E26. doi: 10.1038/s41586-023-06355-3, PMID: 37674002 PMC10482689

[20] Pairo-Castineira E Rawlik K Bretherick AD Qi T Wu Y Nassiri I. GWAS and meta-analysis identifies 49 genetic variants underlying critical COVID-19. Nature. (2023) 617:764–8. doi: 10.1038/s41586-023-06034-3, PMID: 37198478 PMC10208981

[21] Fukushima K Kubo T Ito Y Oda Y Nagayoshi Y Fukuda M. Humoral and cellular immune responses to mRNA COVID-19 vaccinations in the elderly: A longitudinal study in Japan. J Infect Chemother. (2025) 31. doi: 10.1016/j.jiac.2025.102695, PMID: 40189203

[22] Akhtar M Islam MR Khaton F Rahman F Sami TA Tauheed I. Spike specific IgG3 and nucleocapsid IgG response in serum serve as distinguishing immunological markers between SARS-CoV-2 infection and vaccination. Front Immunol. (2025) 16. doi: 10.3389/fimmu.2025.1518915, PMID: 40213555 PMC11983542

[23] Noé A Dang TD Axelrad C Burrell E Germano S Elia S. BNT162b2 COVID-19 vaccination in children alters cytokine responses to heterologous pathogens and Toll-like receptor agonists. Front Immunol. (2023) 14:1242380. doi: 10.3389/fimmu.2023.1242380, PMID: 37691937 PMC10485613

[24] The 1000 Genomes Project Consortium. A map of human genome variation from population-scale sequencing. Nature. (2010) 467:1061–73. doi: 10.1038/nature09534, PMID: 20981092 PMC3042601

[25] The Haplotype Reference Consortium. A reference panel of 64,976 haplotypes for genotype imputation. Nat Genet. (2016) 48:1279–83. doi: 10.1038/ng.3643, PMID: 27548312 PMC5388176

[26] Li Y Oosting M Smeekens SP Jaeger M Aguirre-Gamboa R Le KTT. A functional genomics approach to understand variation in cytokine production in humans. Cell. (2016) 167:1099–110.e14. doi: 10.1016/j.cell.2016.10.017, PMID: 27814507

[27] ter Horst R Jaeger M Smeekens SP Oosting M Swertz MA Li Y. Host and environmental factors influencing individual human cytokine responses. Cell. (2016) 167:1111–24.e13. doi: 10.1016/j.cell.2016.10.018, PMID: 27814508 PMC5787854

[28] Aguirre-Gamboa R Joosten I Urbano PCM van der Molen RG van Rijssen E van Cranenbroek B. Differential effects of environmental and genetic factors on T and B cell immune traits. Cell Rep. (2016) 17:2474–87. doi: 10.1016/j.celrep.2016.10.053, PMID: 27818087 PMC5130901

[29] Orrù V Steri M Sole G Sidore C Virdis F Dei M. Genetic variants regulating immune cell levels in health and disease. Cell. (2013) 155:242. doi: 10.1016/j.cell.2013.08.041, PMID: 24074872 PMC5541764

[30] Van Der Velde KJ Imhann F Charbon B Pang C van Enckevort D Slofstra M. MOLGENIS research: Advanced bioinformatics data software for non-bioinformaticians. Bioinformatics. (2019) 35:1076–8. doi: 10.1093/bioinformatics/bty742, PMID: 30165396 PMC6419911

[31] Wilkinson MD Dumontier M Aalbersberg IJ Appleton G Axton M Baak A. Comment: The FAIR Guiding Principles for scientific data management and stewardship. Sci Data. (2016) 3:1–9. doi: 10.1038/sdata.2016.18, PMID: 26978244 PMC4792175

[32] Watanabe K Taskesen E Van Bochoven A Posthuma D. Functional mapping and annotation of genetic associations with FUMA. Nat Commun. (2017) 8. doi: 10.1038/s41467-017-01261-5, PMID: 29184056 PMC5705698

[33] Ochoa D Hercules A Carmona M Suveges D Baker J Malangone C. The next-generation Open Targets Platform: reimagined, redesigned, rebuilt. Nucleic Acids Res. (2023) 51:D1353–9. doi: 10.1093/nar/gkac1046, PMID: 36399499 PMC9825572

[34] Lonsdale J Thomas J Salvatore M Phillips R Lo E Shad S. The genotype-tissue expression (GTEx) project. Nat Genet. (2013) 45:580–5. doi: 10.1038/ng.2653, PMID: 23715323 PMC4010069

[35] Zheng Z Huang D Wang J Zhao K Zhou Y Guo Z. QTLbase: an integrative resource for quantitative trait loci across multiple human molecular phenotypes. Nucleic Acids Res. (2020) 48:D983–91. doi: 10.1093/nar/gkz888, PMID: 31598699 PMC6943073

[36] Rentzsch P Witten D Cooper GM Shendure J Kircher M. CADD: predicting the deleteriousness of variants throughout the human genome. Nucleic Acids Res. (2019) 47:D886–94. doi: 10.1093/nar/gky1016, PMID: 30371827 PMC6323892

[37] Boyle AP Hong EL Hariharan M Chen Y Schaub MA Kasowski M. Annotation of functional variation in personal genomes using RegulomeDB. Genome Res. (2012) 22:1790–7. doi: 10.1101/gr.137323.112, PMID: 22955989 PMC3431494

[38] Van Der Most PJ Küpers LK Snieder H Nolte I. QCEWAS: automated quality control of results of epigenome-wide association studies. Bioinformatics. (2017) 33:1243–5. doi: 10.1093/bioinformatics/btw766, PMID: 28119308

[39] Wickham H. ggplot2. Wiley Interdiscip Rev Comput Stat. (2011) 3:180–5. doi: 10.1002/wics.147

[40] Turner SD. qqman: an R package for visualizing GWAS results using Q-Q and manhattan plots. J Open Source Softw. (2018) 3:731. doi: 10.21105/joss.00731

[41] Taheri M Noroozi R Sadeghpour S Omrani MD Ghafouri-Fard S. The rs4759314 SNP within Hotair lncRNA is associated with risk of multiple sclerosis. Mult Scler Relat Disord. (2020) 40:101986. doi: 10.1016/j.msard.2020.101986, PMID: 32058948

[42] Zhang R-X Zhang Z-X Zhao X-Y Liu Y-H Zhang X-M Han Q. Mechanism of action of lncRNA-NEAT1 in immune diseases. Front Genet. (2025) 16. doi: 10.3389/fgene.2025.1501115, PMID: 40110044 PMC11919857

[43] Seyedi D Espandar N Hojatizadeh M Mohammadi Y Sadri F Rezaei Z. Noncoding RNAs in rheumatoid arthritis: modulators of the NF-κB signaling pathway and therapeutic implications. Front Immunol. (2024) 15. doi: 10.3389/fimmu.2024.1486476, PMID: 39530095 PMC11550995

[44] Huang X Zhang W Shao Z. Association between long non-coding RNA polymorphisms and cancer risk: a meta-analysis. Biosci Rep. (2018) 38:BSR20180365. doi: 10.1042/BSR20180365, PMID: 29802154 PMC6066654

[45] Mu D Shi Y Sun R Han B Zhong K Ye Y. The acidic microenvironment promotes pancreatic cancer progression via the lncRNA-LOC100507424/E2F1/FOXM1 axis. BMC Cancer. (2025) 25:655. doi: 10.1186/s12885-025-14073-4, PMID: 40211195 PMC11984246

[46] Kwas K Szubert M Wilczyński JR. Latest update on lncRNA in epithelial ovarian cancer-A scoping review. Cells. (2025) 14:555. doi: 10.3390/cells14070555, PMID: 40214508 PMC11988607

[47] Lin Y Pang Q Shi Y Chen X Tu F. Long noncoding RNA MALAT1 promotes angiogenesis through the caveolin-1/VEGF pathway after cerebral ischemic injury. Neuroreport. (2025) 36:350–63. doi: 10.1097/WNR.0000000000002157, PMID: 40203233

[48] Lu Y. Blocking lncRNA NOP14-AS1 overcomes 5-Fu resistance of colon cancer cells by modulating miR-30a-5p-LDHA-glucose metabolism pathway. Discover Oncol. (2025) 16:458. doi: 10.1007/s12672-025-02156-4, PMID: 40180667 PMC11968611

[49] Chen Y Zhu J-Y Hong KH Mikles DC Georg GI Goldstein AS. Structural basis of ALDH1A2 inhibition by irreversible and reversible small molecule inhibitors. ACS Chem Biol. (2018) 13:582–90. doi: 10.1021/acschembio.7b00685, PMID: 29240402 PMC6089219

[50] Beecroft SJ Ayala M McGillivray G Nanda V Agolini E Novelli A. Biallelic hypomorphic variants in ALDH1A2 cause a novel lethal human multiple congenital anomaly syndrome encompassing diaphragmatic, pulmonary, and cardiovascular defects. Hum Mutat. (2021) 42:506–19. doi: 10.1002/humu.24179, PMID: 33565183

[51] Oliveira LDM Teixeira FME Sato MN. Impact of retinoic acid on immune cells and inflammatory diseases. Mediators Inflammation. (2018) 2018:3067126. doi: 10.1155/2018/3067126, PMID: 30158832 PMC6109577

[52] Erbs G Jakobsen JT Schmidt ST Christensen D Bailey M Jungersen G. Retinoic acid-adjuvanted vaccine induces antigen-specific secretory IgA in the gut of newborn piglets. Vaccine. (2025) 46:126672. doi: 10.1016/j.vaccine.2024.126672, PMID: 39733479

[53] Chun TY Carman JA Hayes CE. Retinoid repletion of vitamin A-Deficient mice restores IgG responses. J Nutr. (1992) 122:1062–9. doi: 10.1093/jn/122.5.1062, PMID: 1564559

[54] Hao X Zhong X Sun X. The effects of all-trans retinoic acid on immune cells and its formulation design for vaccines. AAPS J. (2021) 23:32. doi: 10.1208/s12248-021-00565-1, PMID: 33629139

[55] Riccomi A Piccaro G Christensen D Palma C Andersen P Vendetti S. Parenteral vaccination with a tuberculosis subunit vaccine in presence of retinoic acid provides early but transient protection to M. Tuberculosis infection. Front Immunol. (2019) 10:437157. doi: 10.3389/fimmu.2019.00934, PMID: 31130946 PMC6509564

[56] Sadeghzadeh-Bazargan A Behrangi E Goodarzi A. Systemic retinoids in the COVID-19 era – are they helpful, safe, or harmful? a comprehensive systematized review. Iranian J Dermatol. (2020) 23:9–12. doi: 10.22034/ijd.2020.114847

[57] Elkazzaz M Abo-Amer YE-E Ahmed A Haydara T. 13 cis retinoic acid improved the outcomes of COVID-19 patients. A randomized Clin trial. medRxiv. (2022). doi: 10.1101/2022.03.05.22271959

[58] Silveira KC Fonseca IC Oborn C Wengryn P Ghafoor S Beke A. CYP26B1-related disorder: expanding the ends of the spectrum through clinical and molecular evidence. Hum Genet. (2023) 142:1571. doi: 10.1007/s00439-023-02598-2, PMID: 37755482 PMC10602971

[59] Takeuchi H Yokota A Ohoka Y Iwata M. Cyp26b1 regulates retinoic acid-dependent signals in T cells and its expression is inhibited by transforming growth factor-β. PloS One. (2011) 6:e16089. doi: 10.1371/journal.pone.0016089, PMID: 21249211 PMC3017564

[60] Chenery A Burrows K Antignano F Underhill TM Petkovich M Zaph C. The retinoic acid-metabolizing enzyme cyp26b1 regulates CD4 T cell differentiation and function. PloS One. (2013) 8:e72308. doi: 10.1371/journal.pone.0072308, PMID: 23991089 PMC3750006

[61] Ogawa Y Schafer DP Horresh I Bar V Hales K Yang Y. Spectrins and ankyrinB constitute a specialized paranodal cytoskeleton. J Neurosci. (2006) 26:5230–9. doi: 10.1523/JNEUROSCI.0425-06.2006, PMID: 16687515 PMC6674250

[62] Zhang C Susuki K Zollinger DR Dupree JL Rasband MN. Membrane domain organization of myelinated axons requires βII spectrin. J Cell Biol. (2013) 203:437–43. doi: 10.1083/jcb.201308116, PMID: 24217619 PMC3824014

[63] Tang Y Katuri V Dillner A Mishra B Deng C-X Mishra L. Disruption of transforming growth factor-beta signaling in ELF beta-spectrin-deficient mice. Science. (2003) 299:574–7. doi: 10.1126/science.1075994, PMID: 12543979

[64] Nihei Y Haniuda K Higashiyama M Asami S Iwasaki H Fukao Y. Identification of IgA autoantibodies targeting mesangial cells redefines the pathogenesis of IgA nephropathy. Sci Adv. (2023) 9:eadd6734. doi: 10.1126/sciadv.add6734, PMID: 36947618 PMC10032602

[65] Havlicek MG. The role of the host factor SPTBN1 in HIV-1 infection of the role of the host factor SPTBN1 in HIV-1 infection of microglial cells microglial cells.

[66] Dai L-P Xu X-D Yang T-T Yin Z-H Ye Z-Z Wei Y-Z. SPTBN1 attenuates rheumatoid arthritis synovial cell proliferation, invasion, migration and inflammatory response by binding to PIK3R2. Immun Inflammation Dis. (2022) 10:e724. doi: 10.1002/iid3.724, PMID: 36444616 PMC9667201

[67] Van Der Meer LT Jansen JH van der Reijden BA. Gfi1 and Gfi1b: key regulators of hematopoiesis. Leukemia. (2010) 24:1834–43. doi: 10.1038/leu.2010.195, PMID: 20861919

[68] Möröy T Khandanpour C. Growth factor independence 1 (Gfi1) as a regulator of lymphocyte development and activation. Semin Immunol. (2011) 23:368–78. doi: 10.1016/j.smim.2011.08.006, PMID: 21920773

[69] Igwe E Kosan C Khandanpour C Sharif-Askari E Brüne B Möröy T. The zinc finger protein Gfi1 is implicated in the regulation of IgG2b production and the expression of Igamma2b germline transcripts. Eur J Immunol. (2008) 38:3004–14. doi: 10.1002/eji.200838251, PMID: 18991277

[70] Xia Y He F Wu X Tan B Chen S Liao Y. GABA transporter sustains IL-1β production in macrophages. Sci Adv. (2021) 7:eabe9274. doi: 10.1126/sciadv.abe9274, PMID: 33827820 PMC8026138

[71] Street VA McKee-Johnson JW Fonseca RC Tempel BL Noben-Trauth K. Mutations in a plasma membrane Ca2+-ATPase gene cause deafness in deafwaddler mice. Nat Genet. (1998) 19:390–4. doi: 10.1038/1284, PMID: 9697703

[72] Ficarella R Di Leva F Bortolozzi M Ortolano S Donaudy F Petrillo M. A functional study of plasma-membrane calcium-pump isoform 2 mutants causing digenic deafness. Proc Natl Acad Sci U.S.A. (2007) 104:1516–21. doi: 10.1073/pnas.0609775104, PMID: 17234811 PMC1785272

[73] Merino-Wong M Niemeyer BA Alansary D. Plasma membrane calcium ATPase regulates stoichiometry of CD4+ T-cell compartments. Front Immunol. (2021) 12:687242. doi: 10.3389/fimmu.2021.687242, PMID: 34093590 PMC8175910

[74] López-Bielma MF Falfán-Valencia R Fierro-Piña A Abarca-Rojano E Córdoba-Lanus E Fricke-Galindo I. Genetic variants in ATP2B2 as risk factors for mortality in patients unrelated but not associated with families with severe COVID-19. Heliyon. (2024) 10:e29493. doi: 10.1016/j.heliyon.2024.e29493, PMID: 38628728 PMC11019202

[75] Ishida S Kato K Tanaka M Odamaki T Kubo R Mitsuyama E. Genome-wide association studies and heritability analysis reveal the involvement of host genetics in the Japanese gut microbiota. Commun Biol. (2020) 3:686. doi: 10.1038/s42003-020-01416-z, PMID: 33208821 PMC7674416

[76] Rühlemann MC Hermes BM Bang C Doms D Moitinho-Silva L Thingholm LB. Genome-wide association study in 8,956 German individuals identifies influence of ABO histo-blood groups on gut microbiome. Nat Genet. (2021) 53:147–55. doi: 10.1038/s41588-020-00747-1, PMID: 33462482

[77] Weaver D Gago S Bassetti M Ciacobbe DR Prattes J Hoenigl M. Mycobiome analyses of critically ill COVID-19 patients. Microbiol Spectr. (2025) 13:e0411023. doi: 10.1128/spectrum.04110-23, PMID: 39699254 PMC11792475

[78] Guha SK Niyogi S. Microbial dynamics in COVID-19: unraveling the impact of human microbiome on disease susceptibility and therapeutic strategies. Curr Microbiol. (2024) 82:59. doi: 10.1007/s00284-024-04041-9, PMID: 39720963

[79] Bhanu P Buchke S Hemandhar-Kumar N Varsha P Kiran SKR Vikneswaran G. Comparative metagenomic analysis of the oral microbiome in COVID-19 patients and healthy individuals. Sci Rep. (2025) 15:10303. doi: 10.1038/s41598-024-81864-3, PMID: 40133298 PMC11937335

[80] Ng HY Liao Y Cheung CL Zhang R Chan KH Seto W-K. Gut microbiota is associated with persistence of longer-term BNT162b2 vaccine immunogenicity. Front Immunol. (2025) 16. doi: 10.3389/fimmu.2025.1534787, PMID: 40083550 PMC11903479

[81] Dutta S Chatterjee N Gallina NLF Kar S Koley H Nanda PK. Diet, microbiome, and probiotics establish a crucial link in vaccine efficacy. Crit Rev Microbiol. (2025) 20:1–26. doi: 10.1080/1040841X.2025.2480230, PMID: 40110742

[82] Worby CA Dixon JE. Sorting out the cellular functions of sorting nexins. Nat Rev Mol Cell Biol. (2002) 3:919–31. doi: 10.1038/nrm974, PMID: 12461558

[83] Astle WJ Elding H Jiang T Allen D Ruklisa D Mann AL. The allelic landscape of human blood cell trait variation and links to common complex disease. Cell. (2016) 167:1415–29.e19. doi: 10.1016/j.cell.2016.10.042, PMID: 27863252 PMC5300907

[84] Vuckovic D Bao EL Akbari P Lareau CA Mousas A Jiang T. The polygenic and monogenic basis of blood traits and diseases. Cell. (2020) 182:1214–31.e11. doi: 10.1016/j.cell.2020.08.008, PMID: 32888494 PMC7482360

[85] Choudry FA Bagger FO Macaulay IC Farrow S Burden F Kempster C. Transcriptional characterization of human megakaryocyte polyploidization and lineage commitment. J Thromb Haemost. (2021) 19:1236–49. doi: 10.1111/jth.15271, PMID: 33587817

[86] Lacey J Webster SJ Heath PR Hill CJ Nicholson-Goult L Wagner BE. Sorting nexin 24 is required for α-granule biogenesis and cargo delivery in megakaryocytes. Haematologica. (2022) 107:1902. doi: 10.3324/haematol.2021.279636, PMID: 35021601 PMC9335091

[87] Kaldis P Pagano M. Wnt signaling in mitosis. Dev Cell. (2009) 17:749–50. doi: 10.1016/j.devcel.2009.12.001, PMID: 20059944

[88] Davidson G Niehrs C. Emerging links between CDK cell cycle regulators and Wnt signaling. Trends Cell Biol. (2010) 20:453–60. doi: 10.1016/j.tcb.2010.05.002, PMID: 20627573

[89] Chen J-W Wang Y-X Gao R-R Ma L-Y Zhong J Yang J-X. CDK14 regulates the development and repair of lung. Cell Death Discov. (2025) 11:1–14. doi: 10.1038/s41420-025-02292-4, PMID: 39827158 PMC11743204

[90] Chen S Guan F Candotti F Benlagha K Camara NOS Herrada AA. The role of B cells in COVID-19 infection and vaccination. Front Immunol. (2022) 13:988536. doi: 10.3389/fimmu.2022.988536, PMID: 36110861 PMC9468879

[91] Rajamanickam A Kumar NP Nancy P A Selvaraj N Munisankar S Renji RM. Recovery of memory B-cell subsets and persistence of antibodies in convalescent COVID-19 patients. Am J Trop Med Hyg. (2021) 105:1255. doi: 10.4269/ajtmh.21-0883, PMID: 34583334 PMC8592221

[92] Ciabattini A Pastore G Fiorino F Polvere J Lucchesi S Pettini E. Evidence of SARS-CoV-2-specific memory B cells six months after vaccination with the BNT162b2 mRNA vaccine. Front Immunol. (2021) 12:740708. doi: 10.3389/fimmu.2021.740708, PMID: 34650563 PMC8505800

[93] Lin YC Jhunjhunwala S Benner C Heinz S Welinder E Mansson R. A global network of transcription factors, involving E2A, EBF1 and Foxo1, that orchestrates the B cell fate. Nat Immunol. (2010) 11:635. doi: 10.1038/ni.1891, PMID: 20543837 PMC2896911

[94] Wöhner M Tagoh H Bilic I Jaritz M Kostanova D Fischer M. Molecular functions of the transcription factors E2A and E2-2 in controlling germinal center B cell and plasma cell development. J Exp Med. (2016) 213:1201. doi: 10.1084/jem.20152002, PMID: 27261530 PMC4925024

[95] Wikström I Forssell J Goncalves M Colucci F Holmberg D. E2-2 regulates the expansion of pro-B cells and follicular versus marginal zone decisions. J Immunol. (2006) 177:6723–9. doi: 10.4049/jimmunol.177.10.6723, PMID: 17082585

[96] Zhang B Zhang Z Koeken VACM Kuman S Aillaud M Tsay H-C. Altered and allele-specific open chromatin landscape reveals epigenetic and genetic regulators of innate immunity in COVID-19. Cell Genomics. (2023) 3:100232. doi: 10.1016/j.xgen.2022.100232, PMID: 36474914 PMC9715265

[97] Zhang L Nishi H Kinoshita K. Single-cell RNA-seq public data reveal the gene regulatory network landscape of respiratory epithelial and peripheral immune cells in COVID-19 patients. Front Immunol. (2023) 14. doi: 10.3389/fimmu.2023.1194614, PMID: 37936693 PMC10627007

[98] Amrute JM Perry AM Anand G Cruchaga C Hock KG Farnsworth CW. Cell specific peripheral immune responses predict survival in critical COVID-19 patients. Nat Commun. (2022) 13:882. doi: 10.1038/s41467-022-28505-3, PMID: 35169146 PMC8847593

[99] Geng X Xia X Liang Z Li S Yue Z Zhang H. Tropomodulin1 exacerbates inflammatory response in macrophages by negatively regulating LPS-induced TLR4 endocytosis. Cell Mol Life Sci. (2024) 81:402. doi: 10.1007/s00018-024-05424-8, PMID: 39276234 PMC11401823

[100] Liu X Xia X Wang X Zhou J Sung LA Long J. Tropomodulin1 expression increases upon maturation in dendritic cells and promotes their maturation and immune functions. Front Immunol. (2021) 11:587441. doi: 10.3389/fimmu.2020.587441, PMID: 33552047 PMC7856346

[101] Camp CR Vlachos A Klöckner C Krey I Banke TG Shariatzadeh N. Loss of Grin2a causes a transient delay in the electrophysiological maturation of hippocampal parvalbumin interneurons. Commun Biol. (2023) 6. doi: 10.1038/s42003-023-05298-9, PMID: 37723282 PMC10507040

[102] Strehlow V Heyne HO Vlaskamp DRM Marwick KFM Rudolf G de Bellescize J. GRIN2A-related disorders: genotype and functional consequence predict phenotype. Brain. (2019) 142:80–92. doi: 10.1093/brain/awy304, PMID: 30544257 PMC6308310

[103] Yasmin F Marwick KFM Hunter DW Nawaz S Marshall GF Booker SA. Absence of GluN2A in hippocampal CA1 neurons leads to altered dendritic structure and reduced frequency of miniature excitatory synaptic events. Brain Commun. (2025) 7:fcaf124. doi: 10.1093/braincomms/fcaf124, PMID: 40226380 PMC11986202

[104] Xu J Wei H You P Sui J Xiu J Zhu W. Non-neutralizing antibodies to SARS-Cov-2-related linear epitopes induce psychotic-like behavior in mice. Front Mol Neurosci. (2023) 16. doi: 10.3389/fnmol.2023.1177961, PMID: 37138704 PMC10149951

[105] Wang J Sun D Wang Y Ren F Pang S Wang D. FOSL2 positively regulates TGF-β1 signalling in non-small cell lung cancer. PLoS One. (2014) 9:e112150. doi: 10.1371/journal.pone.0112150, PMID: 25375657 PMC4223012

[106] Yang X Letterio JJ Lechleider RJ Chen L Hayman R Gu H. Targeted disruption of SMAD3 results in impaired mucosal immunity and diminished T cell responsiveness to TGF-β. EMBO J. (1999) 18:1280–91. doi: 10.1093/emboj/18.5.1280, PMID: 10064594 PMC1171218

[107] Malhotra N Kang J. SMAD regulatory networks construct a balanced immune system. Immunology. (2013) 139:1. doi: 10.1111/imm.12076, PMID: 23347175 PMC3634534

[108] Zhao X Nicholls JM Chen YG. Severe acute respiratory syndrome-associated coronavirus nucleocapsid protein interacts with smad3 and modulates transforming growth factor-β Signaling. J Biol Chem. (2021) 283:3272. doi: 10.1074/jbc.M708033200, PMID: 18055455 PMC8740907

[109] Liang L Wang W Chen J Wu W Huang X-R Wei B. SARS-CoV-2 N protein induces acute kidney injury in diabetic mice via the Smad3-Ripk3/MLKL necroptosis pathway. Signal Transduction Targeted Ther. (2023) 8:1–4. doi: 10.1038/s41392-023-01410-x, PMID: 37029116 PMC10080522

[110] Wang W Chen J Hu D Pan P Lliang L Wu W. SARS-CoV-2 N protein induces acute kidney injury via Smad3-dependent G1 cell cycle arrest mechanism. Advanced Sci. (2021) 9:2103248. doi: 10.1002/advs.202103248, PMID: 34813685 PMC8787402

[111] Chen M Yao C Qin Y Cui X Li P Ji Z. SARS-CoV-2 nucleocapsid protein triggers hyperinflammation via protein-protein interaction-mediated intracellular Cl– accumulation in respiratory epithelium. Signal Transduction Targeted Ther. (2022) 7:1–13. doi: 10.1038/s41392-021-00710-4, PMID: 35896532 PMC9328007

[112] Mukund K Nayak P Ashokkumar C Rao S Almeda J Betancourt-Garcia MM. Immune response in severe and non-severe coronavirus disease 2019 (COVID-19) infection: A mechanistic landscape. Front Immunol. (2021) 12:738073. doi: 10.3389/fimmu.2021.738073, PMID: 34721400 PMC8548832

[113] Klenke S Rump K Buschkamp K Engler A Peters J Siffert W. Characterization of the PLCB1 promoter and regulation by early growth response transcription factor EGR-1. Eur J Pharmacol. (2014) 742:8–14. doi: 10.1016/j.ejphar.2014.08.026, PMID: 25192965

[114] Lin Y-J Chang J-S Liu X Tsang H Chien W-K Chen J-H. Genetic variants in PLCB4/PLCB1 as susceptibility loci for coronary artery aneurysm formation in Kawasaki disease in Han Chinese in Taiwan. Sci Rep. (2015) 5:1–12. doi: 10.1038/srep14762, PMID: 26434682 PMC4593004

[115] Reyes RA Clarke K Gonzales SJ Cantwell AM Garza R Catano G. SARS-CoV-2 spike-specific memory B cells express higher levels of T-bet and FcRL5 after non-severe COVID-19 as compared to severe disease. PLoS One. (2021) 16:e0261656. doi: 10.1371/journal.pone.0261656, PMID: 34936684 PMC8694470

[116] García-Vega M Llamas-Covarrubias MA Loza M Reséndiz-Sandoval M Hinojosa-Trujillo D Melgoza-González E. Single-cell transcriptomic analysis of B cells reveals new insights into atypical memory B cells in COVID-19. J Med Virol. (2024) 96:e29851. doi: 10.1002/jmv.29851, PMID: 39132689

[117] Kleiber N Lemus-Diaz N Stiller C Heinrichs M Mai MM-Q Hackert P. The RNA methyltransferase METTL8 installs m3C32 in mitochondrial tRNAsThr/Ser(UCN) to optimise tRNA structure and mitochondrial translation. Nat Commun. (2022) 13:1–19. doi: 10.1038/s41467-021-27905-1, PMID: 35017528 PMC8752778

[118] Huang M-H Peng G-X Mao X-L Wang J-T Zhou J-B Zhang J-H. Molecular basis for human mitochondrial tRNA m3C modification by alternatively spliced METTL8. Nucleic Acids Res. (2022) 50:4012. doi: 10.1093/nar/gkac184, PMID: 35357504 PMC9023283

[119] Li Y Jin H Li Q Shi L Mao Y Zhao L. The role of RNA methylation in tumor immunity and its potential in immunotherapy. Mol Cancer. (2024) 23:130. doi: 10.1186/s12943-024-02041-8, PMID: 38902779 PMC11188252

[120] Zhang K Eldin P Ciesla JH Briant L Lentini JM Ramos J. Proteolytic cleavage and inactivation of the TRMT1 tRNA modification enzyme by SARS-CoV-2 main protease. Elife. (2024) 12:RP90316. doi: 10.7554/eLife.90316.2 38814682 PMC11139479

[121] Chen R Cao J Jiang W Wang S Cheng J. Upregulated expression of CYBRD1 predicts poor prognosis of patients with ovarian cancer. J Oncol. (2021) 2021:7548406. doi: 10.1155/2021/7548406, PMID: 34594380 PMC8478559

[122] Chen ZZ Johnson L Trahtemberg U Baker A Huq S Dufresne J. Mitochondria and cytochrome components released into the plasma of severe COVID-19 and ICU acute respiratory distress syndrome patients. Clin Proteomics. (2023) 20:17. doi: 10.1186/s12014-023-09394-0, PMID: 37031181 PMC10082440

[123] Tang M Li Y Luo X Xiao J Wang J Zeng X. Identification of biomarkers related to CD8+ T cell infiltration with gene co-expression network in lung squamous cell carcinoma. Front Cell Dev Biol. (2021) 9:606106. doi: 10.3389/fcell.2021.606106, PMID: 33816462 PMC8012856

[124] Xie J Mothe B Alcalde Herraiz M Li C Xu Y Jödicke AM. Relationship between HLA genetic variations, COVID-19 vaccine antibody response, and risk of breakthrough outcomes. Nat Commun. (2024) 15:4031. doi: 10.1038/s41467-024-48339-5, PMID: 38740772 PMC11091043

[125] Demonbreun AR Sancilio A Velez ME Ryan DT Pesce L Saber R. COVID-19 mRNA vaccination generates greater immunoglobulin G levels in women compared to men. J Infect Dis. (2021) 224:793–7. doi: 10.1093/infdis/jiab314, PMID: 34117873 PMC8536925

[126] Jensen A Stromme M Moyassari S Chadha AS Tartaglia MC Szoeke C. COVID-19 vaccines: Considering sex differences in efficacy and safety. Contemp Clin Trials. (2022) 115:106700. doi: 10.1016/j.cct.2022.106700, PMID: 35149232 PMC8824304

[127] Bachmann M Gültekin N Stanga Z Fehr JS Ülgür II Schlagenhauf P. Disparities in response to mRNA SARS-CoV-2 vaccines according to sex and age: A systematic review. New Microbes New Infect. (2024) 63:101551. doi: 10.1016/j.nmni.2024.101551, PMID: 39807161 PMC11726804

[128] Jay C Adland E Csala A Lim N Longet S Ogbe A. Age- and sex-specific differences in immune responses to BNT162b2 COVID-19 and live-attenuated influenza vaccines in UK adolescents. Front Immunol. (2023) 14:1248630. doi: 10.3389/fimmu.2023.1248630, PMID: 37942333 PMC10627794

