# Supplementary material for: A genome-wide association study identifies new loci associated with response to SARS-CoV-2 mRNA-1273 vaccine in a cohort of healthy healthcare workers
Source: Front Immunol. 2025 Aug 18;16:1639825. doi: 10.3389/fimmu.2025.1639825 (PMC12409172; doi:10.3389/fimmu.2025.1639825)
Supplement: Supplementary file 1 [file DataSheet1.pdf]

Supplementary Figure 1. Quantile-Quantile (QQ) Plots for GWAS Analyses.

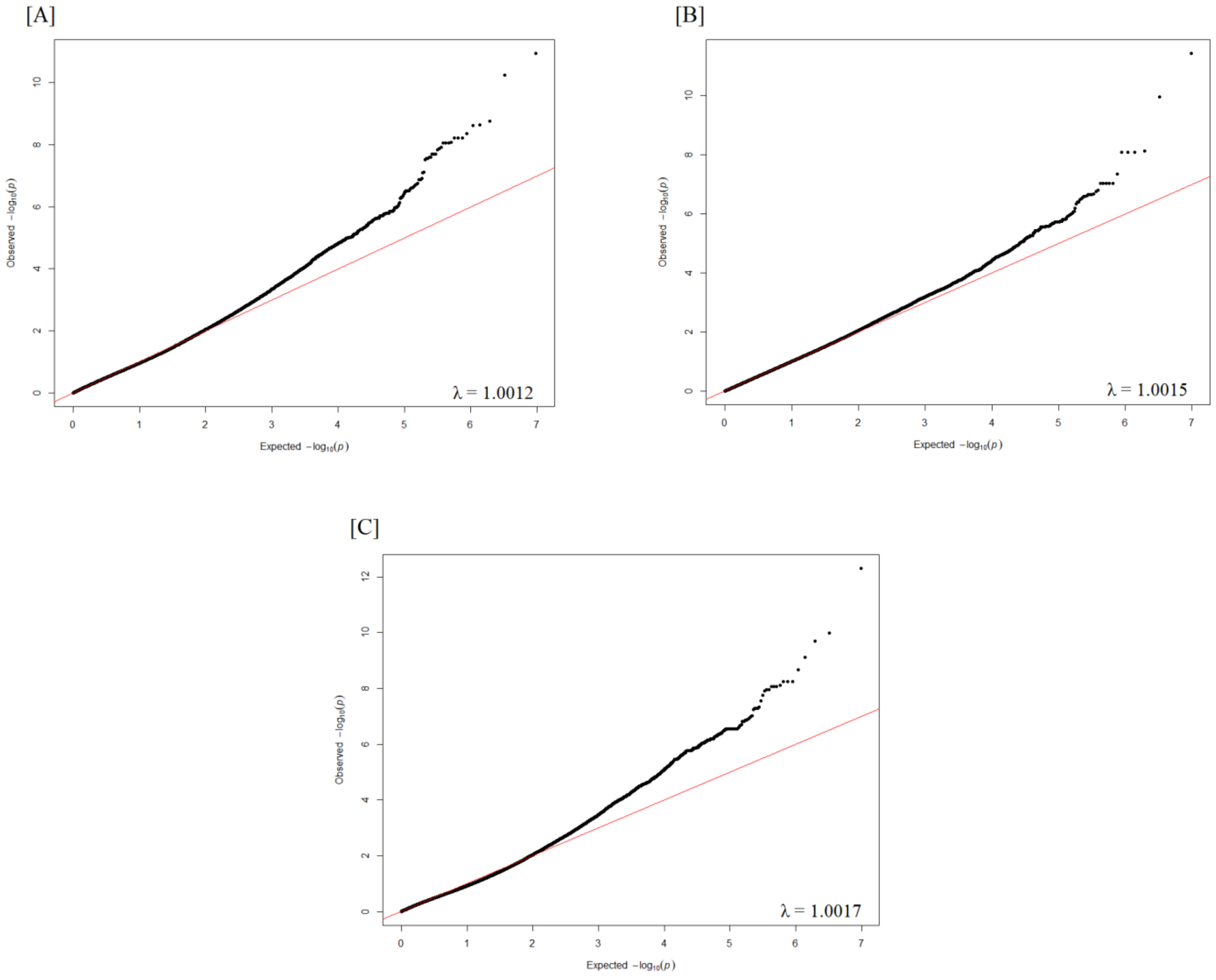

[A] QQ-Plot for GWAS 1: The GWAS analysis of IgG levels measured at the first month after mRNA-1273 vaccination; [B] QQ-Plot for GWAS 2: The GWAS analysis of IgG levels measured at the third month after mRNA-1273 vaccination; [C] QQ-Plot for GWAS 3: The GWAS analysis of the difference in IgG levels between the first and third months after mRNA-1273 vaccination. Expected (under the null hypothesis of no association) and observed distributions of -log10(P) values are shown on the x-axis and y-axis, respectively. The red line corresponds to y = x. Inflation lambda (λ) is the observed median χ^2 test statistic divided by the median expected χ^2 test statistic under the null hypothesis, indicating the genomic inflation.
