# Supplementary material for: A genome-wide association study identifies new loci associated with response to SARS-CoV-2 mRNA-1273 vaccine in a cohort of healthy healthcare workers
Source: Front Immunol. 2025 Aug 18;16:1639825. doi: 10.3389/fimmu.2025.1639825 (PMC12409172; doi:10.3389/fimmu.2025.1639825)
Supplement: Supplementary file 3 [file DataSheet3.pdf]

Supplementary Figure 3. Circos plot displaying genomic risk *loci* and associated features across chromosomes in GWAS2.

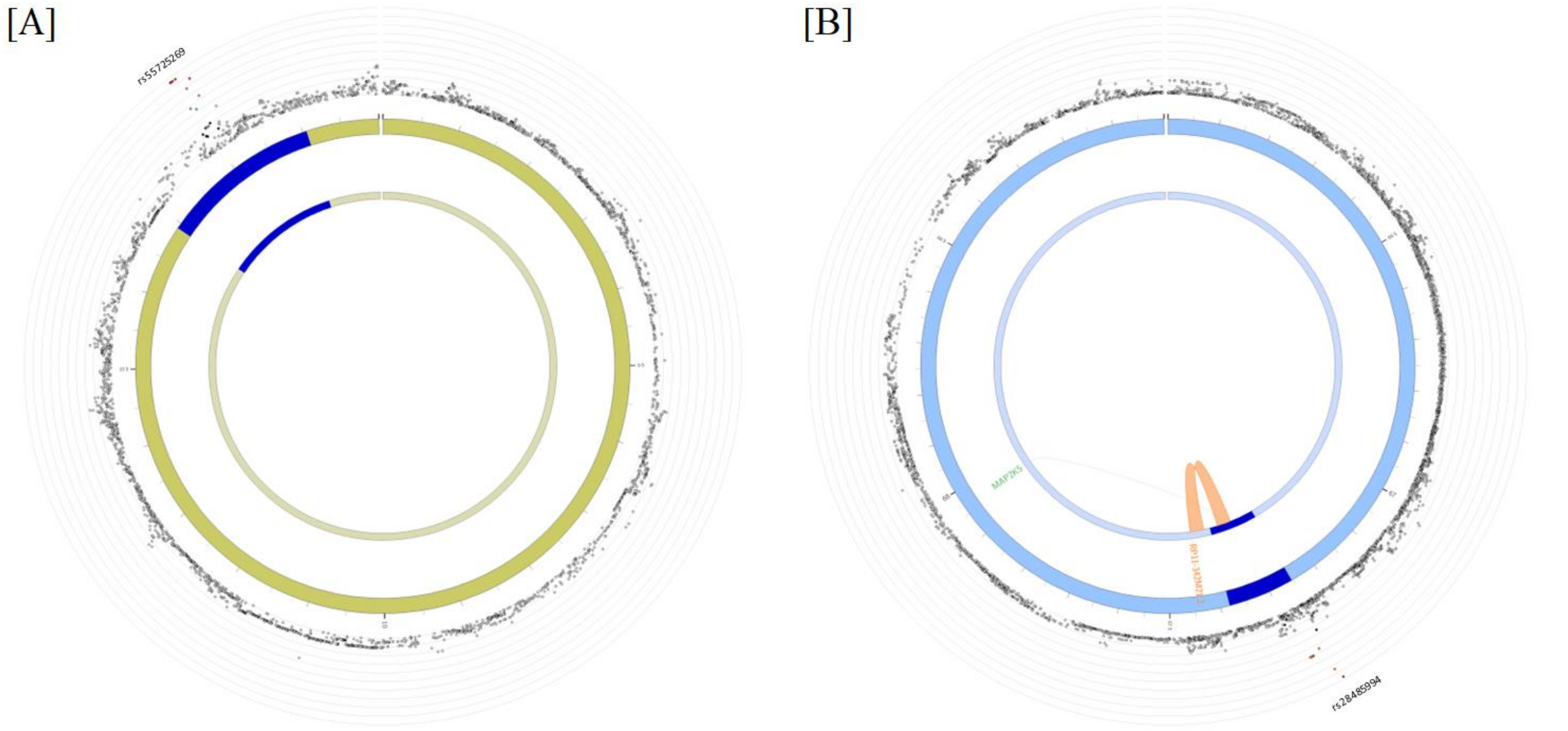

The circos plot shows chromosomes [A] 3; [B] 15. The outermost layer is a Manhattan plot, displaying SNPs with $P < 0.05$, color-coded by r^2 to the independent significant SNP in the locus. The second layer highlights genomic risk loci in blue. The third layer displays mapped genes based on chromatin interactions (orange) or eQTLs (green), with red for genes mapped by both. Interaction links are shown in orange (chromatin) and green (eQTL).
