# Supplementary material for: A genome-wide association study identifies new loci associated with response to SARS-CoV-2 mRNA-1273 vaccine in a cohort of healthy healthcare workers
Source: Front Immunol. 2025 Aug 18;16:1639825. doi: 10.3389/fimmu.2025.1639825 (PMC12409172; doi:10.3389/fimmu.2025.1639825)

Supplementary Figure 5. Violin plots showing the strong association of the *SNX24*rs55770715 and *METTL8*rs1125991 SNPs with *SNX24* and *CYBRD1* mRNA expression levels in whole blood.

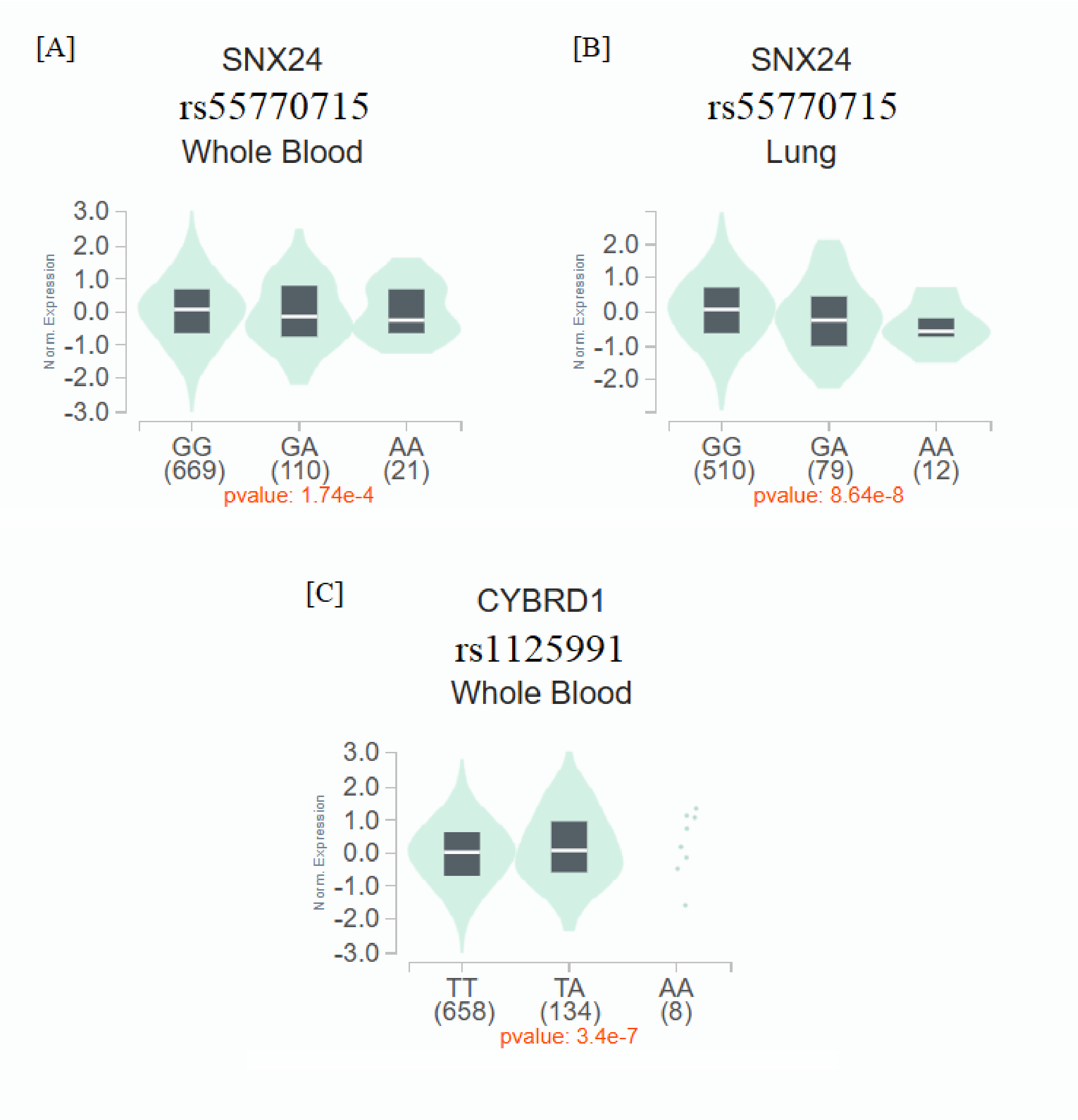

Supplement: Supplementary file 5 [file DataSheet5.pdf]
