# Supplementary material for: A genome-wide association study identifies new loci associated with response to SARS-CoV-2 mRNA-1273 vaccine in a cohort of healthy healthcare workers
Source: Front Immunol. 2025 Aug 18;16:1639825. doi: 10.3389/fimmu.2025.1639825 (PMC12409172; doi:10.3389/fimmu.2025.1639825)
Supplement: Supplementary file 7 [file DataSheet7.pdf]

Supplementary Table 2. Cell types analysed either in whole blood or peripheral mononuclear blood cells.

Number	Name	Parent	Grandparent
1	Leukocytes_CD45P_LMI1	None	None
2	Neutrophils_LMI1	1	None
3	Monocytes_CD14P_LMI1	1	None
4	Classical_monocytes_CD14PPCD16N_LMI1	3	1
5	Intermediate_monocytes_CD14PPCD16P_LMI1	3	1
6	NonClassical_monocytes_CD14PCD16P_LMI1	3	1
7	Lymphocytes_LMI1	1	None
8	T_cells_CD3P_CD56N_LMI1	7	1
9	NK_cells_CD3N_CD56P_LMI1	7	1
10	NKT_cells_CD3P_CD56P_LMI1	7	1
11	B_cells_CD19P_LMI1	7	1
12	CD4P_T_cells_LMI1	8	7
13	CD8P_T_cells_LMI1	8	7
14	DP_CD4P_CD8P_LMI1	8	7
15	DN_CD4N_CD8N_LMI1	8	7
16	CD4P_CD25high_Treg_LMI1	12	8
17	NK_dim_CD56P_CD16P_LMI1	9	7
18	NK_bright_CD56PP_CD16N_LMI1	9	7
19	NK_CD56P_CD16N_LMI1	9	7
20	Leukocytes_CD45P_LMI2	justRef	justRef
21	T_cells_CD3P_CD56N_LMI2	justRef	justRef
22	CD4P_T_cells_LMI2	justRef	justRef
23	CD8P_T_cells_LMI2	justRef	justRef
24	CD45RON_CD45RAP_T_cells_LMI2	21	20
25	CD45ROP_CD45RAP_T_cells_LMI2	21	20
26	CD45ROP_CD45RAN_T_cells_LMI2	21	20
27	CD45RON_CD45RAN_T_cells_LMI2	21	20
28	CD4P_CD25P_CD127low_Treg_LMI2	22	21
29	CD4P_Naive_CD45RAP_CD27P_LMI2	22	21
30	CD4P_Eff_CD45RAP_CD27N_LMI2	22	21
31	CD4P_EM_CD45RAN_CD27N_LMI2	22	21
32	CD4P_CM_CD45RAN_CD27P_LMI2	22	21
33	CD4P_Naive_CD45RON_CD27P_LMI2	22	21
34	CD4P_Eff_CD45RON_CD27N_LMI2	22	21
35	CD4P_EM_CD45ROP_CD27N_LMI2	22	21
36	CD4P_CM_CD45ROP_CD27P_LMI2	22	21
37	CD8P_Naive_CD45RAP_CD27P_LMI2	23	21
38	CD8P_Eff_CD45RAP_CD27N_LMI2	23	21
39	CD8P_EM_CD45RAN_CD27N_LMI2	23	21
40	CD8P_CM_CD45RAN_CD27P_LMI2	23	21
41	CD8P_Naive_CD45RON_CD27P_LMI2	23	21
42	CD8P_Eff_CD45RON_CD27N_LMI2	23	21
43	CD8P_EM_CD45ROP_CD27N_LMI2	23	21
44	CD8P_CM_CD45ROP_CD27P_LMI2	23	21
45	Lymphocytes_LMI3	justRef	justRef
46	B_cells_CD3N_CD19P_LMI3	justRef	justRef

47	CD27N_IgMP_LMI3	justRef	justRef
48	CD19P_CD20N_Plasma_blasts_LMI3	47	46
49	CD19P_CD20P_B_cells_LMI3	47	46
50	IgDN_CD5PP_LMI3	47	46
51	IgDP_CD5PP_LMI3	47	46
52	IgDN_CD5P_LMI3	47	46
53	IgDP_CD5P_LMI3	47	46
54	CD24P_CD38P_LMI3	47	46
55	Transitional_B_cells_CD24PP_CD38PP_LMI3	47	46
56	IgDN_IgMP_LMI3	47	46
57	IgDP_IgMP_LMI3	47	46
58	IgDP_IgMN_LMI3	47	46
59	IgDN_IgMN_LMI3	47	46
60	Transitional_B_cell_CD27N_IgMP_CD24P_CD38high_LMI3	48	47
61	Mature_naive_CD24P_CD38P_LMI3	48	47
62	IgMN_LMI3	55	47
63	CD24P_CD38P_CD27P_IgMP_LMI3	55	47
64	Natural_effector_CD24P_CD38P_IgDP_IgMP_LMI3	55	47
65	Plasmablast_IgDN_IgMN_CD38PP_LMI3	60	47
66	Class_switched_memory_IgDN_IgMN_CD38P_CD27P_LMI3	60	47
67	IgDN_IgMN_CD27N_LMI3	60	47
68	Naive_B_cells_IgDP_IgMP_CD27N_LMI3	58	47
69	Memory_B_cells_IgDP_IgMP_CD27P_LMI3	58	47
70	IgDN_IgMP_CD27N_LMI3	57	47
71	IgM_only_memory_IgDN_IgMP_CD27_LMI3	57	47
72	IgMP_CD38PP_CD27P_LMI3	57+58	47
73	Class_non_switched_memory_IgMP_CD38P_CD27P_LMI3	57+58	47
74	IgMP_CD27N_LMI3	57+58	47
75	Lymphocytes_PBMC_LMI4	justRef	justRef
76	CD4P_T_cells_PBMC_LMI4	justRef	justRef
77	DP_CD4P_CD8P_PBMC_LMI4	justRef	justRef
78	DN_CD4N_CD8N_PBMC_LMI4	justRef	justRef
79	CD8P_T_cells_PBMC_LMI4	justRef	justRef
80	CD25N_CD127P_Tconv_PBMC_LMI4	justRef	justRef
81	CD4P_CD25P_CD127low_Treg_PBMC_LMI4	justRef	justRef
82	Prol_DN_CD4NCD8N_PBMC_LMI4	80	77
83	Prol_DP_CD4PCD8P_PBMC_LMI4	79	77
84	Prol_CD4P_Tconv_PBMC_LMI4	82	78
85	Prol_CD4P_Treg_PBMC_LMI4	83	78
86	Prol_CD8_PBMC_LMI4	81	77
87	Treg_FOXP3P_HeliosP_PBMC_LMI4	83	78
88	Treg_FOXP3P_HeliosN_PBMC_LMI4	83	78
89	Treg_CD45RAP_PBMC_LMI4	83	78
90	Treg_CD45RAN_PBMC_LMI4	83	78
91	Treg_HLANDRP_PBMC_LMI4	83	78

NOTE: Cells included in LMI1-3 were measured in whole blood and those included LIM4 in PBMCs.
