# Supplementary material for: A genome-wide association study identifies new loci associated with response to SARS-CoV-2 mRNA-1273 vaccine in a cohort of healthy healthcare workers
Source: Front Immunol. 2025 Aug 18;16:1639825. doi: 10.3389/fimmu.2025.1639825 (PMC12409172; doi:10.3389/fimmu.2025.1639825)
Supplement: Supplementary file 8 [file DataSheet8.pdf]

Supplementary Table 4. Summary of in silico functional and regulatory annotation of novel significant SNPs.

SNP	Chr	Position	Nearest Genes	Consequence	CADDa		RDBa	
					Score	Consequence	Score	Rank
rs1350209880	15	58183817	ENSG00000295231	LncRNA			7	0.1841
rs72845602 δ	2	72347224	CYP26B1	Downstream gene variant	0.397	Probably benign	5	0.0644
rs4088054 γ	3	10808633	LINC00606 | SLC6A11	Intergenic | Upstream gene variant	0.095	Probably benign	1f	0.5544
rs117643807	9	100352774	TMOD1	Intron variant	0.778	Probably benign	1f	0.5544
rs7792239	7	90808856	CDK14	Intron variant	0.624	Probably benign	7	0.1841
rs28485994	15	67257395	SMASR | SMAD3-DT	Intergenic variant	3.742	Probably benign	4	0.6091
rs7907582	10	118144424	CCDC172 | PNLIPRP3	Intergenic variant	13.02	Probably malignant; need more study	7	0.1841
rs34340658	16	10129731	GRIN2A | LOC105371076	Intron variant	1.983	Probably benign	1f	0.3626
rs55770715	5	122347325	SNX24 | LOC124901213	Intergenic variant	0.86	Probably benign	1f	0.2392
rs55725269	3	10799545	ATP2B2	Downstream gene variant	1.445	Probably benign	1f	0.5544
rs75197984	2	54765683	SPTBN1	Intron variant	11.17	Probably malignant; need more study	1f	0.5532
rs1125991	2	172263448	METTL8	Intron variant	1.71	Probably benign	7	0.5139
rs4630616	17	76330484	LOC105371912	Intron variant	0.148	Probably benign	2b	0.4248
rs55919500	20	8072501	PLCB1	Intron variant	0.851	Probably benign	1f	0.5726

Abbreviations: SNP, single nucleotide polymorphism; Chr, Chromosome; CADD, Combined annotation Dependent Depletion; RDB, Regulome DataBase; ChromHMM , Chromatin Hidden Markov Model; C

δ rs72845602 and rs75197984 are in modest linkage disequilibrium (D’ = 0.601; r2 = 0.001).

γ rs4088054 and rs55725269 are in complete linkage disequilibrium with the rs3745990 (D’ = 1.00; r2 = 0.584).

a Functional annotation from Combined Annotation Dependent Depletion (https://cadd.gs.washington.edu/) and RegulomeDB (https://regulomedb.org/)

b Data were performed using the Functional Mapping and Annotation of Genome-Wide Association Studies tool (https://fuma.ctglab.nl/)

c Data from Haploreg v4.2 (https://pubs.broadinstitute.org/mammals/haploreg/haploreg.php)

d Data extracted from the NHGRI-EBI Catalog of human genome-wide association studies (https://www.ebi.ac.uk/gwas/)

e QTL data from QTLBase (http://www.mulinlab.org/qtlbase) and GTEx Portal (https://gtexportal.org/home/)

	Chromatin state predicted by ChromHMM software b	
Consequence	Tissue	Score
No evidence that the variant has a regulatory impact		
Variants in potential regulatory regions based on computational models		
eQTL + TF binding/DNase peak	Primary T cells from cord blood	13
	Primary hematopoietic stem cells short term culture	13
	Lung	9
eQTL + TF binding/DNase peak	Primary T regulatory cells from peripheral blood	5
	Primary mononuclear cells from peripheral blood	5
	Lung	5
No evidence that the variant has a regulatory impact	Primary B cells from cord blood	5
Variants with some regulatory features, but without direct evidence		
No evidence that the variant has a regulatory impact		
eQTL + TF binding/DNase peak		
eQTL + TF binding/DNase peak	Primary B cells from cord blood	9
	Primary mononuclear cells from peripheral blood	5
	NHLF Lung Fibroblast Primary Cells	5
eQTL + TF binding/DNase peak	Primary T cells from peripheral blood	13
eQTL + TF binding/DNase peak	Primary hematopoietic stem cells	5
	Primary hematopoietic stem cells short term culture	5
	Primary hematopoietic stem cells G-CSF-mobilized Female	5
	Primary hematopoietic stem cells G-CSF-mobilized Male	5
	Lung	5
	Spleen	5
	NHLF Lung Fibroblast Primary Cells	5
No evidence that the variant has a regulatory impact	Primary B cells from cord blood	7
	Primary B cells from peripheral blood	7
	Primary T cells from cord blood	5
	Primary hematopoietic stem cells	7
	Primary hematopoietic stem cells short term culture	7
	Primary T helper memory cells from peripheral blood 2	7
	Primary T helper memory cells from peripheral blood 1	5
	Primary T helper cells PMA-I stimulated	5
	Chromatin state predicted by ChromHMM software b	
Consequence	Tissue	Score
	Primary T helper 17 cells PMA-I stimulated	7
	Primary T helper cells from peripheral blood	5
	Primary hematopoietic stem cells G-CSF-mobilized Female	7
	Primary hematopoietic stem cells G-CSF-mobilized Male	7
	NHLF Lung Fibroblast Primary Cells	5
TF binding + any motif + DNase footprint	Primary B cells from peripheral blood	5
	Primary T cells from cord blood	5
	Primary T cells from peripheral blood	5
	Primary hematopoietic stem cells	5
	Primary hematopoietic stem cells short term culture	5
	Primary T helper memory cells from peripheral blood 2	5
	Primary T helper naive cells from peripheral blood	5
	Primary T helper naive cells from peripheral blood	5
	Primary T helper memory cells from peripheral blood 1	5
	Primary T helper cells PMA-I stimulated	5
	Primary T helper 17 cells PMA-I stimulated	7
	Primary T helper cells from peripheral blood	5
	Primary T regulatory cells from peripheral blood	5
	Primary T cells effector/memory enriched from peripheral blood	5
	Primary Natural Killer cells from peripheral blood	5
	Primary T CD8+ memory cells from peripheral blood	5
	Primary hematopoietic stem cells G-CSF-mobilized Male	5
	Lung	5
	Spleen	5
	NHLF Lung Fibroblast Primary Cells	5
eQTL + TF binding/DNase peak		

QTL, Quantitative Trait Locus; TF, Transcription factor.

Histone marks c

Consequence	Tissue	Marks
H3K9me3 and/or H3K27me3 signal. Generally inactive and repetitive regions.	Primary T CD8+ naive cells from peripheral blood	H3K9ac_Pro
H3K9me3 and/or H3K27me3 signal. Generally inactive and repetitive regions.		
	Spleen	H3K4me3_Pro
High H3K36me3 signal. Transcription elongation regions.		
High signal of H3K4me1 and H3K27ac. Active enhancing regions.	Primary B cells from peripheral blood	H3K27ac_Enh
High signal of H3K4me1 and H3K27ac. Active enhancing regions.	Lung	H3K4me1_Enh
High signal of H3K4me1 and H3K27ac. Active enhancing regions.	Monocytes-CD14+ RO01746 Primary Cells	H3K9ac_Pro
High signal of H3K4me1 and H3K27ac. Active enhancing regions.		
	Lung	H3K4me1_Enh
	Spleen	H3K4me1_Enh
	Monocytes-CD14+ RO01746 Primary Cells	H3K4me1_Enh
	Primary mononuclear cells from peripheral blood	H3K9ac_Pro
High H3K36me3 signal. Transcription elongation regions.		
High signal of H3K4me1 and H3K27ac. Active enhancing regions.		
High signal of H3K4me1 and H3K27ac. Active enhancing regions.		
H3K9me3 and/or H3K27me3 signal. Generally inactive and repetitive regions.	Monocytes-CD14+ RO01746 Primary Cells	H3K9ac_Pro
High signal of H3K4me1 and H3K27ac. Active enhancing regions.	Primary mononuclear cells from peripheral blood	H3K9ac_Pro
High signal of H3K4me1 and H3K27ac. Active enhancing regions.		
High signal of H3K4me1 and H3K27ac. Active enhancing regions.		
High signal of H3K4me1 and H3K27ac. Active enhancing regions.		
High signal of H3K4me1 and H3K27ac. Active enhancing regions.		
High signal of H3K4me1 and H3K27ac. Active enhancing regions.		
High signal of H3K4me1 and H3K27ac. Active enhancing regions.		
High signal of H3K4me1 but low H3K27ac. Less active enhancing regions.	Primary B cells from cord blood	H3K4me1_Enh
High signal of H3K4me1 but low H3K27ac. Less active enhancing regions.	Primary B cells from peripheral blood	H3K4me1_Enh and H3K27ac_Enh
High signal of H3K4me1 and H3K27ac. Active enhancing regions.	Primary T cells from peripheral blood	H3K4me1_Enh and H3K27ac_Enh
High signal of H3K4me1 but low H3K27ac. Less active enhancing regions.	Primary hematopoietic stem cells	H3K4me1_Enh
High signal of H3K4me1 but low H3K27ac. Less active enhancing regions.	Primary hematopoietic stem cells short term culture	H3K4me1_Enh and H3K4me3_Pro
High signal of H3K4me1 but low H3K27ac. Less active enhancing regions.	Primary T helper memory cells from peripheral blood 2	H3K4me1_Enh
High signal of H3K4me1 and H3K27ac. Active enhancing regions.	Primary T helper naive cells from peripheral blood	H3K4me1_Enh
High signal of H3K4me1 and H3K27ac. Active enhancing regions.	Primary T helper memory cells from peripheral blood 1	H3K4me1_Enh

Histone marks c

Consequence	Tissue	Marks
High signal of H3K4me1 but low H3K27ac. Less active enhancing regions.	Primary T helper cells PMA-I stimulated	H3K4me1_Enh
High signal of H3K4me1 and H3K27ac. Active enhancing regions.	Primary T helper 17 cells PMA-I stimulated	H3K4me1_Enh
High signal of H3K4me1 but low H3K27ac. Less active enhancing regions.	Primary T helper cells from peripheral blood	H3K4me1_Enh
High signal of H3K4me1 but low H3K27ac. Less active enhancing regions.	Primary T regulatory cells from peripheral blood	H3K4me1_Enh
	Primary hematopoietic stem cells G-CSF-mobilized Female	H3K4me1_Enh and H3K27ac_Enh
High signal of H3K4me1 and H3K27ac. Active enhancing regions.	Primary hematopoietic stem cells G-CSF-mobilized Male	H3K4me1_Enh and H3K4me3_Pro
High signal of H3K4me1 and H3K27ac. Active enhancing regions.	Primary B cells from peripheral blood	H3K4me1_Enh
High signal of H3K4me1 and H3K27ac. Active enhancing regions.	Primary T cells from cord blood	H3K4me1_Enh
High signal of H3K4me1 and H3K27ac. Active enhancing regions.	Primary T cells from peripheral blood	H3K4me1_Enh and H3K27ac_Enh
High signal of H3K4me1 and H3K27ac. Active enhancing regions.	Primary T helper memory cells from peripheral blood 2	H3K4me1_Enh and H3K27ac_Enh
High signal of H3K4me1 and H3K27ac. Active enhancing regions.	Primary T helper naive cells from peripheral blood	H3K4me1_Enh and
High signal of H3K4me1 and H3K27ac. Active enhancing regions.	Primary T helper naive cells from peripheral blood	H3K4me1_Enh and H3K27ac_Enh
High signal of H3K4me1 and H3K27ac. Active enhancing regions.	Primary T helper memory cells from peripheral blood 1	H3K4me1_Enh and
High signal of H3K4me1 and H3K27ac. Active enhancing regions.	Primary T helper cells PMA-I stimulated	H3K4me1_Enh and H3K27ac_Enh
High signal of H3K4me1 and H3K27ac. Active enhancing regions.	Primary T helper 17 cells PMA-I stimulated	H3K4me1_Enh; H3K4me3_Pro and H3K27ac_Enh
High signal of H3K4me1 and H3K27ac. Active enhancing regions.	Primary T helper cells from peripheral blood	H3K4me1_Enh and H3K27ac_Enh
High signal of H3K4me1 but low H3K27ac. Less active enhancing regions.	Primary T regulatory cells from peripheral blood	H3K4me1_Enh and H3K27ac_Enh
High signal of H3K4me1 and H3K27ac. Active enhancing regions.	Primary T cells effector/memory enriched from peripheral blood	and H3K27ac_Enh
High signal of H3K4me1 and H3K27ac. Active enhancing regions.	Primary T CD8+ naive cells from peripheral blood	H3K4me1_Enh and
High signal of H3K4me1 and H3K27ac. Active enhancing regions.	Primary T CD8+ memory cells from peripheral blood	H3K4me1_Enh and H3K27ac_Enh
High signal of H3K4me1 and H3K27ac. Active enhancing regions.	Mesenchymal Stem Cell Derived Chondrocyte Cultured Cells	H3K4me1_Enh; H3K27ac_Enh and H3K9ac_Pro
High signal of H3K4me1 and H3K27ac. Active enhancing regions.	Lung	H3K4me1_Enh
High signal of H3K4me1 and H3K27ac. Active enhancing regions.	Spleen	H3K27ac_Enh
High signal of H3K4me1 and H3K27ac. Active enhancing regions.		
High signal of H3K4me1 and H3K27ac. Active enhancing regions.	NHLF Lung Fibroblast Primary Cells	H3K4me1_Enh; H3K27ac_Enh and H3K9ac_Pro
High signal of H3K4me1 and H3K27ac. Active enhancing regions.		

Dnase c	Protein bound c		Regulatory Motifs Changed c	Taits associated d	QTL e					
	Cell ID	Protein			Tissue	Gene/Trait	Effective Allele	Pvalue	Molecular trait	PMID studie
			Bcl6b							
			CIZ							
			Gfi1_1							
			Gfi1b							
			Hdx							
			Irf_known3							
			Pou3f1							
			Pou5f1_disc1							
			STAT_disc4							
			STAT_known1							
			STAT_known2							
			Sox_4							
	NHEK	CTCF			Blood-T cell CD4+ naive	cg07225362 (chr3:9975367)	NA	0.00000666	mQTL	27863251
					Blood-T cell CD4+ naive	cg18031978 (chr3:9851572)	NA	0.000012	mQTL	27863251
					Blood-Monocytes CD14+	cg09660365 (chr3:10858395)	NA	0.0000203	mQTL	27863251
					Blood-T cell CD4+ naive	cg05833158 (chr3:9993731)	NA	0.000222	mQTL	27863251
					Blood-T cell CD4+ naive	cg10829427 (chr3:9885775)	NA	0.00031	mQTL	27863251
					Blood-T cell CD4+ naive	cg11021744 (chr3:11034279)	NA	0.000699	mQTL	27863251
					Blood-T cell CD4+ naive	cg13672843 (chr3:10183721)	NA	0.000737	mQTL	27863251
					Blood-T cell CD4+ naive	cg18083704 (chr3:9851847)	NA	0.000754	mQTL	27863251
					Blood-Monocytes CD14+	cg13704678 (chr3:10362667)	NA	0.000798	mQTL	27863251
			SETDB1_disc1		Blood	cg14223444 (chr9:100394496)	T	1.33E-25	mQTL	34493871
			Znf143_disc3							
			AP-1_disc3							
			AP-1_disc9							
			AP-1_known1							
			AP-2_disc1							
			BAF155_disc1							
			BATF_disc1							
			Bach1							
			Bach2							
			E2A_2							
			GATA_disc2							
			GR_disc2							
			HMGN3_disc1							
			KAP1_disc1							
			Myc_disc3							
			PRDM1_disc2							
			TBX5_3							
			TCF4_disc1							
			ZEB1_disc1							
			ZEB1_known4							
			p300_disc1							
			Egr-1_disc4		Blood	MAP2K5	C	0.000043	eQTL	bioRxiv_447367
			Myc_known6							
			Rad21_disc8							
			Smad_1							
			Zic_4		Blood-Monocytes CD14+	cg17184477 (chr15:67360705)	NA	0.000724	mQTL	27863251
			Znf143_disc3							
			Znf143_known1							
			AIRE_2		Blood-Monocytes CD14+	cg12271079 (chr10:117997745)	NA	0.0000885	mQTL	27863251
			CEBPB_known4							
			Foxi1							
			HNF4_disc4		Blood-T cell CD4+ naive	cg12271079 (chr10:117997745)	NA	0.000135	mQTL	27863251
			Maf_known3							
			Maf_known4							
			Pou2f2_known2		Blood-T cell CD4+ naive	cg13890706 (chr10:118033115)	NA	0.000982	mQTL	27863251
			SRF_known5							
			TCF12_disc2							
			E2A_2		Blood	cg03015368 (chr16:10065222)	NA	4.42E-08	mQTL	27036880
			E2A_5		Blood-Monocytes CD14+	cg03015368 (chr16:10065222)	NA	0.0000825	mQTL	27863251
					Blood-T cell CD4+ naive	cg09001143 (chr16:9857475)	NA	0.000619	mQTL	27863251
			E2A_2		Lung	SNX24	A	0.00000963	eQTL	25954001
					Lung	SNX24	A	0.0000135	eQTL	32913098
					Spleen	SNX24	A	0.000113	eQTL	32913098
					Blood	cg04058593 (chr5:122425084)	A	0.00026	mQTL	30871624
			Eomes	Cysteine-rich with EGF-like domain protein 1 levels (PMID: 36168886)	Blood	cg04230438 (chr5:672357)	NA	9.78E-08	mQTL	27036880
			SRF_known5							
			ZEB1_known4							
			Gfi1_1		Lymphocyte	SPTBN1	C	0.0000826	eQTL	25951796
					Blood	cg26097391 (chr2:54893211)	NA	7.47E-09	mQTL	27036880
					Blood-Monocytes CD14+	cg26097391 (chr2:54893211)	NA	0.00000247	mQTL	27863251
					Blood-T cell CD4+ naive	cg26097391 (chr2:54893211)	NA	0.0000117	mQTL	27863251
					Blood-Monocytes CD14+	cg02717117 (chr2:55458694)	NA	0.0000793	mQTL	27863251
					Blood-T cell CD4+ naive	cg23072029 (chr2:55746572)	NA	0.000715	mQTL	27863251
					Blood-T cell CD4+ naive	cg05084827 (chr2:55402999)	NA	0.000937	mQTL	27863251
CCNT2_disc1					Blood	CYBRD1	A	3.29E-09	eQTL	24013639
					Blood	CYBRD1	A	5.82E-07	eQTL	32913098
GATA_known1										
					Blood	CYBRD1	A	0.00000268	eQTL	25954001
GATA_known14					Blood	cg12072024 (chr2:172377981)	NA	3.3E-08	mQTL	27036880

Dnase c	Protein bound c		Regulatory Motifs Changed c	Taits associated d	QTL e					
	Cell ID	Protein			Tissue	Gene/Trait	Effective Allele	Pvalue	Molecular trait	PMID studie
					Blood	cg24200501 (chr2:172378036)	NA	6.41E-08	mQTL	27036880
GATA_known2										
TAL1_disc1					Blood-T cell CD4+ naive	cg18183163 (chr2:171574141)	NA	0.000758	mQTL	27863251
			Osr							
			SMC3_disc2							
			SMC3_disc4							
			CDP_2							
			CDP_3							
			Cart1							
			Foxa_known4							
			Foxd3							
			Foxf1							
			Foxf2							
			Foxi1							
			Foxj1_1							
			Foxj1_2							
			Foxj2_1							
			Foxk1							
			Foxl1_1							
			Foxo_1							
			Foxp1							
			Foxq1							
			HNF1_6							
			Hmx_2							
			Ncx_2							
			Pbx-1_1							
			Sox_18							
