# Supplementary material for: A genome-wide association study identifies new loci associated with response to SARS-CoV-2 mRNA-1273 vaccine in a cohort of healthy healthcare workers
Source: Front Immunol. 2025 Aug 18;16:1639825. doi: 10.3389/fimmu.2025.1639825 (PMC12409172; doi:10.3389/fimmu.2025.1639825)
Supplement: Supplementary file 9 [file DataSheet9.pdf]

Supplementary Table 5. Potential genome-wide association with Antibodies production identified in GWASs.

	SNP	Chr	Position	Alt/Ref	Nearest gene(s)	Consequence	MAF (1,000 Genomes)	MAF (Cohort)	Beta	Standard error	P_{value}
GWAS 1	rs12573939	11	127858117	A/G	*ENSG00000307057*	lncRNA	0.082	0.080	0.634	0.116	$7.76×10^{-08}$
	rs72724944	1	173249164	T/C	*TNFSF4 | LOC100506023*	Upstream gene variant | Intron Variant	0.056	0.090	0.653	0.121	$1.29×10^{-07}$
	rs72857564	18	311288	A/G	*THOC1 | COLEC12*	Intergenic variant	0.073	0.088	0.566	0.105	$1.31×10^{-07}$
	rs1125991	2	172263448	A/T	*METTL8*	Intron variant	0.072	0.083	0.587	0.107	$7.93×10^{-08}$
	rs72943937	1	84833879	A/G	*UOX*	Intron variant	0.054	0.067	0.635	0.119	$1.76×10^{-07}$
	rs117613634	18	74157596	A/G	*ZNF516*	Intron variant	0.075	0.063	0.720	0.136	$1.96×10^{-07}$
	rs55919500	20	8072501	A/G	*PLCB1*	Intron variant	0.101	0.065	0.683	0.129	$2.12×10^{-07}$
	rs4630616	17	76330484	A/G	*SOCS3*	Intron variant	0.176	0.153	0.521	0.099	$2.28×10^{-07}$
	rs147925372	21	29726142	T/C	*ENSG00000232855 | ENSG00000307297*	lncRNAs	0.068	0.059	0.643	0.122	$2.46×10^{-07}$
	rs12985705	19	13582682	G/A	*CACNA1A*	Intron variant	0.090	0.077	0.633	0.120	$2.48×10^{-07}$
	rs10859690	12	94638309	A/G	*PLXNC1*	Intron variant	0.175	0.166	0.457	0.087	$2.64×10^{-07}$
	rs61223015	21	36523905	A/G	*ENSG00000234703 | RUNX1*	Intergenic variant	0.052	0.077	0.636	0.122	$2.84×10^{-07}$
	rs12258039	10	29449253	A/G	*LYZL1*	Regulatory region variant	0.084	0.099	0.522	0.100	$3.04×10^{-07}$
	rs17127526	14	54857961	A/G	*ENSG00000300202*	lncRNA	0.061	0.055	0.704	0.135	$3.31×10^{-07}$
	rs11608994	12	30168134	C/T	*LOC105369715*	Intron variant	0.095	0.101	0.551	0.106	$3.84×10^{-07}$
	rs113707319	9	106215609	T/C	*ENSG00000226566 | ENSG00000307505*	Intergenic variant	0.067	0.071	0.618	0.120	$4.28×10^{-07}$
	rs12699442	7	13147602	T/G	*ENSG00000229618*	lncRNA	0.096	0.071	0.671	0.131	$4.84×10^{-07}$
	rs8178712	8	42054929	G/A	*PLAT*	Intron variant	0.077	0.090	0.536	0.105	$4.91×10^{-07}$
	rs12329849	21	17012801	C/G	*RNU6-1326P | USP25*	Intergenic variant	0.075	0.088	0.639	0.125	$5.41×10^{-07}$
GWAS 2	rs2559605	12	106593284	G/T	*NUAK1 | CKAP4*	Intergenic variant	0.176	0.120	0.498	0.095	$1.58×10^{-07}$
	rs563885	1	77070851	C/A	*ST6GALNAC3*	Intron variant	0.455	0.498	-0.352	0.067	$1.78×10^{-07}$
	rs6651358	8	39991168	A/T	*LOC105379385*	Intron variant	0.082	0.060	0.705	0.136	$2.21×10^{-07}$
	rs12425019	12	13063131	G/T	*GPRC5A*	Intron variant	0.292	0.264	0.381	0.074	$2.40×10^{-07}$
	rs9287357	2	144799194	C/G	*GTDC1*	Intron variant	0.042	0.051	0.760	0.147	$2.61×10^{-07}$
	rs12206038	6	140055651	A/G	*FILNC1*	Intron variant	0.064	0.047	0.713	0.140	$3.96×10^{-07}$
	rs2290168	14	81009746	A/C	*CEP128*	Intron variant	0.145	0.113	0.543	0.110	$8.14×10^{-07}$
	rs4304398	9	118376727	T/C	*LOC105376235*	Intron variant	0.069	0.110	0.749	0.152	$8.48×10^{-07}$
	rs77458347	4	109896081	T/C	*COL25A1*	Intron variant	0.103	0.103	0.638	0.130	$9.58×10^{-07}$
GWAS 3	rs72857564	18	311288	A/G	*THOC1 | COLEC12*	Intergenic variant	0.073	0.088	0.652	0.115	$5.10×10^{-08}$
	rs55636630	1	81394192	A/C	*ENSG00000301718 | ENSG00000295563*	Intergenic variant	0.066	0.062	0.793	0.141	$5.16×10^{-08}$
	rs28485994	15	67257395	C/T	*SMASR | SMAD3-DT*	Intergenic variant	0.094	0.097	0.626	0.113	$9.76×10^{-08}$
	rs35404194	5	137847082	C/T	*ETF1*	Intergenic variant	0.045	0.099	0.723	0.131	$9.92×10^{-08}$
	rs72724944	1	173249164	T/C	*TNFSF4 | LOC100506023*	Upstream gene variant | Intron Variant	0.056	0.090	0.737	0.134	$1.14×10^{-07}$

rs17234651	11	23928672	A/G	*lnc-LUZP2-3 | HSALNG0143151*	Intergenic variant	0.072	0.068	0.799	0.146	1.23×10^{-07}
rs114863311	12	115004484	C/T	*RN7SKP216 | ENSG00000258254*	Intergenic variant	0.056	0.063	0.801	0.149	2.20×10^{-07}
rs9397844	6	156137175	C/T	*LOC105378072 | LOC101928923*	Intron variant	0.072	0.073	0.800	0.150	2.92×10^{-07}
rs9384447	6	156575128	A/G	*LOC101928923*	Intron variant	0.080	0.053	0.759	0.143	3.23×10^{-07}
rs10777797	12	96856310	T/C	*ENSG00000258272*	lncRNA	0.065	0.063	0.739	0.139	3.23×10^{-07}
rs77038260	4	182592048	A/G	*ENSG00000299420*	lncRNA	0.089	0.065	0.548	0.104	3.35×10^{-07}
rs12573939	11	127858117	A/G	*ENSG00000307057*	lncRNA	0.082	0.080	0.662	0.125	3.53×10^{-07}
rs1226147	9	104146218	T/C	*BAAT*	Intron variant	0.075	0.068	0.750	0.143	4.13×10^{-07}
rs116993946	7	42523957	G/A	*ENSG00000294440 | ENSG00000308043*	Intergenic variant	0.030	0.044	0.777	0.148	4.34×10^{-07}
rs55908585	3	15651778	A/C	*BTD*	Intron variant	0.081	0.065	0.740	0.141	4.39×10^{-07}
rs2302628	12	96387765	A/T	*HAL*	Synonymous Variant	0.077	0.051	0.764	0.146	4.50×10^{-07}
rs12543123	8	41985163	T/C	*ENSG00000309019 | MYL9*	Intergenic variant	0.056	0.065	0.721	0.138	5.00×10^{-07}
rs72832295	2	105782546	A/G	*LINC01918 | ENSG00000293860*	Intergenic variant	0.094	0.075	0.689	0.132	5.13×10^{-07}
rs34402473	17	36173120	T/C	*LOC105371757*	Intron variant	0.268	0.174	0.493	0.095	6.39×10^{-07}
rs17127526	14	54857961	A/G	*ENSG00000300202*	lncRNA	0.061	0.055	0.778	0.151	6.52×10^{-07}
rs4688637	3	61575424	C/A	*PTPRG*	Intron variant	0.103	0.092	0.753	0.146	6.82×10^{-07}
rs75748959	1	112061174	T/G	*TMIGD3*	Intron variant	0.060	0.063	0.697	0.135	7.06×10^{-07}
rs4690312	4	768848	G/C	*PCGF3-AS1*	Intron variant	0.069	0.130	0.774	0.150	7.06×10^{-07}
rs189336	13	92333539	C/A	*GPC5*	Intron variant	0.054	0.056	0.751	0.146	7.23×10^{-07}
rs76459611	11	42415665	C/T	*LOC105376642 | HNRNPKP3*	Intergenic variant	0.132	0.136	0.520	0.102	7.91×10^{-07}
rs4088054	3	10808633	T/A	*LINC00606 | ENSG00000230599*	Intergenic variant	0.088	0.093	0.621	0.122	9.12×10^{-07}
rs56298314	3	15613279	G/T	*HACL1*	Intron variant	0.053	0.072	0.697	0.137	9.21×10^{-07}
rs780004	2	125146301	C/T	*CNTNAP5*	Intron variant	0.115	0.143	0.490	0.096	9.87×10^{-07}

Abbreviations: SNP, single nucleotide polymorphism; Chr, Chromosome; Alt, alternative allele; Ref, reference allele; MAF, minor allele frequency; OR, odds ratio; CI, confidence interval; P, P-value; Phet, P-value of heterogeneity.

GWAS 1: The GWAS analysis of IgG levels measured at the first month after mRNA-1273 vaccination.

GWAS 2: The GWAS analysis of IgG levels measured at the third month after mRNA-1273 vaccination.

GWAS 3: The GWAS analysis of the difference in IgG levels between the first and third months after mRNA-1273 vaccination.

GWAS analyses were conducted using linear regression with PLINK software. Estimates calculated according to a log-additive model of inheritance and adjusted for age, sex and 10 first principal components.
