# Supplementary material for: A genome-wide association study identifies new loci associated with response to SARS-CoV-2 mRNA-1273 vaccine in a cohort of healthy healthcare workers
Source: Front Immunol. 2025 Aug 18;16:1639825. doi: 10.3389/fimmu.2025.1639825 (PMC12409172; doi:10.3389/fimmu.2025.1639825)
Supplement: Supplementary file 10 [file DataSheet10.pdf]

Supplementary Table 3. Effect of age and sex on IgG levels and IgG decay over time.

1 Month post-vaccination

		IgG Levels			Age		
	Obs	Median	IQR	Pvalue	Median	IQR	Pvalue
**Overall**	567	2424.31	1543.41-3891.87		50	40-58	
**Men**	201	2580.99	1686.02-4138.58	0.025	48	39-56	0.021
**Women**	366	2374.86	1482.44-3753.00		52	40.25-59	

Patient	IgG	Age	Sex	IgG_Z-score
CV_004	8975.99	52	1	1.9017
CV_005	5178.5	40	1	0.6919
CV_012	1651.55	27	1	-0.4318
CV_013	2216.32	48	1	-0.2519
CV_015	1583.87	54	1	-0.4534
CV_020	3445.83	35	1	0.1399
CV_021	5204.1	42	1	0.7000
CV_022	4693.48	61	1	0.5373
CV_024	2340.03	33	1	-0.2124
CV_026	1934.89	64	1	-0.3415
CV_029	5682	50	1	0.8523
CV_036	1387.568	65	1	-0.5159
CV_037	2325.93	39	1	-0.2169
CV_039	4558.34	57	1	0.4943
CV_041	2747.67	62	1	-0.0826
CV_042	3312.01	56	1	0.0972
CV_045	2760.64	40	1	-0.0784
CV_048	1812.83	42	1	-0.3804
CV_049	5304.28	64	1	0.7319
CV_052	2283.67	50	1	-0.2304
CV_059	5682	29	1	0.8523
CV_061	1992.6	48	1	-0.3231
CV_067	4421.87	64	1	0.4508
CV_069	1324.79	50	1	-0.5359
CV_070	2853.99	55	1	-0.0487
CV_073	1091.14	58	1	-0.6103
CV_074	2356.28	34	1	-0.2073
CV_076	3469.97	57	1	0.1475
CV_080	4000.34	50	1	0.3165
CV_084	740.46	56	1	-0.7221
CV_086	778.9	39	1	-0.7098
CV_087	1011.57	45	1	-0.6357
CV_090	2537.3	36	1	-0.1496
CV_092	3597.17	44	1	0.1881
CV_094	1395.04	25	1	-0.5135
CV_095	2076.93	29	1	-0.2963
CV_096	2270.32	59	1	-0.2347
CV_099	1006.75	55	1	-0.6372
CV_101	3304.6	53	1	0.0949
CV_104	3993.52	30	1	0.3143
CV_109	4734.01	46	1	0.5503
CV_110	2094.37	49	1	-0.2907
CV_111	3885.83	31	1	0.2800
CV_129	2067.89	64	1	-0.2992
CV_138	2103.43	40	1	-0.2878
CV_141	1539.34	38	1	-0.4675
CV_144	3041.81	52	1	0.0111
CV_148	1344.85	40	1	-0.5295
CV_150	1813.11	42	1	-0.3803
CV_151	1106.82	53	1	-0.6053
CV_153	3059.67	44	1	0.0168
CV_159	1446.58	45	1	-0.4971
CV_164	37360	41	1	10.9447
CV_167	4138.58	26	1	0.3606
CV_170	2187.42	62	1	-0.2611
CV_171	2734.31	62	1	-0.0868
CV_175	1086.85	59	1	-0.6117
CV_177	1015.63	28	1	-0.6344
CV_182	1301.5	33	1	-0.5433
CV_185	2476.48	43	1	-0.1690
CV_186	2424.24	52	1	-0.1856
CV_189	1962.77	59	1	-0.3326
CV_190	3185.09	47	1	0.0568
CV_194	3676.49	48	1	0.2133
CV_197	3962.64	56	1	0.3045
CV_198	7876.94	44	1	1.5516
CV_199	1715.77	60	1	-0.4113
CV_200	1942.08	59	1	-0.3392
CV_201	1574.07	50	1	-0.4565
CV_212	2682.39	44	1	-0.1034
CV_214	2265.88	43	1	-0.2361
CV_215	1195.85	53	1	-0.5770
CV_219	4151.21	30	1	0.3646
CV_223	4458.87	59	1	0.4626
CV_228	4176.93	44	1	0.3728
CV_240	1761.81	32	1	-0.3967
CV_241	2150.39	41	1	-0.2729
CV_246	3101.54	45	1	0.0302
CV_266	1870.69	38	1	-0.3620
CV_276	4126.48	56	1	0.3567
CV_299	2133.66	54	1	-0.2782
CV_302	38000	62	1	11.1486
CV_305	5294.81	53	1	0.7289

CV_307	7305.73	53	1	1.3696
CV_309	3913.52	46	1	0.2889
CV_311	1162.24	50	1	-0.5877
CV_313	4855.07	53	1	0.5888
CV_315	4817.41	27	1	0.5768
CV_318	3435.14	44	1	0.1364
CV_319	2169.29	49	1	-0.2668
CV_322	2773.67	49	1	-0.0743
CV_324	3684.7	38	1	0.2160
CV_326	1136.14	57	1	-0.5960
CV_327	3684.05	43	1	0.2157
CV_330	2960.69	52	1	-0.0147
CV_331	2530.67	50	1	-0.1517
CV_336	1340.25	36	1	-0.5310
CV_337	2811.05	61	1	-0.0624
CV_340	4300.09	37	1	0.4120
CV_341	6601.01	51	1	1.1451
CV_344	4065.32	37	1	0.3372
CV_345	3031.8	46	1	0.0079
CV_348	3378.11	24	1	0.1183
CV_353	1686.02	63	1	-0.4208
CV_354	5682	55	1	0.8523
CV_355	4128.12	60	1	0.3572
CV_358	5682	28	1	0.8523
CV_360	4061.56	33	1	0.3360
CV_361	1984.42	50	1	-0.3257
CV_366	2750.7	36	1	-0.0816
CV_369	1789.47	54	1	-0.3879
CV_371	2252.32	63	1	-0.2404
CV_372	4795.16	41	1	0.5697
CV_373	2413.67	56	1	-0.1890
CV_376	2097.92	60	1	-0.2896
CV_377	1861.04	61	1	-0.3651
CV_378	41360	63	1	12.2191
CV_380	1929.75	44	1	-0.3432
CV_382	1847.06	29	1	-0.3695
CV_383	1697.64	60	1	-0.4171
CV_387	3838.29	44	1	0.2649
CV_391	3468.39	60	1	0.1470
CV_393	2046.7	32	1	-0.3059
CV_396	4150.38	60	1	0.3643
CV_398	2790.27	60	1	-0.0690
CV_400	2795.68	25	1	-0.0673
CV_401	4900.15	30	1	0.6032
CV_405	1639.93	32	1	-0.4355
CV_407	4444.07	53	1	0.4579
CV_411	1227.96	63	1	-0.5667
CV_412	4736.48	60	1	0.5510
CV_415	2150.56	43	1	-0.2728
CV_419	2249.12	39	1	-0.2414
CV_424	3832.92	24	1	0.2632
CV_426	5401.69	54	1	0.7630
CV_432	3208.52	30	1	0.0642
CV_434	2529.52	43	1	-0.1521
CV_436	2275.72	23	1	-0.2329
CV_437	935.91	48	1	-0.6598
CV_439	762.75	63	1	-0.7150
CV_440	1545.5	52	1	-0.4656
CV_442	3237.5	41	1	0.0735
CV_448	4183.14	33	1	0.3748
CV_452	2713.19	48	1	-0.0936
CV_453	2749.94	40	1	-0.0819
CV_455	1549.04	65	1	-0.4645
CV_461	7283.95	57	1	1.3627
CV_466	5291.18	39	1	0.7278
CV_471	3172.07	49	1	0.0526
CV_477	2503.33	33	1	-0.1604
CV_481	2232.28	34	1	-0.2468
CV_490	2289.81	63	1	-0.2284
CV_492	1600.85	42	1	-0.4479
CV_495	3792.95	34	1	0.2504
CV_496	3008.6	57	1	0.0006
CV_497	2004.23	33	1	-0.3194
CV_498	1528.74	29	1	-0.4709
CV_499	1338.7	63	1	-0.5315
CV_500	2837.67	49	1	-0.0539
CV_503	1431.35	51	1	-0.5019
CV_505	382.43	64	1	-0.8361
CV_506	1107.73	31	1	-0.6051
CV_507	2381.94	55	1	-0.1991
CV_508	5682	26	1	0.8523
CV_517	6789.734	65	1	1.2052
CV_523	1545.404	62	1	-0.4656
CV_525	4014.318	53	1	0.3210
CV_526	4226.796	29	1	0.3887
CV_527	1300.138	63	1	-0.5438
CV_528	5682	44	1	0.8523
CV_529	1580.8	57	1	-0.4543

CV_532	65.48	63	1	-0.9371
CV_533	680.25	52	1	-0.7412
CV_534	1365.9	46	1	-0.5228
CV_535	9854.5	23	1	2.1816
CV_537	446.79	33	1	-0.8156
CV_541	550.29	44	1	-0.7826
CV_542	5682	43	1	0.8523
CV_543	609.27	27	1	-0.7639
CV_544	6368.56	45	1	1.0710
CV_545	5682	38	1	0.8523
CV_546	871.33	41	1	-0.6804
CV_548	5208.03	34	1	0.7013
CV_549	490.24	46	1	-0.8018
CV_550	870.55	45	1	-0.6806
CV_554	863.53	50	1	-0.6829
CV_567	4625.47	50	1	0.5157
CV_568	6764.67	43	1	1.1972
CV_569	718.79	60	1	-0.7290
CV_570	2212.62	51	1	-0.2530
CV_571	404.47	52	1	-0.8291
CV_572	829.24	27	1	-0.6938
CV_573	10137.12	64	1	2.2717
CV_574	8769.03	51	1	1.8358
CV_575	3982.11	52	1	0.3107
CV_577	6727.29	51	1	1.1853
CV_579	1843.87	49	1	-0.3705
CV_580	2580.99	53	1	-0.1357
CV_581	5244.13	42	1	0.7128
CV_582	3650.04	51	1	0.2049
CV_583	5682	55	1	0.8523
CV_001	5102.36	23	2	0.6676
CV_002	1736.96	46	2	-0.4046
CV_003	4872.94	56	2	0.5945
CV_006	4910.06	45	2	0.6063
CV_007	871.84	27	2	-0.6802
CV_008	1855.76	40	2	-0.3667
CV_009	1920	33	2	-0.3463
CV_010	2007.07	39	2	-0.3185
CV_011	4790.71	61	2	0.5683
CV_014	1590.83	61	2	-0.4511
CV_016	1842.14	50	2	-0.3711
CV_017	1642.14	39	2	-0.4348
CV_018	951.03	55	2	-0.6550
CV_019	1897.74	51	2	-0.3534
CV_025	2236.67	63	2	-0.2454
CV_027	953.69	46	2	-0.6541
CV_028	3922.61	44	2	0.2918
CV_031	794.52	33	2	-0.7048
CV_032	2103.374	62	2	-0.2878
CV_033	3760.05	36	2	0.2400
CV_034	4186.182	26	2	0.3757
CV_035	4782.624	63	2	0.5657
CV_038	1339.63	51	2	-0.5312
CV_040	4604.62	54	2	0.5090
CV_043	2049.4	37	2	-0.3050
CV_044	2087.58	32	2	-0.2929
CV_046	3162.33	60	2	0.0495
CV_047	1803.54	42	2	-0.3834
CV_050	1356.14	39	2	-0.5259
CV_051	2283.46	34	2	-0.2305
CV_053	2921.25	53	2	-0.0273
CV_054	577.64	56	2	-0.7739
CV_055	4192.59	45	2	0.3778
CV_056	729.68	52	2	-0.7255
CV_058	1472.7	65	2	-0.4888
CV_060	1365.83	57	2	-0.5228
CV_062	1208.68	33	2	-0.5729
CV_063	1075.76	39	2	-0.6152
CV_064	1625.18	60	2	-0.4402
CV_065	4277.92	60	2	0.4050
CV_066	5682	58	2	0.8523
CV_068	1445.57	56	2	-0.4974
CV_071	4953.54	52	2	0.6202
CV_072	3118.11	39	2	0.0354
CV_075	4436.69	53	2	0.4555
CV_077	2413.72	65	2	-0.1890
CV_078	2670.38	25	2	-0.1072
CV_079	2837.12	51	2	-0.0541
CV_081	5445.59	57	2	0.7770
CV_082	2023.27	49	2	-0.3134
CV_083	2888.96	48	2	-0.0376
CV_085	3026.19	64	2	0.0062
CV_088	2065.63	49	2	-0.2999
CV_089	1595.04	56	2	-0.4498
CV_091	1196.04	44	2	-0.5769
CV_093	1675.44	59	2	-0.4242
CV_097	4065.09	48	2	0.3371
CV_098	937.63	45	2	-0.6592

CV_100	892.56	52	2	-0.6736
CV_103	4081.99	44	2	0.3425
CV_105	2481.58	58	2	-0.1674
CV_106	3770.97	41	2	0.2434
CV_107	2857.92	59	2	-0.0475
CV_108	1030.21	58	2	-0.6297
CV_112	2460.68	55	2	-0.1740
CV_113	1415.56	59	2	-0.5070
CV_114	2853.48	62	2	-0.0489
CV_115	3658.84	59	2	0.2077
CV_116	1684.99	48	2	-0.4211
CV_117	2842.63	51	2	-0.0523
CV_118	4510.26	56	2	0.4790
CV_119	1259.58	45	2	-0.5567
CV_120	1376.51	55	2	-0.5194
CV_121	1548.92	59	2	-0.4645
CV_122	2121.47	52	2	-0.2821
CV_123	5443.71	44	2	0.7764
CV_124	3467.36	24	2	0.1467
CV_125	3751.28	61	2	0.2372
CV_126	2429.19	36	2	-0.1840
CV_127	2270.2	35	2	-0.2347
CV_128	1506.31	52	2	-0.4781
CV_130	3838.77	45	2	0.2650
CV_131	2434.94	43	2	-0.1822
CV_132	1067.97	60	2	-0.6177
CV_133	1044.07	54	2	-0.6253
CV_134	506.34	36	2	-0.7967
CV_135	1456.15	30	2	-0.4940
CV_136	4315.96	55	2	0.4171
CV_137	1074.91	57	2	-0.6155
CV_139	1719.71	54	2	-0.4101
CV_140	2836	48	2	-0.0544
CV_142	2941.73	41	2	-0.0208
CV_143	1477.6	52	2	-0.4872
CV_145	1418.62	64	2	-0.5060
CV_146	1520.91	33	2	-0.4734
CV_152	1099.05	62	2	-0.6078
CV_154	2166.12	40	2	-0.2679
CV_155	2213.2	61	2	-0.2529
CV_156	2491.06	51	2	-0.1643
CV_157	1600.37	25	2	-0.4481
CV_158	2120.54	42	2	-0.2824
CV_160	1859.14	56	2	-0.3657
CV_161	1480.59	53	2	-0.4863
CV_162	2439.23	44	2	-0.1808
CV_163	3052.39	62	2	0.0145
CV_165	1074.24	57	2	-0.6157
CV_166	2508.49	54	2	-0.1588
CV_168	2552.17	25	2	-0.1449
CV_169	113.54	61	2	-0.9218
CV_172	2488.64	54	2	-0.1651
CV_173	2186.32	25	2	-0.2614
CV_174	1024.33	59	2	-0.6316
CV_176	3660.67	58	2	0.2083
CV_178	2557.92	41	2	-0.1430
CV_179	1459.08	34	2	-0.4931
CV_180	820.82	52	2	-0.6965
CV_181	3880.75	50	2	0.2784
CV_183	2432.56	50	2	-0.1830
CV_184	950.16	58	2	-0.6553
CV_187	1590.09	47	2	-0.4514
CV_188	1944.14	47	2	-0.3386
CV_191	5041.23	59	2	0.6481
CV_192	1655.88	48	2	-0.4304
CV_193	1544.07	64	2	-0.4660
CV_195	3732.92	59	2	0.2313
CV_196	1092.12	43	2	-0.6100
CV_202	3369.52	55	2	0.1155
CV_203	1617.47	49	2	-0.4427
CV_204	3753.57	35	2	0.2379
CV_205	2170.53	30	2	-0.2665
CV_206	2907.42	58	2	-0.0317
CV_207	1510.27	54	2	-0.4768
CV_208	5593.76	48	2	0.8242
CV_209	2435.97	49	2	-0.1819
CV_210	965.43	60	2	-0.6504
CV_211	3648.63	50	2	0.2045
CV_213	3647.03	54	2	0.2040
CV_216	1383.08	33	2	-0.5173
CV_217	1035.49	63	2	-0.6281
CV_218	1422.75	54	2	-0.5047
CV_220	3725.84	23	2	0.2291
CV_221	3207.51	37	2	0.0639
CV_222	1472.11	49	2	-0.4890
CV_224	506.16	38	2	-0.7967
CV_225	1397.73	49	2	-0.5127
CV_226	1253.5	55	2	-0.5586

CV_227	3200	59	2	0.0615
CV_229	1809.59	58	2	-0.3814
CV_230	1955.92	38	2	-0.3348
CV_231	1906.59	62	2	-0.3505
CV_232	1297.23	28	2	-0.5447
CV_233	746.37	40	2	-0.7202
CV_234	1700.86	35	2	-0.4161
CV_235	540.78	33	2	-0.7857
CV_236	2481.41	63	2	-0.1674
CV_237	4909	49	2	0.6060
CV_238	1467.04	40	2	-0.4906
CV_239	1542.76	62	2	-0.4665
CV_242	10705.52	64	2	2.4528
CV_244	2208.8	61	2	-0.2543
CV_245	5270.02	58	2	0.7210
CV_247	2268.05	44	2	-0.2354
CV_248	974.82	48	2	-0.6474
CV_249	4943.67	61	2	0.6171
CV_250	2510.23	61	2	-0.1582
CV_251	3474.6	60	2	0.1490
CV_252	2424.31	62	2	-0.1856
CV_253	1091.84	44	2	-0.6101
CV_254	1011.05	39	2	-0.6359
CV_255	1470.04	53	2	-0.4896
CV_256	2876.41	52	2	-0.0416
CV_257	1810.98	62	2	-0.3810
CV_258	3923.09	55	2	0.2919
CV_259	2605.46	44	2	-0.1279
CV_260	1634.36	57	2	-0.4373
CV_261	4034.35	23	2	0.3274
CV_262	2349.45	37	2	-0.2094
CV_263	524.49	47	2	-0.7909
CV_264	1678.61	21	2	-0.4232
CV_265	1497.18	63	2	-0.4810
CV_267	1648.8	22	2	-0.4327
CV_268	3907.36	59	2	0.2869
CV_269	3092.26	53	2	0.0272
CV_270	2901.8	59	2	-0.0335
CV_271	974.5	36	2	-0.6475
CV_272	2161.61	35	2	-0.2693
CV_273	1100.39	51	2	-0.6074
CV_274	1248.79	62	2	-0.5601
CV_275	4210.43	57	2	0.3835
CV_277	4573.93	42	2	0.4993
CV_278	2447.13	39	2	-0.1783
CV_280	5633.78	49	2	0.8369
CV_281	2782.36	62	2	-0.0715
CV_282	937.46	55	2	-0.6593
CV_283	2313.11	60	2	-0.2210
CV_284	884.63	23	2	-0.6761
CV_285	1704.81	61	2	-0.4148
CV_286	2409.38	48	2	-0.1904
CV_287	2295.98	52	2	-0.2265
CV_288	1563.41	53	2	-0.4599
CV_289	959.47	26	2	-0.6523
CV_290	2951.65	57	2	-0.0176
CV_291	4091.19	29	2	0.3455
CV_292	2974.89	58	2	-0.0102
CV_293	1518.21	58	2	-0.4743
CV_294	1142.62	64	2	-0.5939
CV_295	3905.09	54	2	0.2862
CV_296	2208.37	28	2	-0.2544
CV_297	2675.24	57	2	-0.1057
CV_298	2931.01	52	2	-0.0242
CV_300	2565.23	62	2	-0.1407
CV_301	1845.38	24	2	-0.3700
CV_303	3878.03	61	2	0.2776
CV_304	1180.08	36	2	-0.5820
CV_306	3897.91	35	2	0.2839
CV_308	4721.67	58	2	0.5463
CV_310	1409.97	47	2	-0.5088
CV_312	5682	59	2	0.8523
CV_314	2978.42	58	2	-0.0091
CV_316	575.01	59	2	-0.7748
CV_317	4998.67	48	2	0.6346
CV_320	5682	62	2	0.8523
CV_321	1366.93	41	2	-0.5225
CV_323	5119.57	61	2	0.6731
CV_325	2842.58	55	2	-0.0523
CV_328	1731.51	27	2	-0.4063
CV_329	2195.33	65	2	-0.2585
CV_332	7240.3	53	2	1.3488
CV_333	8166.93	60	2	1.6440
CV_334	4007.82	48	2	0.3189
CV_335	2116.99	37	2	-0.2835
CV_338	3366.22	58	2	0.1145
CV_339	4665.88	38	2	0.5286
CV_342	1023	25	2	-0.6320

CV_343	5300.14	49	2	0.7306
CV_346	2155.23	52	2	-0.2713
CV_347	2417.68	54	2	-0.1877
CV_349	1487.99	22	2	-0.4839
CV_350	1685.64	48	2	-0.4209
CV_351	2677.95	44	2	-0.1048
CV_352	1838.6	26	2	-0.3722
CV_356	3703.8	58	2	0.2220
CV_359	246.61	59	2	-0.8794
CV_362	2906.36	37	2	-0.0320
CV_363	3435.32	27	2	0.1365
CV_364	3880.08	38	2	0.2782
CV_365	4967.47	50	2	0.6246
CV_368	3832.78	43	2	0.2631
CV_374	1061.62	47	2	-0.6197
CV_375	1831.62	54	2	-0.3744
CV_379	4459.51	38	2	0.4628
CV_381	1449.41	36	2	-0.4962
CV_384	2260.3	53	2	-0.2378
CV_385	2571.88	30	2	-0.1386
CV_388	1356.71	47	2	-0.5257
CV_389	3957.61	56	2	0.3029
CV_390	2197.82	50	2	-0.2578
CV_392	6862.83	64	2	1.2285
CV_394	4055.78	56	2	0.3342
CV_395	2576.11	35	2	-0.1372
CV_397	4146.16	59	2	0.3630
CV_399	4621.73	55	2	0.5145
CV_402	1235.76	52	2	-0.5643
CV_403	3803.56	39	2	0.2538
CV_404	1970.7	35	2	-0.3301
CV_406	3906.83	60	2	0.2867
CV_408	4688.39	64	2	0.5357
CV_409	5538	63	2	0.8064
CV_410	2482.69	37	2	-0.1670
CV_413	3345.89	47	2	0.1080
CV_414	2364.43	53	2	-0.2047
CV_416	2577.57	60	2	-0.1368
CV_417	2560.15	54	2	-0.1423
CV_418	3746.19	46	2	0.2355
CV_420	1641.29	33	2	-0.4351
CV_422	1602.82	40	2	-0.4473
CV_423	1194.87	34	2	-0.5773
CV_425	3678.37	63	2	0.2139
CV_427	3695.69	63	2	0.2195
CV_428	4414.03	41	2	0.4483
CV_429	1028.42	60	2	-0.6303
CV_430	6659.33	55	2	1.1637
CV_433	3502.49	42	2	0.1579
CV_435	2622.16	60	2	-0.1226
CV_438	2752.77	42	2	-0.0810
CV_441	4673.58	59	2	0.5310
CV_443	1132.48	43	2	-0.5972
CV_444	1636.38	53	2	-0.4366
CV_445	2782.31	56	2	-0.0715
CV_446	2212.25	29	2	-0.2532
CV_447	4121.32	51	2	0.3551
CV_449	4216.25	55	2	0.3853
CV_450	6676.09	57	2	1.1690
CV_451	4155.98	60	2	0.3661
CV_454	1430.35	58	2	-0.5023
CV_456	9111.9	61	2	1.9450
CV_457	2385.3	58	2	-0.1980
CV_458	2934.29	33	2	-0.0231
CV_459	2951.43	64	2	-0.0177
CV_460	1544.25	42	2	-0.4660
CV_462	1314.82	63	2	-0.5391
CV_463	5375	62	2	0.7545
CV_464	1037.69	60	2	-0.6274
CV_465	1902.33	64	2	-0.3519
CV_467	2785.93	54	2	-0.0704
CV_468	3355.47	58	2	0.1111
CV_469	952.35	56	2	-0.6546
CV_470	3778.15	27	2	0.2457
CV_472	1527.83	63	2	-0.4712
CV_473	3387.57	60	2	0.1213
CV_474	3867.21	23	2	0.2741
CV_475	1524.41	53	2	-0.4723
CV_476	2417.9	56	2	-0.1876
CV_479	2711.94	56	2	-0.0940
CV_480	2546.56	58	2	-0.1466
CV_482	2800.74	40	2	-0.0657
CV_483	4144.72	61	2	0.3625
CV_484	1552.68	57	2	-0.4633
CV_486	3103.25	41	2	0.0307
CV_487	4542.18	56	2	0.4891
CV_488	3069.88	59	2	0.0201
CV_489	1595.41	29	2	-0.4497

CV_491	5418.81	45	2	0.7684
CV_493	521.59	43	2	-0.7918
CV_494	1850.79	52	2	-0.3683
CV_501	923.43	45	2	-0.6638
CV_502	4570.53	34	2	0.4982
CV_504	1992.23	60	2	-0.3233
CV_509	5682	50	2	0.8523
CV_510	1668.114	36	2	-0.4265
CV_511	4314.562	46	2	0.4166
CV_512	1667.372	43	2	-0.4268
CV_513	2339.162	60	2	-0.2127
CV_514	3128.272	46	2	0.0387
CV_515	714.854	31	2	-0.7302
CV_516	2196.124	62	2	-0.2583
CV_518	348.894	65	2	-0.8468
CV_519	1833.006	65	2	-0.3740
CV_520	463.386	60	2	-0.8103
CV_521	1665.258	40	2	-0.4274
CV_522	1853.81	45	2	-0.3674
CV_524	4043.914	28	2	0.3304
CV_530	416.15	42	2	-0.8254
CV_531	82.32	26	2	-0.9317
CV_536	459.98	59	2	-0.8114
CV_538	5682	50	2	0.8523
CV_539	838.18	62	2	-0.6909
CV_540	308.18	30	2	-0.8598
CV_547	5682	54	2	0.8523
CV_551	875	34	2	-0.6792
CV_552	544.31	60	2	-0.7846
CV_553	617.64	29	2	-0.7612
CV_555	521.57	53	2	-0.7918
CV_556	5521.474	49	2	0.8011
CV_557	4588.052	64	2	0.5038
CV_558	3241.014	52	2	0.0746
CV_559	5490.744	57	2	0.7914
CV_560	3261.272	58	2	0.0811
CV_562	7498.708	58	2	1.4311
CV_563	8127.98	63	2	1.6316
CV_564	1184.316	49	2	-0.5807
CV_565	8109.276	59	2	1.6256
CV_566	2999.93	62	2	-0.0022
CV_576	5121.95	33	2	0.6739
CV_578	5673.55	26	2	0.8496
CV_584	6152.01	32	2	1.0020

3 Months post-vaccination

Patient	IgG	Age	Sex	IgG_Z-score
CV_002	1253.52	46	2	0.1670
CV_003	2112.42	56	2	1.0963
CV_004	3490.63	52	1	2.5875
CV_006	2010.18	45	2	0.9857
CV_007	2078.94	27	2	1.0601
CV_008	588.68	40	2	-0.5523
CV_010	855.21	40	2	-0.2640
CV_012	333.54	27	1	-0.8284
CV_013	832.6	48	1	-0.2884
CV_014	820.08	61	2	-0.3020
CV_015	396.58	54	1	-0.7602
CV_017	542.91	39	2	-0.6019
CV_018	261.51	55	2	-0.9063
CV_020	941.93	35	1	-0.1701
CV_021	2059.37	42	1	1.0389
CV_022	1626.92	61	1	0.5710
CV_024	790.74	33	1	-0.3337
CV_025	507.99	63	2	-0.6397
CV_026	923.24	64	1	-0.1904
CV_027	483.86	47	2	-0.6658
CV_028	1113.59	44	2	0.0156
CV_029	2553.76	50	1	1.5738
CV_031	209.9	33	2	-0.9622
CV_032	1059.69	62	2	-0.0427
CV_036	4502.65	65	1	3.6825
CV_038	408.41	51	2	-0.7474
CV_039	1710.3	58	1	0.6612
CV_040	1472.01	55	2	0.4034
CV_041	850.84	62	1	-0.2687
CV_042	1136.78	57	1	0.0407
CV_043	874.95	37	2	-0.2426
CV_044	1225.1	32	2	0.1362
CV_046	1111.59	60	2	0.0134
CV_048	510.56	42	1	-0.6369
CV_049	1582.46	64	1	0.5229
CV_050	374.98	39	2	-0.7836
CV_051	1182.32	34	2	0.0900
CV_052	1233	50	1	0.1448
CV_053	1425.61	53	2	0.3532
CV_054	259.06	56	2	-0.9090
CV_055	1941.64	45	2	0.9115
CV_056	247.38	52	2	-0.9216
CV_058	555.79	65	2	-0.5879
CV_060	461.66	58	2	-0.6898
CV_061	765.86	49	1	-0.3606
CV_062	140.84	34	2	-1.0369
CV_063	227.77	39	2	-0.9428
CV_064	946.63	61	2	-0.1651
CV_065	1938.34	60	2	0.9080
CV_066	3095.53	59	2	2.1600
CV_068	395.5	56	2	-0.7614
CV_069	424.42	50	1	-0.7301
CV_070	1545.5	55	1	0.4829
CV_071	1576.53	53	2	0.5165
CV_072	905.66	39	2	-0.2094
CV_074	875.42	34	1	-0.2421
CV_075	2215.85	53	2	1.2082
CV_076	1605.93	57	1	0.5483
CV_077	681.12	65	2	-0.4523
CV_080	2270.21	50	1	1.2670
CV_082	669.54	50	2	-0.4649
CV_083	1123.53	49	2	0.0263
CV_086	417.84	39	1	-0.7372
CV_088	592.42	49	2	-0.5483
CV_089	799.77	57	2	-0.3240
CV_090	918.47	37	1	-0.1955
CV_091	583.42	44	2	-0.5580
CV_092	1504.69	45	1	0.4388
CV_093	615.7	59	2	-0.5231
CV_095	1075.56	29	1	-0.0256
CV_096	1207.47	59	1	0.1172
CV_098	275.72	45	2	-0.8910
CV_100	240.76	52	2	-0.9288
CV_101	1174.37	53	1	0.0814
CV_103	1474.36	45	2	0.4059
CV_104	1484.74	30	1	0.4172
CV_105	690.42	59	2	-0.4423
CV_106	988.52	41	2	-0.1197
CV_107	1234.97	59	2	0.1469
CV_108	486.93	58	2	-0.6624
CV_110	648.16	49	1	-0.4880
CV_112	850.24	55	2	-0.2693
CV_113	376.1	59	2	-0.7824

		IgG Levels			Age		
	Obs	Median	IQR	Pvalue	Median	IQR	Pvalue
Overall	447	892.09	520.63-1469.24		52	42-59	
Men	153	1031.4	599.37-1626.92	0.012	50	42-57	0.107
Women	294	848.45	501.78-1293.22		53	43-59	

CV_114	928.41	62	2	-0.1848
CV_115	1253.58	59	2	0.1671
CV_116	486.83	48	2	-0.6625
CV_118	1891.17	57	2	0.8569
CV_119	349.22	45	2	-0.8114
CV_123	1515.86	44	2	0.4508
CV_124	2110.93	24	2	1.0947
CV_126	1129.89	36	2	0.0332
CV_127	1095.8	35	2	-0.0037
CV_128	379.65	52	2	-0.7785
CV_129	604.3	64	1	-0.5354
CV_130	1733.93	45	2	0.6868
CV_131	780.11	43	2	-0.3452
CV_133	329.24	54	2	-0.8331
CV_134	178.56	36	2	-0.9961
CV_135	490.07	30	2	-0.6590
CV_136	1461.27	55	2	0.3918
CV_137	417.58	58	2	-0.7375
CV_138	857.21	41	1	-0.2618
CV_139	600.29	54	2	-0.5398
CV_141	252.8	38	1	-0.9158
CV_143	768.69	53	2	-0.3576
CV_144	1356.74	52	1	0.2787
CV_145	246.09	64	2	-0.9230
CV_146	536.19	33	2	-0.6091
CV_150	1322.63	42	1	0.2418
CV_151	523.99	53	1	-0.6223
CV_152	278.01	62	2	-0.8885
CV_155	631.53	61	2	-0.5060
CV_159	463.29	45	1	-0.6880
CV_160	714.15	56	2	-0.4166
CV_162	917.72	44	2	-0.1963
CV_163	1716.51	62	2	0.6679
CV_164	5538.09	42	1	4.8028
CV_165	433.36	57	2	-0.7204
CV_166	803.49	55	2	-0.3199
CV_167	2005.85	26	1	0.9810
CV_168	1095.2	25	2	-0.0043
CV_169	65.21	61	2	-1.1187
CV_170	677.14	63	1	-0.4566
CV_172	922.28	54	2	-0.1914
CV_175	482.36	59	1	-0.6674
CV_176	1421.21	58	2	0.3484
CV_178	1315.3	41	2	0.2338
CV_179	778.59	34	2	-0.3469
CV_180	277.64	53	2	-0.8889
CV_181	1040.86	50	2	-0.0631
CV_183	972.76	50	2	-0.1368
CV_184	260.09	59	2	-0.9079
CV_185	1376.16	43	1	0.2997
CV_186	917.06	52	1	-0.1970
CV_187	253.13	47	2	-0.9154
CV_188	372.81	47	2	-0.7859
CV_190	874.61	47	1	-0.2430
CV_191	1154.3	60	2	0.0596
CV_193	488.59	65	2	-0.6606
CV_194	1440.73	49	1	0.3696
CV_195	1900.88	59	2	0.8674
CV_196	312.57	43	2	-0.8511
CV_199	651.5	60	1	-0.4844
CV_200	594.04	59	1	-0.5465
CV_202	536.73	55	2	-0.6086
CV_203	501.54	49	2	-0.6466
CV_204	1966.25	35	2	0.9382
CV_205	671.6	31	2	-0.4626
CV_206	932.09	59	2	-0.1808
CV_208	1706.44	48	2	0.6570
CV_209	853.02	50	2	-0.2663
CV_210	496.5	60	2	-0.6521
CV_211	796.52	50	2	-0.3275
CV_212	1004.21	44	1	-0.1028
CV_213	1370	54	2	0.2930
CV_214	487.8	44	1	-0.6615
CV_215	378.25	53	1	-0.7800
CV_217	427.16	63	2	-0.7271
CV_218	690.63	54	2	-0.4420
CV_219	1744.34	30	1	0.6981
CV_220	1322.29	23	2	0.2414
CV_221	1261.51	37	2	0.1756
CV_222	436.56	49	2	-0.7169
CV_223	1003.81	59	1	-0.1032
CV_224	204.88	38	2	-0.9676
CV_225	520.66	49	2	-0.6259
CV_226	532.86	55	2	-0.6127
CV_227	1598.65	59	2	0.5404
CV_228	2243.32	44	1	1.2379
CV_229	647.29	59	2	-0.4889
CV_230	735.25	38	2	-0.3938

CV_231	635.12	62	2	-0.5021
CV_232	929.94	29	2	-0.1831
CV_233	308.04	40	2	-0.8560
CV_234	554.45	35	2	-0.5894
CV_236	1045.4	63	2	-0.0582
CV_237	1294.57	49	2	0.2114
CV_238	502.48	40	2	-0.6456
CV_239	545.78	62	2	-0.5988
CV_242	4535.17	64	2	3.7177
CV_244	840.02	61	2	-0.2804
CV_245	2594.92	58	2	1.6184
CV_246	742.45	46	1	-0.3860
CV_247	1091.62	45	2	-0.0082
CV_248	429.45	48	2	-0.7246
CV_249	2692.59	61	2	1.7240
CV_250	620.81	61	2	-0.5176
CV_251	1568.84	60	2	0.5082
CV_254	321.5	39	2	-0.8414
CV_255	520.6	53	2	-0.6260
CV_256	1102.94	52	2	0.0041
CV_257	962.45	62	2	-0.1479
CV_258	1701.5	55	2	0.6517
CV_259	880.46	44	2	-0.2366
CV_260	570.41	57	2	-0.5721
CV_262	978.75	37	2	-0.1303
CV_264	530.24	22	2	-0.6156
CV_265	592.79	63	2	-0.5479
CV_269	1587.35	53	2	0.5282
CV_270	659.8	59	2	-0.4754
CV_273	310.27	51	2	-0.8536
CV_274	604.65	62	2	-0.5351
CV_277	1020.45	43	2	-0.0852
CV_278	988.19	39	2	-0.1201
CV_280	1945.07	49	2	0.9152
CV_281	974.72	62	2	-0.1347
CV_282	328.19	55	2	-0.8342
CV_283	905.19	61	2	-0.2099
CV_284	338.06	23	2	-0.8235
CV_285	901.33	61	2	-0.2141
CV_286	668.96	48	2	-0.4655
CV_287	769.36	52	2	-0.3569
CV_288	390.02	53	2	-0.7673
CV_290	1025.85	57	2	-0.0793
CV_291	1013.89	30	2	-0.0923
CV_293	775.22	58	2	-0.3505
CV_294	386.1	64	2	-0.7715
CV_295	1686.53	54	2	0.6355
CV_296	1206.26	28	2	0.1159
CV_297	868.24	57	2	-0.2499
CV_299	312.76	54	1	-0.8509
CV_300	724.61	62	2	-0.4053
CV_302	11362	62	1	11.1041
CV_305	1992.2	53	1	0.9662
CV_306	916.96	35	2	-0.1972
CV_307	3015.95	53	1	2.0739
CV_308	1117.82	58	2	0.0202
CV_309	1089.44	46	1	-0.0105
CV_310	272.71	47	2	-0.8942
CV_311	440.78	50	1	-0.7124
CV_312	2313.18	59	2	1.3135
CV_313	1556.28	53	1	0.4946
CV_314	944.95	58	2	-0.1669
CV_315	1607.64	27	1	0.5501
CV_317	2565.37	48	2	1.5864
CV_318	884.19	44	1	-0.2326
CV_320	2423.63	62	2	1.4330
CV_321	291.16	42	2	-0.8743
CV_323	1445.83	61	2	0.3751
CV_324	895.03	38	1	-0.2209
CV_325	976.43	55	2	-0.1328
CV_326	359.39	57	1	-0.8004
CV_327	1370.67	43	1	0.2937
CV_328	462.24	27	2	-0.6892
CV_329	630.84	65	2	-0.5067
CV_330	1036.39	52	1	-0.0679
CV_331	934.73	51	1	-0.1779
CV_332	2060.14	53	2	1.0397
CV_333	2556.2	60	2	1.5765
CV_334	1931.61	48	2	0.9007
CV_335	1210.86	38	2	0.1208
CV_336	659.89	36	1	-0.4753
CV_337	746.34	61	1	-0.3818
CV_338	457.11	59	2	-0.6947
CV_339	1185.83	38	2	0.0938
CV_340	1605.96	37	1	0.5483
CV_341	1475.51	51	1	0.4072
CV_346	615.17	52	2	-0.5237
CV_347	739.61	55	2	-0.3890

CV_348	1323.24	24	1	0.2424
CV_349	423.81	23	2	-0.7307
CV_350	501.42	48	2	-0.6468
CV_351	728.87	44	2	-0.4007
CV_352	663.59	27	2	-0.4713
CV_353	212.5	63	1	-0.9594
CV_354	2861.41	55	1	1.9067
CV_355	1085.6	60	1	-0.0147
CV_356	1365.94	58	2	0.2886
CV_362	877.65	37	2	-0.2397
CV_363	1035.89	27	2	-0.0685
CV_369	535.06	54	1	-0.6104
CV_371	561.35	63	1	-0.5819
CV_372	1982.35	41	1	0.9556
CV_373	1095.18	56	1	-0.0043
CV_374	528.54	47	2	-0.6174
CV_375	933.18	54	2	-0.1796
CV_376	615.43	60	1	-0.5234
CV_377	599.92	61	1	-0.5402
CV_378	2528.28	64	1	1.5463
CV_382	640.32	29	1	-0.4965
CV_383	631.16	60	1	-0.5064
CV_384	574.25	53	2	-0.5680
CV_385	992.99	30	2	-0.1149
CV_387	1557.85	44	1	0.4963
CV_388	685.01	48	2	-0.4481
CV_390	758.95	51	2	-0.3681
CV_391	599.37	60	1	-0.5408
CV_392	1700.22	64	2	0.6503
CV_394	1116.7	56	2	0.0190
CV_395	903.3	35	2	-0.2119
CV_396	2597.14	60	1	1.6208
CV_397	1989.85	59	2	0.9637
CV_398	650.74	60	1	-0.4852
CV_399	2148.62	55	2	1.1355
CV_401	1808.92	30	1	0.7679
CV_402	481.75	52	2	-0.6680
CV_403	976.18	39	2	-0.1331
CV_404	1726.11	35	2	0.6783
CV_406	1613.69	60	2	0.5567
CV_407	1888.07	54	1	0.8536
CV_408	1486.4	65	2	0.4190
CV_409	1267.14	63	2	0.1817
CV_410	775.55	37	2	-0.3502
CV_411	340.91	63	1	-0.8204
CV_412	2078.07	60	1	1.0591
CV_413	1423.76	47	2	0.3512
CV_414	538.56	53	2	-0.6066
CV_415	1148.07	43	1	0.0529
CV_416	770.72	61	2	-0.3554
CV_417	846.66	54	2	-0.2732
CV_418	1634.48	46	2	0.5792
CV_419	783.77	39	1	-0.3413
CV_420	568.6	33	2	-0.5741
CV_422	490.88	41	2	-0.6582
CV_423	245.62	34	2	-0.9235
CV_424	1456.37	24	1	0.3865
CV_425	1059.43	64	2	-0.0430
CV_426	2787.94	54	1	1.8272
CV_427	1254.1	63	2	0.1676
CV_428	1562.33	41	2	0.5011
CV_429	565.13	60	2	-0.5778
CV_433	941.94	42	2	-0.1701
CV_434	1104.87	43	1	0.0062
CV_435	785.74	60	2	-0.3391
CV_436	828.5	23	1	-0.2929
CV_437	344.69	48	1	-0.8163
CV_438	1289.19	42	2	0.2056
CV_439	207.22	63	1	-0.9651
CV_441	2194.61	60	2	1.1852
CV_443	363.34	43	2	-0.7962
CV_444	446.65	53	2	-0.7060
CV_445	1227.45	56	2	0.1388
CV_447	1679.53	51	2	0.6279
CV_448	1632.05	33	1	0.5766
CV_450	1988.88	57	2	0.9626
CV_451	1956.21	61	2	0.9273
CV_452	872.7	48	1	-0.2450
CV_453	699.51	40	1	-0.4324
CV_454	394.12	58	2	-0.7629
CV_455	299.61	65	1	-0.8651
CV_456	2033.64	61	2	1.0111
CV_457	778.8	58	2	-0.3466
CV_459	929.43	64	2	-0.1837
CV_460	395.84	42	2	-0.7610
CV_461	1745.22	57	1	0.6990
CV_462	539.39	64	2	-0.6057
CV_463	1023.71	63	2	-0.0817

CV_464	534.96	60	2	-0.6105
CV_467	582.75	54	2	-0.5588
CV_468	1491	58	2	0.4239
CV_469	215.3	56	2	-0.9563
CV_471	348.94	49	1	-0.8117
CV_472	509.23	63	2	-0.6383
CV_473	1466.48	60	2	0.3974
CV_474	1504.77	23	2	0.4388
CV_475	491.12	53	2	-0.6579
CV_476	946.5	56	2	-0.1652
CV_477	1280.67	33	1	0.1964
CV_479	572.19	56	2	-0.5702
CV_480	1150.13	58	2	0.0551
CV_481	1264.34	35	1	0.1787
CV_482	880.29	41	2	-0.2368
CV_483	1736.36	61	2	0.6894
CV_484	987.74	57	2	-0.1206
CV_486	729.85	41	2	-0.3996
CV_487	1623.67	56	2	0.5675
CV_488	832.49	60	2	-0.2885
CV_490	665.78	63	1	-0.4689
CV_491	2997.92	45	2	2.0544
CV_492	423.22	42	1	-0.7314
CV_493	150.89	43	2	-1.0260
CV_494	748.64	52	2	-0.3793
CV_496	1292.56	57	1	0.2092
CV_497	1632.67	34	1	0.5772
CV_498	726.97	29	1	-0.4027
CV_499	298.27	63	1	-0.8666
CV_500	1472.03	49	1	0.4034
CV_501	312.03	45	2	-0.8517
CV_502	892.09	34	2	-0.2241
CV_503	677.37	51	1	-0.4564
CV_505	126.54	65	1	-1.0524
CV_506	391.17	31	1	-0.7660
CV_507	816.32	56	1	-0.3060
CV_508	3619.96	26	1	2.7274
CV_509	4014.38	50	2	3.1542
CV_510	524.65	37	2	-0.6216
CV_511	928.23	46	2	-0.1850
CV_512	532.86	43	2	-0.6127
CV_513	926.82	60	2	-0.1865
CV_514	754.42	46	2	-0.3730
CV_515	228.62	32	2	-0.9419
CV_516	1196.51	63	2	0.1053
CV_517	1793.05	66	1	0.7508
CV_518	144.93	65	2	-1.0325
CV_519	417.27	66	2	-0.7378
CV_520	192.58	60	2	-0.9809
CV_522	494.09	45	2	-0.6547
CV_523	1031.4	63	1	-0.0733
CV_524	1459.86	28	2	0.3903
CV_525	1281.55	53	1	0.1973
CV_526	1228.02	30	1	0.1394
CV_527	464.1	63	1	-0.6871
CV_529	442.32	57	1	-0.7107
CV_530	79.85	42	2	-1.1029
CV_531	53.41	26	2	-1.1315
CV_532	20.66	63	1	-1.1669
CV_533	219.87	52	1	-0.9514
CV_535	3488.27	24	1	2.5849
CV_536	119.22	60	2	-1.0603
CV_537	436.2	33	1	-0.7173
CV_538	1907.57	50	2	0.8747
CV_539	203.37	62	2	-0.9692
CV_541	139.33	44	1	-1.0385
CV_542	3324.94	43	1	2.4082
CV_543	226.29	28	1	-0.9444
CV_544	1750.99	45	1	0.7052
CV_545	4160.86	39	1	3.3127
CV_548	1677.47	34	1	0.6257
CV_549	142.78	46	1	-1.0348
CV_551	377.69	34	2	-0.7806
CV_553	190.29	29	2	-0.9834
CV_554	204.55	50	1	-0.9680
CV_556	2112.39	49	2	1.0963
CV_557	1225.2	64	2	0.1364
CV_558	828.13	52	2	-0.2933
CV_559	1629.24	57	2	0.5735
CV_560	1609.53	59	2	0.5522
CV_562	2364.94	58	2	1.3695
CV_563	2359.39	63	2	1.3635
CV_564	361.59	50	2	-0.7981
CV_565	3506.08	59	2	2.6042
CV_566	1580.5	62	2	0.5208
CV_567	1674.89	50	1	0.6229
CV_568	3548.45	43	1	2.6501
CV_569	292.45	60	1	-0.8729

CV_572	446.01	27	1	-0.7067
CV_573	1860.37	64	1	0.8236
CV_574	2964.76	51	1	2.0185
CV_575	1227.35	53	1	0.1387
CV_576	1871.25	33	2	0.8354
CV_577	3057.8	51	1	2.1192
CV_579	726.61	49	1	-0.4031
CV_580	1069.74	53	1	-0.0318
CV_581	1192.74	43	1	0.1012
CV_582	1662.92	52	1	0.6100
CV_583	2611.59	56	1	1.6364
CV_584	2675.54	32	2	1.7056

1 vs. 3 Months post-vaccination

Patient	IgG 1M	IgG 3M	Age	Sex	Total Difference	% Drop in antibodies	Diff IgG_Z-score
CV_002	1736.96	1253.52	46	2	483.440	27.833	-0.5472
CV_003	4872.94	2112.42	56	2	2760.520	56.650	0.2661
CV_004	8975.99	3490.63	52	1	5485.360	61.111	1.2395
CV_006	4910.06	2010.18	45	2	2899.880	59.060	0.3159
CV_007	871.84	2078.94	27	2	-1207.100	-138.454	-0.2888
CV_008	1855.76	588.68	40	2	1267.080	68.278	-0.2673
CV_010	2007.07	855.21	40	2	1151.860	57.390	-0.3085
CV_012	1651.55	333.54	27	1	1318.010	79.804	-0.2491
CV_013	2216.32	832.6	48	1	1383.720	62.433	-0.2257
CV_014	1590.83	820.08	61	2	770.750	48.450	-0.4446
CV_015	1583.87	396.58	54	1	1187.290	74.961	-0.2958
CV_017	1642.14	542.91	39	2	1099.230	66.939	-0.3273
CV_018	951.03	261.51	55	2	689.520	72.502	-0.4736
CV_020	3445.83	941.93	35	1	2503.900	72.665	0.1745
CV_021	5204.1	2059.37	42	1	3144.730	60.428	0.4034
CV_022	4693.48	1626.92	61	1	3066.560	65.337	0.3755
CV_024	2340.03	790.74	33	1	1549.290	66.208	-0.1665
CV_025	2236.67	507.99	63	2	1728.680	77.288	-0.1024
CV_026	1934.89	923.24	64	1	1011.650	52.285	-0.3586
CV_027	953.69	483.86	47	2	469.830	49.264	-0.5521
CV_028	3922.61	1113.59	44	2	2809.020	71.611	0.2835
CV_029	5682	2553.76	50	1	3128.240	55.055	0.3975
CV_031	794.52	209.9	33	2	584.620	73.582	-0.5111
CV_032	2103.374	1059.69	62	2	1043.684	49.620	-0.3471
CV_036	1387.568	4502.65	65	1	-3115.082	-224.499	0.3928
CV_038	1339.63	408.41	51	2	931.220	69.513	-0.3873
CV_039	4558.34	1710.3	58	1	2848.040	62.480	0.2974
CV_040	4604.62	1472.01	55	2	3132.610	68.032	0.3990
CV_041	2747.67	850.84	62	1	1896.830	69.034	-0.0424
CV_042	3312.01	1136.78	57	1	2175.230	65.677	0.0571
CV_043	2049.4	874.95	37	2	1174.450	57.307	-0.3004
CV_044	2087.58	1225.1	32	2	862.480	41.315	-0.4118
CV_046	3162.33	1111.59	60	2	2050.740	64.849	0.0126
CV_048	1812.83	510.56	42	1	1302.270	71.836	-0.2548
CV_049	5304.28	1582.46	64	1	3721.820	70.166	0.6095
CV_050	1356.14	374.98	39	2	981.160	72.349	-0.3695
CV_051	2283.46	1182.32	34	2	1101.140	48.222	-0.3266
CV_052	2283.67	1233	50	1	1050.670	46.008	-0.3446
CV_053	2921.25	1425.61	53	2	1495.640	51.199	-0.1857
CV_054	577.64	259.06	56	2	318.580	55.152	-0.6061
CV_055	4192.59	1941.64	45	2	2250.950	53.689	0.0841
CV_056	729.68	247.38	52	2	482.300	66.097	-0.5477
CV_058	1472.7	555.79	65	2	916.910	62.260	-0.3924
CV_060	1365.83	461.66	58	2	904.170	66.199	-0.3970
CV_061	1992.6	765.86	49	1	1226.740	61.565	-0.2817
CV_062	1208.68	140.84	34	2	1067.840	88.348	-0.3385
CV_063	1075.76	227.77	39	2	847.990	78.827	-0.4170
CV_064	1625.18	946.63	61	2	678.550	41.752	-0.4775
CV_065	4277.92	1938.34	60	2	2339.580	54.690	0.1158
CV_066	5682	3095.53	59	2	2586.470	45.520	0.2040
CV_068	1445.57	395.5	56	2	1050.070	72.641	-0.3448
CV_069	1324.79	424.42	50	1	900.370	67.963	-0.3983
CV_070	2853.99	1545.5	55	1	1308.490	45.848	-0.2525
CV_071	4953.54	1576.53	53	2	3377.010	68.174	0.4863
CV_072	3118.11	905.66	39	2	2212.450	70.955	0.0704
CV_074	2356.28	875.42	34	1	1480.860	62.847	-0.1910
CV_075	4436.69	2215.85	53	2	2220.840	50.056	0.0734
CV_076	3469.97	1605.93	57	1	1864.040	53.719	-0.0541
CV_077	2413.72	681.12	65	2	1732.600	71.781	-0.1010
CV_080	4000.34	2270.21	50	1	1730.130	43.250	-0.1019
CV_082	2023.27	669.54	50	2	1353.730	66.908	-0.2364
CV_083	2888.96	1123.53	49	2	1765.430	61.110	-0.0893
CV_086	778.9	417.84	39	1	361.060	46.355	-0.5910
CV_088	2065.63	592.42	49	2	1473.210	71.320	-0.1937
CV_089	1595.04	799.77	57	2	795.270	49.859	-0.4359
CV_090	2537.3	918.47	37	1	1618.830	63.801	-0.1417
CV_091	1196.04	583.42	44	2	612.620	51.221	-0.5011
CV_092	3597.17	1504.69	45	1	2092.480	58.170	0.0275
CV_093	1675.44	615.7	59	2	1059.740	63.251	-0.3414
CV_095	2076.93	1075.56	29	1	1001.370	48.214	-0.3622
CV_096	2270.32	1207.47	59	1	1062.850	46.815	-0.3403
CV_098	937.63	275.72	45	2	661.910	70.594	-0.4835
CV_100	892.56	240.76	52	2	651.800	73.026	-0.4871
CV_101	3304.6	1174.37	53	1	2130.230	64.463	0.0410
CV_103	4081.99	1474.36	45	2	2607.630	63.881	0.2115
CV_104	3993.52	1484.74	30	1	2508.780	62.821	0.1762
CV_105	2481.58	690.42	59	2	1791.160	72.178	-0.0801
CV_106	3770.97	988.52	41	2	2782.450	73.786	0.2740
CV_107	2857.92	1234.97	59	2	1622.950	56.788	-0.1402
CV_108	1030.21	486.93	58	2	543.280	52.735	-0.5259
CV_110	2094.37	648.16	49	1	1446.210	69.052	-0.2033
CV_112	2460.68	850.24	55	2	1610.440	65.447	-0.1447
CV_113	1415.56	376.1	59	2	1039.460	73.431	-0.3486

		IgG Levels			Age		
	Obs	Median	IQR	Pvalue	Median	IQR	Pvalue
Overall	447	1526.62	998.30-2401.49		52	42-59	
Men	153	1624.03	1066.48-2694.13	0.043	50	42-57	0.107
Women	294	1472.31	935.73-2251.05		53	43-59	

CV_114	2853.48	928.41	62	2	1925.070	67.464	-0.0323
CV_115	3658.84	1253.58	59	2	2405.260	65.738	0.1392
CV_116	1684.99	486.83	48	2	1198.160	71.108	-0.2919
CV_118	4510.26	1891.17	57	2	2619.090	58.070	0.2156
CV_119	1259.58	349.22	45	2	910.360	72.275	-0.3947
CV_123	5443.71	1515.86	44	2	3927.850	72.154	0.6831
CV_124	3467.36	2110.93	24	2	1356.430	39.120	-0.2354
CV_126	2429.19	1129.89	36	2	1299.300	53.487	-0.2558
CV_127	2270.2	1095.8	35	2	1174.400	51.731	-0.3004
CV_128	1506.31	379.65	52	2	1126.660	74.796	-0.3175
CV_129	2067.89	604.3	64	1	1463.590	70.777	-0.1971
CV_130	3838.77	1733.93	45	2	2104.840	54.831	0.0319
CV_131	2434.94	780.11	43	2	1654.830	67.962	-0.1288
CV_133	1044.07	329.24	54	2	714.830	68.466	-0.4646
CV_134	506.34	178.56	36	2	327.780	64.735	-0.6028
CV_135	1456.15	490.07	30	2	966.080	66.345	-0.3748
CV_136	4315.96	1461.27	55	2	2854.690	66.143	0.2998
CV_137	1074.91	417.58	58	2	657.330	61.152	-0.4851
CV_138	2103.43	857.21	41	1	1246.220	59.247	-0.2748
CV_139	1719.71	600.29	54	2	1119.420	65.094	-0.3201
CV_141	1539.34	252.8	38	1	1286.540	83.577	-0.2604
CV_143	1477.6	768.69	53	2	708.910	47.977	-0.4667
CV_144	3041.81	1356.74	52	1	1685.070	55.397	-0.1180
CV_145	1418.62	246.09	64	2	1172.530	82.653	-0.3011
CV_146	1520.91	536.19	33	2	984.720	64.745	-0.3682
CV_150	1813.11	1322.63	42	1	490.480	27.052	-0.5447
CV_151	1106.82	523.99	53	1	582.830	52.658	-0.5117
CV_152	1099.05	278.01	62	2	821.040	74.705	-0.4267
CV_155	2213.2	631.53	61	2	1581.670	71.465	-0.1550
CV_159	1446.58	463.29	45	1	983.290	67.973	-0.3687
CV_160	1859.14	714.15	56	2	1144.990	61.587	-0.3109
CV_162	2439.23	917.72	44	2	1521.510	62.377	-0.1764
CV_163	3052.39	1716.51	62	2	1335.880	43.765	-0.2428
CV_164	37360	5538.09	42	1	31821.910	85.176	10.6469
CV_165	1074.24	433.36	57	2	640.880	59.659	-0.4910
CV_166	2508.49	803.49	55	2	1705.000	67.969	-0.1109
CV_167	4138.58	2005.85	26	1	2132.730	51.533	0.0419
CV_168	2552.17	1095.2	25	2	1456.970	57.087	-0.1995
CV_169	113.54	65.21	61	2	48.330	42.566	-0.7027
CV_170	2187.42	677.14	63	1	1510.280	69.044	-0.1805
CV_172	2488.64	922.28	54	2	1566.360	62.940	-0.1604
CV_175	1086.85	482.36	59	1	604.490	55.619	-0.5040
CV_176	3660.67	1421.21	58	2	2239.460	61.176	0.0800
CV_178	2557.92	1315.3	41	2	1242.620	48.579	-0.2761
CV_179	1459.08	778.59	34	2	680.490	46.638	-0.4769
CV_180	820.82	277.64	53	2	543.180	66.175	-0.5259
CV_181	3880.75	1040.86	50	2	2839.890	73.179	0.2945
CV_183	2432.56	972.76	50	2	1459.800	60.011	-0.1985
CV_184	950.16	260.09	59	2	690.070	72.627	-0.4734
CV_185	2476.48	1376.16	43	1	1100.320	44.431	-0.3269
CV_186	2424.24	917.06	52	1	1507.180	62.171	-0.1816
CV_187	1590.09	253.13	47	2	1336.960	84.081	-0.2424
CV_188	1944.14	372.81	47	2	1571.330	80.824	-0.1586
CV_190	3185.09	874.61	47	1	2310.480	72.540	0.1054
CV_191	5041.23	1154.3	60	2	3886.930	77.103	0.6685
CV_193	1544.07	488.59	65	2	1055.480	68.357	-0.3429
CV_194	3676.49	1440.73	49	1	2235.760	60.812	0.0787
CV_195	3732.92	1900.88	59	2	1832.040	49.078	-0.0655
CV_196	1092.12	312.57	43	2	779.550	71.380	-0.4415
CV_199	1715.77	651.5	60	1	1064.270	62.029	-0.3398
CV_200	1942.08	594.04	59	1	1348.040	69.412	-0.2384
CV_202	3369.52	536.73	55	2	2832.790	84.071	0.2919
CV_203	1617.47	501.54	49	2	1115.930	68.992	-0.3213
CV_204	3753.57	1966.25	35	2	1787.320	47.617	-0.0815
CV_205	2170.53	671.6	31	2	1498.930	69.058	-0.1845
CV_206	2907.42	932.09	59	2	1975.330	67.941	-0.0143
CV_208	5593.76	1706.44	48	2	3887.320	69.494	0.6686
CV_209	2435.97	853.02	50	2	1582.950	64.982	-0.1545
CV_210	965.43	496.5	60	2	468.930	48.572	-0.5524
CV_211	3648.63	796.52	50	2	2852.110	78.169	0.2989
CV_212	2682.39	1004.21	44	1	1678.180	62.563	-0.1205
CV_213	3647.03	1370	54	2	2277.030	62.435	0.0934
CV_214	2265.88	487.8	44	1	1778.080	78.472	-0.0848
CV_215	1195.85	378.25	53	1	817.600	68.370	-0.4279
CV_217	1035.49	427.16	63	2	608.330	58.748	-0.5026
CV_218	1422.75	690.63	54	2	732.120	51.458	-0.4584
CV_219	4151.21	1744.34	30	1	2406.870	57.980	0.1398
CV_220	3725.84	1322.29	23	2	2403.550	64.510	0.1386
CV_221	3207.51	1261.51	37	2	1946.000	60.670	-0.0248
CV_222	1472.11	436.56	49	2	1035.550	70.345	-0.3500
CV_223	4458.87	1003.81	59	1	3455.060	77.487	0.5142
CV_224	506.16	204.88	38	2	301.280	59.523	-0.6123
CV_225	1397.73	520.66	49	2	877.070	62.750	-0.4066
CV_226	1253.5	532.86	55	2	720.640	57.490	-0.4625
CV_227	3200	1598.65	59	2	1601.350	50.042	-0.1479
CV_228	4176.93	2243.32	44	1	1933.610	46.293	-0.0292
CV_229	1809.59	647.29	59	2	1162.300	64.230	-0.3048
CV_230	1955.92	735.25	38	2	1220.670	62.409	-0.2839

CV_231	1906.59	635.12	62	2	1271.470	66.688	-0.2658
CV_232	1297.23	929.94	29	2	367.290	28.313	-0.5887
CV_233	746.37	308.04	40	2	438.330	58.728	-0.5634
CV_234	1700.86	554.45	35	2	1146.410	67.402	-0.3104
CV_236	2481.41	1045.4	63	2	1436.010	57.871	-0.2070
CV_237	4909	1294.57	49	2	3614.430	73.629	0.5712
CV_238	1467.04	502.48	40	2	964.560	65.749	-0.3754
CV_239	1542.76	545.78	62	2	996.980	64.623	-0.3638
CV_242	10705.52	4535.17	64	2	6170.350	57.637	1.4841
CV_244	2208.8	840.02	61	2	1368.780	61.969	-0.2310
CV_245	5270.02	2594.92	58	2	2675.100	50.761	0.2356
CV_246	3101.54	742.45	46	1	2359.090	76.062	0.1227
CV_247	2268.05	1091.62	45	2	1176.430	51.870	-0.2997
CV_248	974.82	429.45	48	2	545.370	55.946	-0.5251
CV_249	4943.67	2692.59	61	2	2251.080	45.535	0.0842
CV_250	2510.23	620.81	61	2	1889.420	75.269	-0.0450
CV_251	3474.6	1568.84	60	2	1905.760	54.848	-0.0392
CV_254	1011.05	321.5	39	2	689.550	68.201	-0.4736
CV_255	1470.04	520.6	53	2	949.440	64.586	-0.3808
CV_256	2876.41	1102.94	52	2	1773.470	61.656	-0.0864
CV_257	1810.98	962.45	62	2	848.530	46.855	-0.4168
CV_258	3923.09	1701.5	55	2	2221.590	56.629	0.0736
CV_259	2605.46	880.46	44	2	1725.000	66.207	-0.1038
CV_260	1634.36	570.41	57	2	1063.950	65.099	-0.3399
CV_262	2349.45	978.75	37	2	1370.700	58.341	-0.2303
CV_264	1678.61	530.24	22	2	1148.370	68.412	-0.3097
CV_265	1497.18	592.79	63	2	904.390	60.406	-0.3969
CV_269	3092.26	1587.35	53	2	1504.910	48.667	-0.1824
CV_270	2901.8	659.8	59	2	2242.000	77.262	0.0809
CV_273	1100.39	310.27	51	2	790.120	71.804	-0.4377
CV_274	1248.79	604.65	62	2	644.140	51.581	-0.4898
CV_277	4573.93	1020.45	43	2	3553.480	77.690	0.5494
CV_278	2447.13	988.19	39	2	1458.940	59.618	-0.1988
CV_280	5633.78	1945.07	49	2	3688.710	65.475	0.5977
CV_281	2782.36	974.72	62	2	1807.640	64.968	-0.0742
CV_282	937.46	328.19	55	2	609.270	64.992	-0.5023
CV_283	2313.11	905.19	61	2	1407.920	60.867	-0.2170
CV_284	884.63	338.06	23	2	546.570	61.785	-0.5247
CV_285	1704.81	901.33	61	2	803.480	47.130	-0.4329
CV_286	2409.38	668.96	48	2	1740.420	72.235	-0.0982
CV_287	2295.98	769.36	52	2	1526.620	66.491	-0.1746
CV_288	1563.41	390.02	53	2	1173.390	75.053	-0.3008
CV_290	2951.65	1025.85	57	2	1925.800	65.245	-0.0320
CV_291	4091.19	1013.89	30	2	3077.300	75.218	0.3793
CV_293	1518.21	775.22	58	2	742.990	48.939	-0.4545
CV_294	1142.62	386.1	64	2	756.520	66.209	-0.4497
CV_295	3905.09	1686.53	54	2	2218.560	56.812	0.0725
CV_296	2208.37	1206.26	28	2	1002.110	45.378	-0.3620
CV_297	2675.24	868.24	57	2	1807.000	67.545	-0.0745
CV_299	2133.66	312.76	54	1	1820.900	85.342	-0.0695
CV_300	2565.23	724.61	62	2	1840.620	71.753	-0.0625
CV_302	38000	11362	62	1	26638.000	70.100	8.7952
CV_305	5294.81	1992.2	53	1	3302.610	62.374	0.4598
CV_306	3897.91	916.96	35	2	2980.950	76.476	0.3449
CV_307	7305.73	3015.95	53	1	4289.780	58.718	0.8124
CV_308	4721.67	1117.82	58	2	3603.850	76.326	0.5674
CV_309	3913.52	1089.44	46	1	2824.080	72.162	0.2888
CV_310	1409.97	272.71	47	2	1137.260	80.658	-0.3137
CV_311	1162.24	440.78	50	1	721.460	62.075	-0.4622
CV_312	5682	2313.18	59	2	3368.820	59.289	0.4834
CV_313	4855.07	1556.28	53	1	3298.790	67.945	0.4584
CV_314	2978.42	944.95	58	2	2033.470	68.273	0.0064
CV_315	4817.41	1607.64	27	1	3209.770	66.629	0.4266
CV_317	4998.67	2565.37	48	2	2433.300	48.679	0.1493
CV_318	3435.14	884.19	44	1	2550.950	74.260	0.1913
CV_320	5682	2423.63	62	2	3258.370	57.345	0.4440
CV_321	1366.93	291.16	42	2	1075.770	78.700	-0.3357
CV_323	5119.57	1445.83	61	2	3673.740	71.759	0.5923
CV_324	3684.7	895.03	38	1	2789.670	75.710	0.2765
CV_325	2842.58	976.43	55	2	1866.150	65.650	-0.0533
CV_326	1136.14	359.39	57	1	776.750	68.367	-0.4425
CV_327	3684.05	1370.67	43	1	2313.380	62.794	0.1064
CV_328	1731.51	462.24	27	2	1269.270	73.304	-0.2665
CV_329	2195.33	630.84	65	2	1564.490	71.264	-0.1611
CV_330	2960.69	1036.39	52	1	1924.300	64.995	-0.0326
CV_331	2530.67	934.73	51	1	1595.940	63.064	-0.1499
CV_332	7240.3	2060.14	53	2	5180.160	71.546	1.1304
CV_333	8166.93	2556.2	60	2	5610.730	68.701	1.2842
CV_334	4007.82	1931.61	48	2	2076.210	51.804	0.0217
CV_335	2116.99	1210.86	38	2	906.130	42.803	-0.3963
CV_336	1340.25	659.89	36	1	680.360	50.764	-0.4769
CV_337	2811.05	746.34	61	1	2064.710	73.450	0.0176
CV_338	3366.22	457.11	59	2	2909.110	86.421	0.3192
CV_339	4665.88	1185.83	38	2	3480.050	74.585	0.5232
CV_340	4300.09	1605.96	37	1	2694.130	62.653	0.2424
CV_341	6601.01	1475.51	51	1	5125.500	77.647	1.1109
CV_346	2155.23	615.17	52	2	1540.060	71.457	-0.1698
CV_347	2417.68	739.61	55	2	1678.070	69.408	-0.1205

CV_348	3378.11	1323.24	24	1	2054.870	60.829	0.0141
CV_349	1487.99	423.81	23	2	1064.180	71.518	-0.3398
CV_350	1685.64	501.42	48	2	1184.220	70.253	-0.2969
CV_351	2677.95	728.87	44	2	1949.080	72.783	-0.0237
CV_352	1838.6	663.59	27	2	1175.010	63.908	-0.3002
CV_353	1686.02	212.5	63	1	1473.520	87.396	-0.1936
CV_354	5682	2861.41	55	1	2820.590	49.641	0.2876
CV_355	4128.12	1085.6	60	1	3042.520	73.702	0.3669
CV_356	3703.8	1365.94	58	2	2337.860	63.121	0.1152
CV_362	2906.36	877.65	37	2	2028.710	69.802	0.0047
CV_363	3435.32	1035.89	27	2	2399.430	69.846	0.1372
CV_369	1789.47	535.06	54	1	1254.410	70.100	-0.2719
CV_371	2252.32	561.35	63	1	1690.970	75.077	-0.1159
CV_372	4795.16	1982.35	41	1	2812.810	58.659	0.2848
CV_373	2413.67	1095.18	56	1	1318.490	54.626	-0.2490
CV_374	1061.62	528.54	47	2	533.080	50.214	-0.5295
CV_375	1831.62	933.18	54	2	898.440	49.052	-0.3990
CV_376	2097.92	615.43	60	1	1482.490	70.665	-0.1904
CV_377	1861.04	599.92	61	1	1261.120	67.764	-0.2695
CV_378	41360	2528.28	64	1	38831.720	93.887	13.1508
CV_382	1847.06	640.32	29	1	1206.740	65.333	-0.2889
CV_383	1697.64	631.16	60	1	1066.480	62.821	-0.3390
CV_384	2260.3	574.25	53	2	1686.050	74.594	-0.1177
CV_385	2571.88	992.99	30	2	1578.890	61.391	-0.1559
CV_387	3838.29	1557.85	44	1	2280.440	59.413	0.0946
CV_388	1356.71	685.01	48	2	671.700	49.509	-0.4800
CV_390	2197.82	758.95	51	2	1438.870	65.468	-0.2060
CV_391	3468.39	599.37	60	1	2869.020	82.719	0.3049
CV_392	6862.83	1700.22	64	2	5162.610	75.226	1.1242
CV_394	4055.78	1116.7	56	2	2939.080	72.466	0.3299
CV_395	2576.11	903.3	35	2	1672.810	64.936	-0.1224
CV_396	4150.38	2597.14	60	1	1553.240	37.424	-0.1651
CV_397	4146.16	1989.85	59	2	2156.310	52.007	0.0503
CV_398	2790.27	650.74	60	1	2139.530	76.678	0.0443
CV_399	4621.73	2148.62	55	2	2473.110	53.510	0.1635
CV_401	4900.15	1808.92	30	1	3091.230	63.084	0.3843
CV_402	1235.76	481.75	52	2	754.010	61.016	-0.4506
CV_403	3803.56	976.18	39	2	2827.380	74.335	0.2900
CV_404	1970.7	1726.11	35	2	244.590	12.411	-0.6326
CV_406	3906.83	1613.69	60	2	2293.140	58.696	0.0992
CV_407	4444.07	1888.07	54	1	2556.000	57.515	0.1931
CV_408	4688.39	1486.4	65	2	3201.990	68.296	0.4238
CV_409	5538	1267.14	63	2	4270.860	77.119	0.8056
CV_410	2482.69	775.55	37	2	1707.140	68.762	-0.1101
CV_411	1227.96	340.91	63	1	887.050	72.238	-0.4031
CV_412	4736.48	2078.07	60	1	2658.410	56.126	0.2297
CV_413	3345.89	1423.76	47	2	1922.130	57.447	-0.0333
CV_414	2364.43	538.56	53	2	1825.870	77.222	-0.0677
CV_415	2150.56	1148.07	43	1	1002.490	46.615	-0.3618
CV_416	2577.57	770.72	61	2	1806.850	70.099	-0.0745
CV_417	2560.15	846.66	54	2	1713.490	66.929	-0.1079
CV_418	3746.19	1634.48	46	2	2111.710	56.370	0.0344
CV_419	2249.12	783.77	39	1	1465.350	65.152	-0.1965
CV_420	1641.29	568.6	33	2	1072.690	65.357	-0.3368
CV_422	1602.82	490.88	41	2	1111.940	69.374	-0.3227
CV_423	1194.87	245.62	34	2	949.250	79.444	-0.3809
CV_424	3832.92	1456.37	24	1	2376.550	62.004	0.1290
CV_425	3678.37	1059.43	64	2	2618.940	71.198	0.2156
CV_426	5401.69	2787.94	54	1	2613.750	48.388	0.2137
CV_427	3695.69	1254.1	63	2	2441.590	66.066	0.1522
CV_428	4414.03	1562.33	41	2	2851.700	64.605	0.2987
CV_429	1028.42	565.13	60	2	463.290	45.049	-0.5544
CV_433	3502.49	941.94	42	2	2560.550	73.107	0.1947
CV_434	2529.52	1104.87	43	1	1424.650	56.321	-0.2110
CV_435	2622.16	785.74	60	2	1836.420	70.035	-0.0640
CV_436	2275.72	828.5	23	1	1447.220	63.594	-0.2030
CV_437	935.91	344.69	48	1	591.220	63.171	-0.5087
CV_438	2752.77	1289.19	42	2	1463.580	53.168	-0.1971
CV_439	762.75	207.22	63	1	555.530	72.833	-0.5215
CV_441	4673.58	2194.61	60	2	2478.970	53.042	0.1656
CV_443	1132.48	363.34	43	2	769.140	67.916	-0.4452
CV_444	1636.38	446.65	53	2	1189.730	72.705	-0.2950
CV_445	2782.31	1227.45	56	2	1554.860	55.884	-0.1645
CV_447	4121.32	1679.53	51	2	2441.790	59.248	0.1523
CV_448	4183.14	1632.05	33	1	2551.090	60.985	0.1913
CV_450	6676.09	1988.88	57	2	4687.210	70.209	0.9544
CV_451	4155.98	1956.21	61	2	2199.770	52.930	0.0658
CV_452	2713.19	872.7	48	1	1840.490	67.835	-0.0625
CV_453	2749.94	699.51	40	1	2050.430	74.563	0.0125
CV_454	1430.35	394.12	58	2	1036.230	72.446	-0.3498
CV_455	1549.04	299.61	65	1	1249.430	80.658	-0.2736
CV_456	9111.9	2033.64	61	2	7078.260	77.681	1.8084
CV_457	2385.3	778.8	58	2	1606.500	67.350	-0.1461
CV_459	2951.43	929.43	64	2	2022.000	68.509	0.0023
CV_460	1544.25	395.84	42	2	1148.410	74.367	-0.3097
CV_461	7283.95	1745.22	57	1	5538.730	76.040	1.2585
CV_462	1314.82	539.39	64	2	775.430	58.976	-0.4429
CV_463	5375	1023.71	63	2	4351.290	80.954	0.8344

CV_464	1037.69	534.96	60	2	502.730	48.447	-0.5404
CV_467	2785.93	582.75	54	2	2203.180	79.082	0.0671
CV_468	3355.47	1491	58	2	1864.470	55.565	-0.0539
CV_469	952.35	215.3	56	2	737.050	77.393	-0.4567
CV_471	3172.07	348.94	49	1	2823.130	89.000	0.2885
CV_472	1527.83	509.23	63	2	1018.600	66.670	-0.3561
CV_473	3387.57	1466.48	60	2	1921.090	56.710	-0.0337
CV_474	3867.21	1504.77	23	2	2362.440	61.089	0.1239
CV_475	1524.41	491.12	53	2	1033.290	67.783	-0.3508
CV_476	2417.9	946.5	56	2	1471.400	60.854	-0.1943
CV_477	2503.33	1280.67	33	1	1222.660	48.841	-0.2832
CV_479	2711.94	572.19	56	2	2139.750	78.901	0.0444
CV_480	2546.56	1150.13	58	2	1396.430	54.836	-0.2211
CV_481	2232.28	1264.34	35	1	967.940	43.361	-0.3742
CV_482	2800.74	880.29	41	2	1920.450	68.569	-0.0339
CV_483	4144.72	1736.36	61	2	2408.360	58.107	0.1403
CV_484	1552.68	987.74	57	2	564.940	36.385	-0.5181
CV_486	3103.25	729.85	41	2	2373.400	76.481	0.1279
CV_487	4542.18	1623.67	56	2	2918.510	64.254	0.3226
CV_488	3069.88	832.49	60	2	2237.390	72.882	0.0793
CV_490	2289.81	665.78	63	1	1624.030	70.924	-0.1398
CV_491	5418.81	2997.92	45	2	2420.890	44.676	0.1448
CV_492	1600.85	423.22	42	1	1177.630	73.563	-0.2993
CV_493	521.59	150.89	43	2	370.700	71.071	-0.5875
CV_494	1850.79	748.64	52	2	1102.150	59.550	-0.3262
CV_496	3008.6	1292.56	57	1	1716.040	57.038	-0.1070
CV_497	2004.23	1632.67	34	1	371.560	18.539	-0.5872
CV_498	1528.74	726.97	29	1	801.770	52.446	-0.4335
CV_499	1338.7	298.27	63	1	1040.430	77.719	-0.3483
CV_500	2837.67	1472.03	49	1	1365.640	48.125	-0.2321
CV_501	923.43	312.03	45	2	611.400	66.210	-0.5015
CV_502	4570.53	892.09	34	2	3678.440	80.482	0.5940
CV_503	1431.35	677.37	51	1	753.980	52.676	-0.4506
CV_505	382.43	126.54	65	1	255.890	66.912	-0.6285
CV_506	1107.73	391.17	31	1	716.560	64.687	-0.4640
CV_507	2381.94	816.32	56	1	1565.620	65.729	-0.1607
CV_508	5682	3619.96	26	1	2062.040	36.291	0.0166
CV_509	5682	4014.38	50	2	1667.620	29.349	-0.1243
CV_510	1668.114	524.65	37	2	1143.464	68.548	-0.3115
CV_511	4314.562	928.23	46	2	3386.332	78.486	0.4897
CV_512	1667.372	532.86	43	2	1134.512	68.042	-0.3147
CV_513	2339.162	926.82	60	2	1412.342	60.378	-0.2154
CV_514	3128.272	754.42	46	2	2373.852	75.884	0.1280
CV_515	714.854	228.62	32	2	486.234	68.019	-0.5462
CV_516	2196.124	1196.51	63	2	999.614	45.517	-0.3629
CV_517	6789.734	1793.05	66	1	4996.684	73.592	1.0649
CV_518	348.894	144.93	65	2	203.964	58.460	-0.6471
CV_519	1833.006	417.27	66	2	1415.736	77.236	-0.2142
CV_520	463.386	192.58	60	2	270.806	58.441	-0.6232
CV_522	1853.81	494.09	45	2	1359.720	73.347	-0.2342
CV_523	1545.404	1031.4	63	1	514.004	33.260	-0.5363
CV_524	4043.914	1459.86	28	2	2584.054	63.900	0.2031
CV_525	4014.318	1281.55	53	1	2732.768	68.076	0.2562
CV_526	4226.796	1228.02	30	1	2998.776	70.947	0.3512
CV_527	1300.138	464.1	63	1	836.038	64.304	-0.4213
CV_529	1580.8	442.32	57	1	1138.480	72.019	-0.3133
CV_530	416.15	79.85	42	2	336.300	80.812	-0.5998
CV_531	82.32	53.41	26	2	28.910	35.119	-0.7096
CV_532	65.48	20.66	63	1	44.820	68.448	-0.7039
CV_533	680.25	219.87	52	1	460.380	67.678	-0.5555
CV_535	9854.5	3488.27	24	1	6366.230	64.602	1.5541
CV_536	459.98	119.22	60	2	340.760	74.081	-0.5982
CV_537	446.79	436.2	33	1	10.590	2.370	-0.7161
CV_538	5682	1907.57	50	2	3774.430	66.428	0.6283
CV_539	838.18	203.37	62	2	634.810	75.737	-0.4932
CV_541	550.29	139.33	44	1	410.960	74.681	-0.5731
CV_542	5682	3324.94	43	1	2357.060	41.483	0.1220
CV_543	609.27	226.29	28	1	382.980	62.859	-0.5831
CV_544	6368.56	1750.99	45	1	4617.570	72.506	0.9295
CV_545	5682	4160.86	39	1	1521.140	26.771	-0.1766
CV_548	5208.03	1677.47	34	1	3530.560	67.791	0.5412
CV_549	490.24	142.78	46	1	347.460	70.875	-0.5958
CV_551	875	377.69	34	2	497.310	56.835	-0.5423
CV_553	617.64	190.29	29	2	427.350	69.191	-0.5673
CV_554	863.53	204.55	50	1	658.980	76.312	-0.4845
CV_556	5521.474	2112.39	49	2	3409.084	61.742	0.4978
CV_557	4588.052	1225.2	64	2	3362.852	73.296	0.4813
CV_558	3241.014	828.13	52	2	2412.884	74.448	0.1420
CV_559	5490.744	1629.24	57	2	3861.504	70.328	0.6594
CV_560	3261.272	1609.53	59	2	1651.742	50.647	-0.1299
CV_562	7498.708	2364.94	58	2	5133.768	68.462	1.1139
CV_563	8127.98	2359.39	63	2	5768.590	70.972	1.3406
CV_564	1184.316	361.59	50	2	822.726	69.468	-0.4260
CV_565	8109.276	3506.08	59	2	4603.196	56.765	0.9243
CV_566	2999.93	1580.5	62	2	1419.430	47.315	-0.2129
CV_567	4625.47	1674.89	50	1	2950.580	63.790	0.3340
CV_568	6764.67	3548.45	43	1	3216.220	47.544	0.4289
CV_569	718.79	292.45	60	1	426.340	59.314	-0.5676

CV_572	829.24	446.01	27	1	383.230	46.215	-0.5830
CV_573	10137.12	1860.37	64	1	8276.750	81.648	2.2365
CV_574	8769.03	2964.76	51	1	5804.270	66.191	1.3534
CV_575	3982.11	1227.35	53	1	2754.760	69.178	0.2641
CV_576	5121.95	1871.25	33	2	3250.700	63.466	0.4412
CV_577	6727.29	3057.8	51	1	3669.490	54.546	0.5908
CV_579	1843.87	726.61	49	1	1117.260	60.593	-0.3208
CV_580	2580.99	1069.74	53	1	1511.250	58.553	-0.1801
CV_581	5244.13	1192.74	43	1	4051.390	77.256	0.7272
CV_582	3650.04	1662.92	52	1	1987.120	54.441	-0.0101
CV_583	5682	2611.59	56	1	3070.410	54.037	0.3768
CV_584	6152.01	2675.54	32	2	3476.470	56.509	0.5219
